# Supplementary material for: Factors associated with COVID-19 vaccine uptake in people with kidney disease: an OpenSAFELY cohort study
Source: BMJ Open. 2023 Jan 31;13(1):e066164. doi: 10.1136/bmjopen-2022-066164 (PMC9890277; doi:10.1136/bmjopen-2022-066164)
Supplement: Supplementary data [file bmjopen-2022-066164supp001.pdf]

BMJ Publishing Group Limited (BMJ) disclaims all liability and responsibility arising from any reliance placed on this supplemental material which has been supplied by the author(s)

Supplementary Appendix

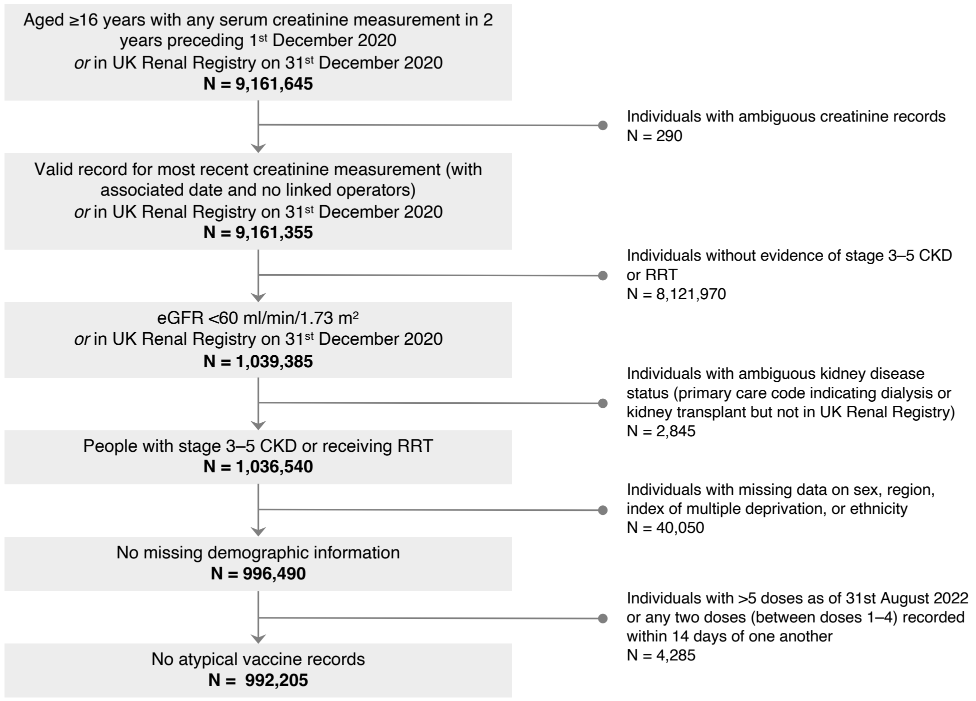

Supplementary Figure 1. Flow chart of study eligibility. Frequencies are rounded to the nearest 5. CKD, chronic kidney disease; eGFR, estimated glomerular filtrate rate; RRT, renal replacement therapy.

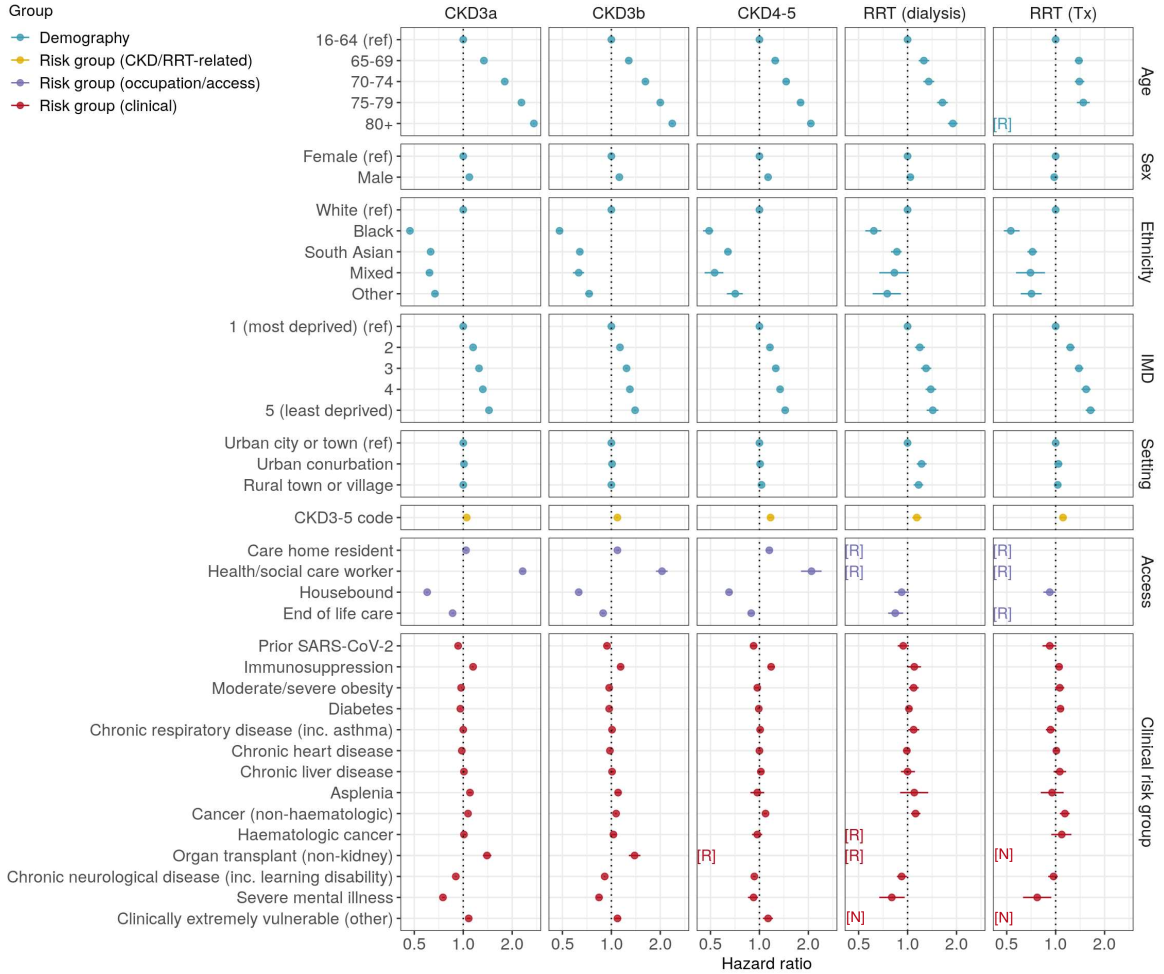

Supplementary Figure 2. Factors associated with completion of a 3-dose vaccine series in kidney disease subgroups. Hazard ratios and 95% confidence intervals were derived from partially adjusted models that included age, care home residence, health and social care worker status, housebound status, receipt of end-of-life care, setting (urban/rural), sex, ethnicity, IMD quintile, prior SARS-CoV-2 infection, immunosuppression, and haematologic cancer. Data are redacted [R] for any rows in which there were >0 and ≤10 event or non-event counts after rounding to the nearest 5, with non-event counts calculated among uncensored individuals. CKD, chronic kidney disease; IMD, index of multiple deprivation; [N], covariate absent in all individuals by definition; [R], redacted; RRT, renal replacement therapy; Tx, transplant.

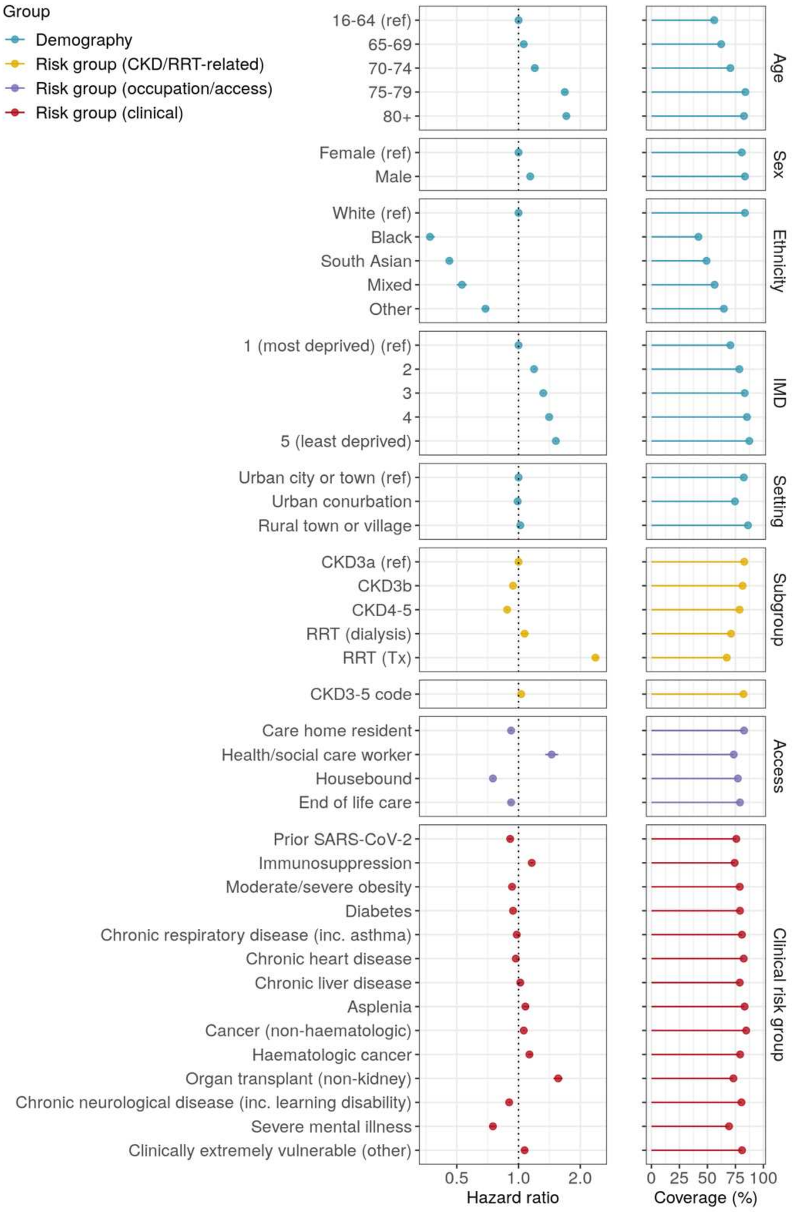

Supplementary Figure 3. Factors associated with completion of a 4-dose vaccine series in people with kidney disease. The left-hand panels display hazard ratios and 95% confidence intervals from partially adjusted models that included age, care home residence, health and social care worker status, housebound status, receipt of end-of-life care, setting (urban/rural), sex, ethnicity, IMD quintile, prior SARS-CoV-2 infection, immunosuppression, and haematologic cancer. Confidence intervals are generally too narrow to be visible. See **Supplementary Table 6** for minimally and fully adjusted model outputs. The right-hand panels display the cumulative coverage based on Kaplan-Meier estimates, censoring at death, deregistration, or 31st August 2022. CKD, chronic kidney disease; IMD, index of multiple deprivation; RRT, renal replacement therapy; Tx, transplant.

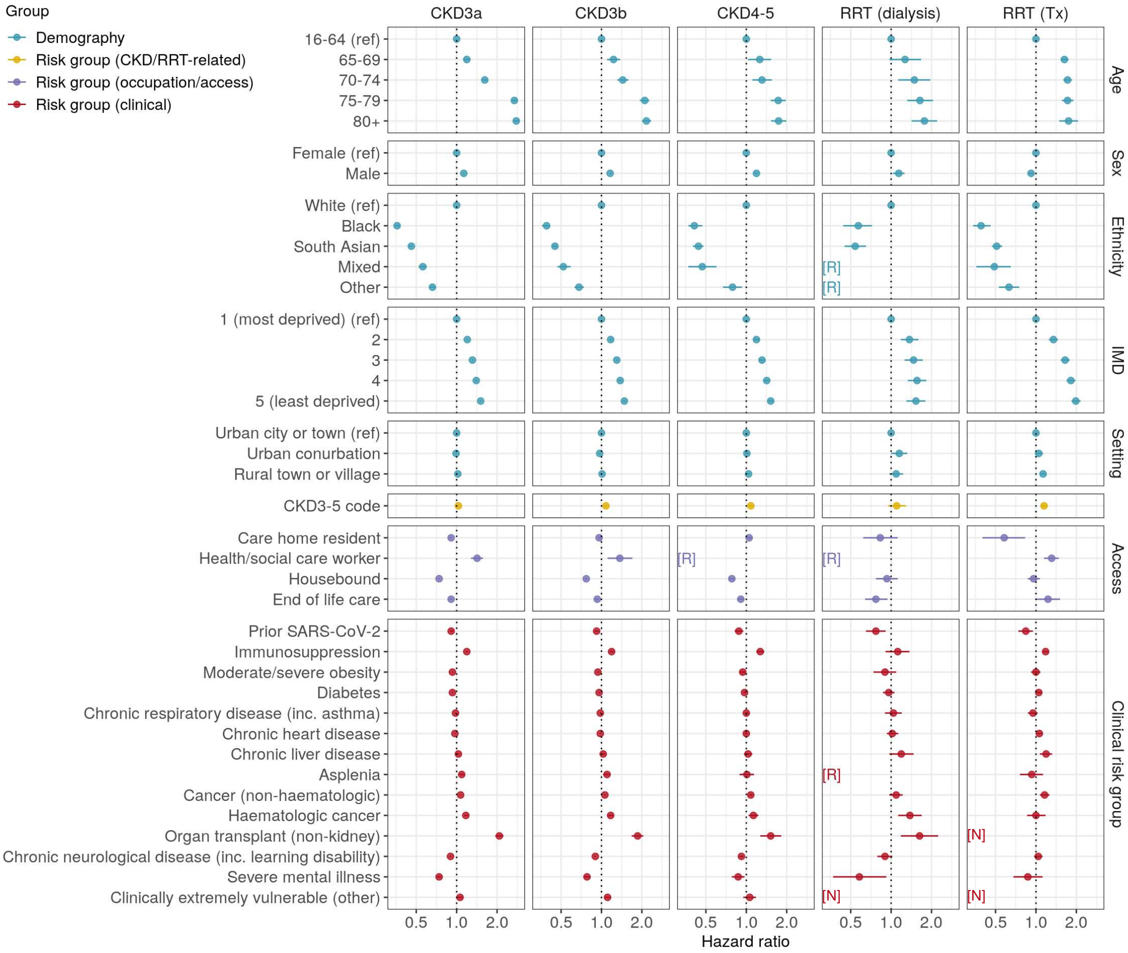

Supplementary Figure 4. Factors associated with completion of a 4-dose vaccine series in kidney disease subgroups. Individuals were included in the analysis if they were ≥75 years of age at baseline, care home residents, transplant recipients, or had a history of haematologic malignancy or immunosuppression. Hazard ratios and 95% confidence intervals were derived from partially adjusted models that included age, care home residence, health and social care worker status, housebound status, receipt of end-of-life care, setting (urban/rural), sex, ethnicity, IMD quintile, prior SARS-CoV-2 infection, immunosuppression, and haematologic cancer. Data are redacted [R] for any rows in which there were >0 and ≤10 event or non-event counts after rounding to the nearest 5, with non-event counts calculated among uncensored individuals. CKD, chronic kidney disease; IMD, index of multiple deprivation; [N], covariate absent in all individuals by definition; [R], redacted; RRT, renal replacement therapy; Tx, transplant.

, *et al. BMJ Open* 2023; 13:e066164. doi: 10.1136/bmjopen-2022-066164

Supplementary Table 1. Product profile by dose.

Subgroup	Dose 1, n (%)	Dose 2, n (%)	Dose 3, n (%)	Dose 4, n (%)
**N**	944,605 (100)	922,365 (100)	849,150 (100)	535,975 (100)
**Product**				
BNT	504,980 (53.5)	495,375 (53.7)	779,740 (91.8)	216,435 (40.4)
AZ	438,890 (46.5)	425,820 (46.2)	1,965 (0.2)	200 (0.0)
MOD	735 (0.1)	1,170 (0.1)	67,445 (7.9)	319,340 (59.6)
**2-dose product combination**				
BNT-BNT	–	492,105 (53.4)	–	–
AZ-AZ	–	422,145 (45.8)	–	–
BNT-AZ	–	3,675 (0.4)	–	–
AZ-BNT	–	3,215 (0.3)	–	–
Other	–	1,225 (0.1)	–	–
**3-dose product combination**				
BNT-BNT-BNT	–	–	438,905 (51.7)	–
AZ-AZ-BNT	–	–	336,725 (39.7)	–
AZ-AZ-MOD	–	–	44,430 (5.2)	–
BNT-BNT-MOD	–	–	21,970 (2.6)	–
BNT-AZ-BNT	–	–	2,575 (0.3)	–
AZ-AZ-AZ	–	–	1,720 (0.2)	–
AZ-BNT-BNT	–	–	1,320 (0.2)	–
Other	–	–	1,510 (0.2)	–

Frequencies are rounded to the nearest 5. Any product combinations given to <1000 individuals were pooled as 'Other'. AZ, ChAdOx1-S (AstraZeneca); BNT, BNT162b2 (Pfizer-BioNTech); MOD, mRNA-1273 (Moderna).

, et al. BMJ Open 2023; 13:e066164. doi: 10.1136/bmjopen-2022-066164

Supplementary Table 2. Cox proportional hazards model and logistic regression outputs for factors associated with completion of a 3-dose vaccination series in people with kidney disease.

	Cox models					Logistic regression	
**Variable**	**N (n events)**	**Cov. (%)**	**HR (95% CI), minimally adjusted**	**HR (95% CI), partially adjusted**	**HR (95% CI), fully adjusted**	**N (n events)**	**OR (95% CI), partially adjusted**
**Age**							
16–64 (ref)	109,815 (89,610)	84.7	1.00	1.00	1.00	103,215 (87,745)	1.00
65–69	74,740 (65,845)	91.9	1.32 (1.31-1.34)	1.28 (1.27-1.29)	1.31 (1.30-1.32)	69,495 (64,110)	1.85 (1.79-1.92)
70–74	143,575 (129,880)	94.8	1.76 (1.75-1.78)	1.67 (1.66-1.69)	1.71 (1.70-1.73)	132,385 (125,905)	2.67 (2.59-2.75)
75–79	181,200 (163,730)	95.8	2.20 (2.18-2.21)	2.09 (2.07-2.11)	2.15 (2.14-2.17)	162,940 (156,740)	3.38 (3.27-3.49)
80+	482,870 (399,765)	95.5	2.47 (2.45-2.49)	2.45 (2.44-2.47)	2.56 (2.54-2.58)	370,940 (356,715)	3.39 (3.30-3.48)
**Sex**							
Female (ref)	555,365 (476,420)	93.7	1.00	1.00	1.00	473,535 (445,645)	1.00
Male	436,840 (372,415)	94.1	1.11 (1.11-1.12)	1.10 (1.09-1.10)	1.10 (1.09-1.10)	365,440 (345,565)	1.18 (1.16-1.20)
**Ethnicity**							
White (ref)	930,565 (805,890)	95.1	1.00	1.00	1.00	785,470 (750,345)	1.00
Black	17,675 (11,030)	66.8	0.45 (0.44-0.46)	0.48 (0.47-0.48)	0.48 (0.47-0.49)	15,705 (10,590)	0.19 (0.19-0.20)
South Asian	32,620 (23,560)	79.2	0.61 (0.60-0.62)	0.65 (0.64-0.66)	0.65 (0.64-0.66)	28,000 (22,365)	0.32 (0.31-0.33)
Mixed	4,755 (3,435)	77.5	0.60 (0.58-0.62)	0.62 (0.60-0.64)	0.62 (0.60-0.65)	4,215 (3,290)	0.29 (0.26-0.31)
Other	6,590 (4,920)	82.2	0.68 (0.66-0.70)	0.70 (0.68-0.72)	0.69 (0.67-0.71)	5,585 (4,625)	0.33 (0.31-0.36)
**IMD**							
1 most deprived (ref)	164,415 (130,595)	88.1	1.00	1.00	1.00	136,680 (121,160)	1.00
2	187,240 (156,820)	92.5	1.19 (1.18-1.20)	1.15 (1.14-1.16)	1.14 (1.14-1.15)	157,145 (146,105)	1.48 (1.44-1.52)
3	224,590 (194,255)	94.8	1.32 (1.31-1.33)	1.25 (1.24-1.26)	1.24 (1.23-1.25)	190,175 (181,085)	1.90 (1.84-1.95)
4	216,980 (190,310)	95.8	1.40 (1.39-1.42)	1.32 (1.31-1.33)	1.31 (1.30-1.32)	184,545 (177,440)	2.18 (2.11-2.25)
5 least deprived	198,985 (176,855)	96.7	1.53 (1.52-1.54)	1.44 (1.43-1.45)	1.42 (1.41-1.43)	170,425 (165,420)	2.74 (2.64-2.83)
**Setting**							
Urban city or town (ref)	530,975 (456,140)	94.2	1.00	1.00	1.00	449,315 (425,415)	1.00
Urban conurbation	204,530 (166,040)	89.8	0.93 (0.93-0.94)	1.02 (1.01-1.02)	1.01 (1.01-1.02)	170,720 (154,285)	0.84 (0.82-0.86)
Rural town or village	256,700 (226,650)	96.3	1.08 (1.07-1.09)	1.01 (1.00-1.01)	1.01 (1.00-1.01)	218,940 (211,515)	1.18 (1.14-1.21)
**Kidney disease subgroup**							
CKD3a (ref)	659,785 (579,655)	94.1	1.00	1.00	1.00	581,900 (549,290)	1.00
CKD3b	245,380 (203,490)	94.3	0.92 (0.92-0.93)	0.94 (0.93-0.94)	0.93 (0.92-0.93)	194,630 (184,545)	0.95 (0.93-0.97)
CKD4–5	64,855 (47,985)	92.3	0.84 (0.83-0.85)	0.87 (0.87-0.88)	0.86 (0.85-0.87)	44,025 (41,015)	0.87 (0.83-0.90)
RRT (dialysis)	9,455 (6,925)	86.8	1.03 (1.00-1.05)	1.11 (1.08-1.14)	1.09 (1.06-1.11)	6,875 (6,015)	1.13 (1.04-1.22)
RRT (Tx)	12,735 (10,780)	89.1	1.53 (1.50-1.56)	1.51 (1.48-1.54)	1.48 (1.45-1.51)	11,545 (10,340)	1.23 (1.15-1.31)
**Primary care coding of kidney disease**							
CKD diagnostic code	592,520 (507,470)	94.5	1.03 (1.02-1.03)	1.04 (1.04-1.04)	1.07 (1.06-1.07)	494,910 (469,965)	1.25 (1.23-1.28)
**Risk group (occupation/access)**							
Care home resident	44,470 (28,155)	95.9	1.02 (1.00-1.03)	1.06 (1.05-1.08)	1.11 (1.10-1.13)	21,100 (20,320)	1.25 (1.16-1.35)
Health/social care worker	5,680 (5,245)	93.5	2.12 (2.06-2.18)	2.26 (2.20-2.32)	2.24 (2.18-2.30)	5,515 (5,165)	2.67 (2.38-2.99)
Housebound	54,600 (38,340)	93.4	0.60 (0.59-0.60)	0.61 (0.61-0.62)	0.63 (0.62-0.63)	33,090 (31,195)	0.85 (0.81-0.90)

, *et al. BMJ Open* 2023; 13:e066164. doi: 10.1136/bmjopen-2022-066164

End of life care	40,400 (24,220)	93.5	0.82 (0.81-0.84)	0.87 (0.86-0.88)	0.87 (0.86-0.88)	19,445 (18,405)	0.89 (0.83-0.95)
**Risk group (clinical)**							
Prior SARS-CoV-2	27,835 (19,630)	91.7	0.88 (0.87-0.90)	0.93 (0.92-0.94)	0.94 (0.92-0.95)	18,470 (17,040)	1.02 (0.96-1.08)
Immunosuppression	56,985 (47,850)	94.6	1.17 (1.15-1.18)	1.19 (1.18-1.20)	1.13 (1.11-1.14)	46,000 (43,730)	1.47 (1.39-1.54)
Moderate/severe obesity	111,630 (96,740)	92.8	0.93 (0.93-0.94)	0.97 (0.96-0.97)	0.98 (0.97-0.99)	99,230 (92,410)	1.10 (1.07-1.13)
Diabetes	275,745 (229,435)	93.1	0.91 (0.90-0.91)	0.96 (0.95-0.96)	0.97 (0.96-0.97)	225,340 (211,050)	1.09 (1.06-1.11)
CRD (inc. asthma)	120,935 (98,590)	94.5	0.98 (0.97-0.98)	1.00 (0.99-1.01)	1.00 (1.00-1.01)	92,980 (88,445)	1.08 (1.04-1.11)
Chronic heart disease	438,965 (366,070)	94.8	0.98 (0.98-0.99)	0.98 (0.98-0.98)	0.99 (0.98-0.99)	348,165 (332,040)	1.08 (1.05-1.10)
Chronic liver disease	32,215 (27,010)	92.5	1.00 (0.99-1.01)	1.01 (1.00-1.02)	1.01 (1.00-1.02)	27,140 (25,240)	1.04 (0.99-1.09)
Asplenia	8,665 (7,440)	94.5	1.10 (1.07-1.12)	1.09 (1.06-1.11)	1.08 (1.06-1.11)	7,345 (6,970)	1.18 (1.06-1.31)
Cancer (non-haematologic)	168,840 (141,125)	95.8	1.09 (1.08-1.10)	1.07 (1.06-1.08)	1.07 (1.07-1.08)	133,245 (128,420)	1.34 (1.30-1.38)
Haematologic cancer	20,430 (16,740)	95.8	1.16 (1.14-1.18)	0.98 (0.96-1.00)	1.03 (1.01-1.05)	15,450 (14,895)	1.04 (0.94-1.15)
Organ transplant (non-kidney)	2,190 (1,895)	94.3	1.45 (1.38-1.51)	1.36 (1.30-1.43)	1.41 (1.35-1.48)	1,880 (1,780)	1.68 (1.36-2.07)
CND (inc. learning disability)	163,135 (129,130)	94.1	0.89 (0.88-0.89)	0.90 (0.90-0.91)	0.91 (0.90-0.91)	119,585 (113,255)	0.92 (0.90-0.95)
Severe mental illness	12,985 (10,105)	87.3	0.75 (0.73-0.76)	0.78 (0.76-0.80)	0.79 (0.77-0.80)	10,575 (9,280)	0.65 (0.61-0.69)
CEV (other)	9,065 (7,790)	92.8	1.04 (1.02-1.06)	1.08 (1.06-1.10)	1.06 (1.04-1.09)	7,795 (7,265)	1.02 (0.93-1.12)

Cumulative coverage was determined based on Kaplan-Meier estimates, censoring at death, deregistration, or 31st August 2022. Logistic regression models included 838,975 individuals who were uncensored (registered and alive) as of the analysis cut-off (31st August 2022). Minimally adjusted models included age, care home residence, and health and social care worker status given their use in vaccine prioritisation criteria. Partially adjusted models additionally included housebound status, receipt of end-of-life care, setting, sex, ethnicity, IMD quintile, prior SARS-CoV-2 infection, immunosuppression, and haematologic cancer. Fully adjusted models included all covariates. CI, confidence interval; CKD, chronic kidney disease; HR, hazard ratio; IMD, index of multiple deprivation; OR, odds ratio; RRT, renal replacement therapy; Tx, transplant.

Supplementary Table 3. Cox proportional hazards models for factors associated with completion of a 3-dose vaccination series in kidney disease subgroups.

	CKD3a		CKD3b		CKD4-5		RRT (dialysis)		RRT (Tx)	
**Variable**	**Cov. (%)**	**HR (95% CI)**	**Cov. (%)**	**HR (95% CI)**	**Cov. (%)**	**HR (95% CI)**	**Cov. (%)**	**HR (95% CI)**	**Cov. (%)**	**HR (95% CI)**
**Age**										
16–64 (ref)	85.1	1.00	83.1	1.00	81.4	1.00	80.8	1.00	87.2	1.00
65–69	92.3	1.34 (1.32-1.35)	90.8	1.28 (1.24-1.31)	88.9	1.25 (1.19-1.32)	88.7	1.26 (1.17-1.36)	94.1	1.39 (1.31-1.47)
70–74	95.1	1.80 (1.78-1.82)	94.1	1.62 (1.58-1.66)	91.9	1.46 (1.40-1.52)	91.2	1.35 (1.25-1.46)	94.5	1.40 (1.31-1.50)
75–79	96.0	2.28 (2.25-2.30)	95.4	2.00 (1.96-2.05)	94.0	1.79 (1.72-1.86)	95.0	1.64 (1.52-1.77)	94.3	1.48 (1.35-1.62)
80+	95.7	2.72 (2.70-2.75)	95.4	2.37 (2.32-2.41)	94.3	2.07 (2.00-2.13)	95.0	1.90 (1.77-2.03)	[R]	[R]
**Sex**										
Female (ref)	93.9	1.00	94.0	1.00	92.1	1.00	85.4	1.00	89.2	1.00
Male	94.3	1.09 (1.08-1.09)	94.6	1.12 (1.11-1.13)	92.5	1.13 (1.11-1.15)	87.6	1.04 (0.99-1.09)	89.0	0.98 (0.94-1.02)
**Ethnicity**										
White (ref)	95.2	1.00	95.3	1.00	94.1	1.00	89.5	1.00	91.6	1.00
Black	66.0	0.47 (0.46-0.48)	68.6	0.48 (0.46-0.50)	66.0	0.49 (0.45-0.52)	70.2	0.62 (0.55-0.69)	69.9	0.53 (0.48-0.60)
South Asian	79.6	0.63 (0.62-0.64)	78.7	0.64 (0.63-0.66)	75.3	0.64 (0.61-0.67)	81.6	0.86 (0.79-0.92)	80.2	0.72 (0.67-0.77)
Mixed	77.3	0.62 (0.59-0.64)	79.2	0.63 (0.58-0.68)	69.7	0.53 (0.46-0.60)	81.8	0.83 (0.67-1.02)	79.2	0.70 (0.57-0.86)
Other	82.3	0.67 (0.65-0.70)	83.6	0.73 (0.69-0.77)	79.3	0.71 (0.63-0.79)	70.1	0.75 (0.61-0.91)	80.3	0.71 (0.61-0.82)
**IMD**										
1 most deprived (ref)	88.3	1.00	89.1	1.00	86.0	1.00	79.6	1.00	81.2	1.00
2	92.6	1.15 (1.14-1.16)	93.1	1.13 (1.11-1.14)	90.8	1.16 (1.12-1.19)	85.3	1.19 (1.11-1.28)	86.7	1.23 (1.16-1.31)
3	94.9	1.25 (1.24-1.26)	95.2	1.24 (1.22-1.26)	93.4	1.26 (1.22-1.30)	89.1	1.30 (1.21-1.40)	90.9	1.39 (1.31-1.48)
4	95.9	1.32 (1.31-1.33)	96.0	1.30 (1.28-1.32)	95.0	1.34 (1.30-1.38)	92.3	1.39 (1.29-1.50)	92.7	1.54 (1.44-1.64)
5 least deprived	96.8	1.44 (1.43-1.45)	96.8	1.40 (1.38-1.42)	96.0	1.44 (1.39-1.48)	93.1	1.43 (1.31-1.55)	94.6	1.64 (1.53-1.75)
**Setting**										
Urban city or town (ref)	94.4	1.00	94.6	1.00	93.0	1.00	86.5	1.00	89.9	1.00
Urban conurbation	90.2	1.01 (1.01-1.02)	90.5	1.01 (1.00-1.02)	87.1	1.01 (0.98-1.03)	83.1	1.22 (1.14-1.31)	84.0	1.04 (0.98-1.10)
Rural town or village	96.4	1.00 (1.00-1.01)	96.5	1.00 (0.99-1.01)	95.6	1.03 (1.00-1.05)	94.1	1.17 (1.09-1.25)	93.5	1.03 (0.98-1.08)
**Primary care coding of kidney disease**										
CKD diagnostic code	94.9	1.05 (1.04-1.06)	94.7	1.09 (1.07-1.10)	92.8	1.17 (1.13-1.20)	88.1	1.14 (1.07-1.22)	90.1	1.11 (1.06-1.16)
**Risk group (occupation/access)**										
Care home resident	95.9	1.04 (1.02-1.06)	96.1	1.09 (1.07-1.12)	94.9	1.15 (1.10-1.20)	[R]	[R]	[R]	[R]
Health/social care worker	93.7	2.32 (2.25-2.40)	91.9	2.05 (1.88-2.22)	90.2	2.09 (1.80-2.41)	[R]	[R]	[R]	[R]
Housebound	93.6	0.60 (0.59-0.61)	93.7	0.63 (0.62-0.65)	92.0	0.65 (0.63-0.68)	90.5	0.92 (0.83-1.02)	91.2	0.92 (0.84-1.00)
End of life care	93.7	0.86 (0.85-0.88)	93.9	0.89 (0.87-0.91)	92.6	0.89 (0.85-0.93)	86.5	0.84 (0.76-0.94)	[R]	[R]
**Risk group (clinical)**										
Prior SARS-CoV-2	92.3	0.93 (0.91-0.94)	92.7	0.94 (0.91-0.96)	89.8	0.92 (0.88-0.97)	83.2	0.94 (0.87-1.02)	83.4	0.92 (0.83-1.01)
Immunosuppression	95.4	1.15 (1.13-1.16)	95.5	1.14 (1.11-1.17)	93.6	1.18 (1.12-1.24)	86.9	1.10 (1.00-1.21)	89.4	1.05 (1.00-1.09)
Moderate/severe obesity	92.8	0.97 (0.97-0.98)	93.2	0.97 (0.96-0.99)	91.4	0.97 (0.94-1.00)	88.2	1.09 (1.02-1.17)	88.7	1.06 (0.99-1.13)
Diabetes	93.3	0.96 (0.95-0.96)	93.4	0.97 (0.96-0.98)	91.7	0.99 (0.97-1.01)	88.1	1.02 (0.97-1.07)	90.1	1.07 (1.03-1.12)
CRD (inc. asthma)	94.6	1.00 (0.99-1.00)	94.8	1.01 (1.00-1.02)	93.2	1.01 (0.98-1.04)	92.0	1.09 (1.01-1.18)	89.3	0.93 (0.87-1.00)
Chronic heart disease	95.1	0.98 (0.98-0.99)	94.9	0.98 (0.97-0.99)	93.5	1.00 (0.98-1.02)	88.3	0.99 (0.94-1.04)	90.6	1.01 (0.97-1.05)
Chronic liver disease	92.7	1.01 (1.00-1.03)	92.8	1.01 (0.99-1.04)	91.3	1.02 (0.97-1.07)	83.3	1.00 (0.91-1.11)	89.9	1.06 (0.97-1.16)
Asplenia	95.1	1.10 (1.07-1.13)	94.4	1.10 (1.05-1.15)	90.2	0.97 (0.88-1.07)	84.4	1.10 (0.90-1.34)	87.3	0.95 (0.81-1.12)
Cancer (non-haematologic)	95.9	1.07 (1.07-1.08)	96.0	1.07 (1.05-1.08)	94.8	1.09 (1.06-1.11)	92.3	1.12 (1.05-1.20)	93.7	1.14 (1.06-1.22)
Haematologic cancer	95.9	1.01 (0.99-1.04)	95.8	1.03 (0.99-1.07)	94.1	0.97 (0.90-1.04)	[R]	[R]	90.3	1.09 (0.94-1.25)
Organ transplant (non-kidney)	93.4	1.40 (1.32-1.49)	94.4	1.39 (1.28-1.51)	[R]	[R]	[R]	[R]	[N]	[N]
CND (inc. learning disability)	94.3	0.90 (0.89-0.91)	94.3	0.91 (0.90-0.92)	92.6	0.93 (0.91-0.95)	87.5	0.92 (0.86-0.99)	90.9	0.97 (0.90-1.03)
Severe mental illness	86.2	0.75 (0.74-0.77)	90.6	0.84 (0.81-0.87)	87.7	0.92 (0.85-0.98)	73.3	0.80 (0.67-0.96)	76.6	0.77 (0.63-0.94)

CEV (other)	93.1	1.08 (1.05-1.11)	93.6	1.09 (1.05-1.15)	89.4	1.13 (1.05-1.21)	[N]	[N]	[N]	[N]

Counts are rounded to the nearest 5. Cumulative coverage was determined based on Kaplan-Meier estimates, censoring at death, deregistration, or 31st August 2022. Hazard ratios and 95% confidence intervals were derived from partially adjusted models that included age, care home residence, health and social care worker status, housebound status, receipt of end-of-life care, setting (urban/rural), sex, ethnicity, IMD quintile, prior SARS-CoV-2 infection, immunosuppression, and haematologic cancer. Data are redacted [R] for any rows in which there were >0 and ≤10 event or non-event counts after rounding to the nearest 5, with non-event counts calculated among uncensored individuals. CEV, clinically extremely vulnerable; CKD, chronic kidney disease; CND, chronic neurological disease; IMD, index of multiple deprivation; [N], covariate absent in all individuals by definition; [R] redacted; RRT, renal replacement therapy; Tx, transplant.

, *et al. BMJ Open* 2023; 13:e066164. doi: 10.1136/bmjopen-2022-066164

Supplementary Table 4. Cumulative 3-dose coverage in subgroups defined by deprivation index, ethnicity, age, and kidney disease severity.

	IMD 1 (most deprived)				IMD 2				IMD 3				IMD 4				IMD 5 (least deprived)			
	White		Minority ethnic groups		White		Minority ethnic groups		White		Minority ethnic groups		White		Minority ethnic groups		White		Minority ethnic groups	
**Characteristic**	N	%	N	%	N	%	N	%	N	%	N	%	N	%	N	%	N	%	N	%
**All**	143510	91.3	20905	66.9	171190	94.1	16045	75.8	213100	95.6	11485	80.4	209385	96.2	7590	84.1	193375	97.0	5610	87.7
**Age**																				
16–64	19850	81.3	6655	59.6	19170	86.4	4720	67.5	19665	89.3	3075	72.0	17945	90.9	1970	76.4	15395	93.4	1375	81.5
65–69	12720	89.5	2560	67.0	13520	92.9	1875	75.1	15135	94.4	1275	79.1	14210	95.2	830	85.8	12005	95.9	610	86.6
70–74	22120	92.3	2425	71.5	25420	94.9	2015	79.4	31215	96.1	1535	84.2	30175	96.5	1015	87.0	26870	97.1	790	89.6
75–79	25380	93.6	2475	73.3	31565	95.6	2175	81.8	40160	96.8	1690	84.3	39405	97.4	1140	86.6	36295	97.9	920	90.3
80+	63440	94.1	6790	70.2	81520	95.4	5260	79.8	106935	96.4	3915	83.9	107650	96.8	2635	86.7	102815	97.4	1920	89.4
**Kidney disease subgroup**																				
CKD3a	92270	91.4	12630	66.3	112400	94.2	9790	75.2	142175	95.7	7210	79.8	141890	96.3	4890	83.4	132825	97.1	3710	87.4
CKD3b	37355	92.0	4675	67.0	43600	94.4	3620	77.6	53440	95.9	2530	81.7	51140	96.4	1655	85.1	46200	97.0	1170	88.1
CKD4-5	10320	90.3	1945	64.0	11610	93.1	1380	72.8	13620	94.6	980	78.9	12815	95.5	545	82.5	11245	96.3	400	85.7
RRT (dial.)	1770	83.1	850	73.2	1580	88.6	605	75.8	1540	90.8	350	81.7	1330	92.3	205	85.7	1085	93.6	140	83.8
RRT (Tx)	1800	85.2	805	72.3	2000	89.2	650	78.7	2330	92.4	415	81.7	2215	93.9	305	82.1	2015	95.1	200	85.9

Cumulative coverage (%) was determined based on Kaplan-Meier estimates, censoring at death, deregistration, or 31st August 2022. Minority ethnic groups are combined due to their low combined prevalence within the study population. CKD, chronic kidney disease; dial., dialysis; IMD, index of multiple deprivation quintile; RRT, renal replacement therapy; Tx, transplant.

Key

	<75%
	75–90%

, et al. BMJ Open 2023; 13:e066164. doi: 10.1136/bmjopen-2022-066164

Supplementary Table 5. Baseline characteristics of secondary outcome analysis population (dose 4 uptake).

Characteristic	All, n (%)	CKD3a, n (%)	CKD3b, n (%)	CKD4–5, n (%)	RRT (dialysis), n (%)	RRT (Tx), n (%)
**N**	698,755 (100)	431,830 (100)	199,175 (100)	51,305 (100)	3,715 (100)	12,735 (100)
**Age**						
16–64	17,035 (2.4)	5,170 (1.2)	1,405 (0.7)	565 (1.1)	615 (16.6)	9,285 (72.9)
65–69	6,630 (0.9)	3,660 (0.8)	1,075 (0.5)	335 (0.7)	145 (3.9)	1,415 (11.1)
70–74	11,015 (1.6)	6,850 (1.6)	2,200 (1.1)	650 (1.3)	145 (3.9)	1,170 (9.2)
75–79	181,200 (25.9)	130,805 (30.3)	39,940 (20.1)	8,620 (16.8)	1,225 (33.0)	615 (4.8)
80+	482,870 (69.1)	285,345 (66.1)	154,560 (77.6)	41,135 (80.2)	1,590 (42.8)	245 (1.9)
**Sex**						
Female	398,820 (57.1)	248,700 (57.6)	116,330 (58.4)	27,545 (53.7)	1,335 (35.9)	4,910 (38.6)
Male	299,940 (42.9)	183,130 (42.4)	82,845 (41.6)	23,755 (46.3)	2,385 (64.2)	7,825 (61.4)
**Ethnicity**						
White	665,365 (95.2)	413,335 (95.7)	190,370 (95.6)	48,230 (94.0)	3,080 (82.9)	10,355 (81.3)
Black	8,610 (1.2)	4,910 (1.1)	2,240 (1.1)	790 (1.5)	185 (5.0)	490 (3.8)
South Asian	18,360 (2.6)	9,860 (2.3)	4,880 (2.5)	1,770 (3.4)	360 (9.7)	1,490 (11.7)
Mixed	2,455 (0.4)	1,455 (0.3)	645 (0.3)	195 (0.4)	35 (0.9)	125 (1.0)
Other	3,965 (0.6)	2,270 (0.5)	1,045 (0.5)	320 (0.6)	55 (1.5)	275 (2.2)
**Index of multiple deprivation quintile**						
1 most deprived	105,175 (15.1)	61,455 (14.2)	31,565 (15.8)	8,785 (17.1)	765 (20.6)	2,605 (20.5)
2	127,700 (18.3)	76,800 (17.8)	37,380 (18.8)	10,065 (19.6)	805 (21.7)	2,650 (20.8)
3	160,310 (22.9)	99,115 (23.0)	45,905 (23.0)	11,735 (22.9)	805 (21.7)	2,750 (21.6)
4	157,740 (22.6)	99,470 (23.0)	44,055 (22.1)	10,980 (21.4)	720 (19.4)	2,515 (19.7)
5 least deprived	147,835 (21.2)	94,985 (22.0)	40,275 (20.2)	9,740 (19.0)	620 (16.7)	2,210 (17.4)
**Setting**						
Urban city or town	373,270 (53.4)	229,995 (53.3)	107,200 (53.8)	27,600 (53.8)	1,875 (50.5)	6,605 (51.9)
Urban conurbation	140,300 (20.1)	85,065 (19.7)	39,865 (20.0)	10,885 (21.2)	1,010 (27.2)	3,470 (27.2)
Rural	185,185 (26.5)	116,770 (27.0)	52,110 (26.2)	12,820 (25.0)	830 (22.3)	2,655 (20.8)
**Primary care coding of kidney disease**						
CKD3–5 diagnostic code	439,595 (62.9)	223,695 (51.8)	157,215 (78.9)	45,745 (89.2)	3,290 (88.6)	9,650 (75.8)
Dialysis code	11,280 (1.6)	[N]	[N]	[N]	2,980 (80.2)	8,300 (65.2)
Kidney transplant code	12,780 (1.8)	[N]	[N]	[N]	440 (11.8)	12,340 (96.9)
**Risk group (occupation/access)**						
Care home resident	44,470 (6.4)	24,280 (5.6)	15,255 (7.7)	4,705 (9.2)	165 (4.4)	65 (0.5)
Health/social care worker	1,040 (0.1)	515 (0.1)	110 (0.1)	30 (0.1)	[R]	370 (2.9)
Housebound	50,195 (7.2)	24,770 (5.7)	18,040 (9.1)	6,445 (12.6)	330 (8.9)	610 (4.8)
End of life care	36,515 (5.2)	18,305 (4.2)	12,585 (6.3)	5,110 (10.0)	335 (9.0)	180 (1.4)
**Risk group (clinical)**						
Prior SARS-CoV-2 *	20,685 (3.0)	10,845 (2.5)	6,590 (3.3)	2,295 (4.5)	415 (11.2)	535 (4.2)
Immunosuppression	56,985 (8.2)	33,045 (7.7)	13,600 (6.8)	4,000 (7.8)	985 (26.5)	5,355 (42.0)
Moderate/severe obesity	53,820 (7.7)	31,075 (7.2)	16,655 (8.4)	4,680 (9.1)	260 (7.0)	1,150 (9.0)
Diabetes	190,955 (27.3)	100,935 (23.4)	63,425 (31.8)	21,350 (41.6)	1,405 (37.8)	3,840 (30.2)
Chronic respiratory disease (inc. asthma)	92,340 (13.2)	55,060 (12.8)	28,215 (14.2)	7,570 (14.8)	505 (13.6)	990 (7.8)

Chronic heart disease	347,840 (49.8)	200,060 (46.3)	110,430 (55.4)	31,365 (61.1)	2,140 (57.6)	3,850 (30.2)
Chronic liver disease	18,560 (2.7)	11,035 (2.6)	5,265 (2.6)	1,480 (2.9)	200 (5.4)	585 (4.6)
Asplenia	5,960 (0.9)	3,690 (0.9)	1,585 (0.8)	455 (0.9)	55 (1.5)	170 (1.3)
Cancer (non-haematologic)	130,945 (18.7)	79,045 (18.3)	39,060 (19.6)	11,145 (21.7)	730 (19.7)	960 (7.5)
Haematologic cancer	20,430 (2.9)	12,180 (2.8)	5,830 (2.9)	1,875 (3.7)	310 (8.3)	240 (1.9)
Organ transplant (non-kidney) †	2,190 (0.3)	1,205 (0.3)	690 (0.3)	220 (0.4)	75 (2.0)	[N]
CND (inc. learning disability)	135,005 (19.3)	79,505 (18.4)	42,145 (21.2)	11,520 (22.5)	655 (17.6)	1,180 (9.3)
Severe mental illness	7,075 (1.0)	4,185 (1.0)	2,070 (1.0)	640 (1.2)	55 (1.5)	125 (1.0)
Clinically extremely vulnerable (other) §	5,850 (0.8)	3,585 (0.8)	1,690 (0.8)	580 (1.1)	[N]	[N]
**Region**						
East of England	156,055 (22.3)	97,360 (22.5)	43,865 (22.0)	10,950 (21.3)	910 (24.5)	2,970 (23.3)
Midlands	155,545 (22.3)	95,935 (22.2)	44,660 (22.4)	11,410 (22.2)	825 (22.2)	2,715 (21.3)
London	20,295 (2.9)	11,830 (2.7)	5,525 (2.8)	1,775 (3.5)	255 (6.9)	905 (7.1)
North East and Yorkshire	131,475 (18.8)	80,280 (18.6)	38,150 (19.2)	9,910 (19.3)	675 (18.2)	2,465 (19.4)
North West	63,585 (9.1)	39,165 (9.1)	18,285 (9.2)	4,735 (9.2)	235 (6.3)	1,160 (9.1)
South East	43,760 (6.3)	27,525 (6.4)	12,125 (6.1)	3,135 (6.1)	235 (6.3)	735 (5.8)
South West	128,045 (18.3)	79,730 (18.5)	36,560 (18.4)	9,385 (18.3)	585 (15.7)	1,780 (14.0)

Individuals were included in the analysis if they were ≥75 years of age at baseline, care home residents, transplant recipients, or had a history of haematologic malignancy or immunosuppression. Data are n (%) after rounding to the nearest 5. Primary care codes and risk groups are coded by separate binary variables; percentages under these table subheadings therefore do not sum to 100. Among people with CKD4–5, 47,670 had stage 4 CKD (eGFR 15–29 ml/min/1.73 m^2) and 3,630 had stage 5 CKD (eGFR <15ml/min/1.73 m^2). CKD, chronic kidney disease; CND, chronic neurological disease; [N], covariate absent in all individuals by definition; RRT, renal replacement therapy; Tx, transplant. * Based on prior evidence of a positive SARS-CoV-2 test, COVID-19-related primary care code, or COVID-19-related hospitalisation as of 1st December 2020; † Excludes individuals with kidney transplants based on primary care coding or UK Renal Registry status; § Classified as clinically extremely vulnerable in the absence of any of the comorbidities listed above (including RRT).

, et al. BMJ Open 2023; 13:e066164. doi: 10.1136/bmjopen-2022-066164

Supplementary Table 6. Cox proportional hazards models for factors associated with completion of a 4-dose vaccination series in people with kidney disease.

Variable	N (n events)	Cov. (%)	HR (95% CI), minimally adjusted	HR (95% CI), partially adjusted	HR (95% CI), fully adjusted
**Age**					
16–64 (ref)	17,035 (8,955)	56.1	1.00	1.00	1.00
65–69	6,630 (3,685)	62.3	1.18 (1.14-1.23)	1.06 (1.02-1.10)	1.53 (1.47-1.60)
70–74	11,015 (6,750)	70.5	1.41 (1.37-1.45)	1.20 (1.16-1.24)	1.89 (1.82-1.96)
75–79	181,200 (139,245)	83.9	1.71 (1.67-1.74)	1.68 (1.64-1.72)	2.96 (2.87-3.06)
80+	482,870 (321,255)	82.7	1.70 (1.67-1.74)	1.71 (1.67-1.75)	3.04 (2.94-3.13)
**Sex**					
Female (ref)	398,820 (272,245)	80.7	1.00	1.00	1.00
Male	299,940 (207,645)	83.5	1.15 (1.15-1.16)	1.14 (1.13-1.15)	1.14 (1.13-1.15)
**Ethnicity**					
White (ref)	665,365 (465,840)	83.5	1.00	1.00	1.00
Black	8,610 (3,115)	42.0	0.34 (0.33-0.35)	0.37 (0.36-0.39)	0.38 (0.37-0.40)
South Asian	18,360 (7,610)	49.2	0.42 (0.41-0.43)	0.46 (0.45-0.47)	0.46 (0.45-0.47)
Mixed	2,455 (1,185)	56.4	0.51 (0.48-0.54)	0.53 (0.50-0.56)	0.54 (0.51-0.57)
Other	3,965 (2,140)	64.7	0.66 (0.64-0.69)	0.69 (0.66-0.72)	0.68 (0.65-0.71)
**IMD**					
1 most deprived (ref)	105,175 (60,455)	70.5	1.00	1.00	1.00
2	127,700 (83,030)	78.5	1.24 (1.23-1.26)	1.19 (1.18-1.21)	1.19 (1.18-1.20)
3	160,310 (112,170)	83.3	1.41 (1.40-1.43)	1.32 (1.31-1.33)	1.31 (1.30-1.33)
4	157,740 (113,965)	85.3	1.51 (1.50-1.53)	1.41 (1.39-1.42)	1.39 (1.38-1.41)
5 least deprived	147,835 (110,270)	87.4	1.64 (1.62-1.65)	1.52 (1.50-1.53)	1.50 (1.48-1.51)
**Setting**					
Urban city or town (ref)	373,270 (258,070)	82.5	1.00	1.00	1.00
Urban conurbation	140,300 (86,560)	74.6	0.90 (0.89-0.91)	0.99 (0.98-1.00)	0.98 (0.97-0.99)
Rural town or village	185,185 (135,255)	86.2	1.10 (1.09-1.10)	1.02 (1.01-1.03)	1.02 (1.01-1.02)
**Kidney disease subgroup**					
CKD3a (ref)	431,830 (312,330)	82.9	1.00	1.00	1.00
CKD3b	199,175 (130,145)	81.4	0.92 (0.92-0.93)	0.94 (0.93-0.94)	0.93 (0.92-0.94)
CKD4–5	51,305 (27,610)	78.7	0.85 (0.84-0.86)	0.88 (0.87-0.89)	0.87 (0.86-0.88)
RRT (dialysis)	3,715 (1,830)	71.1	0.98 (0.93-1.03)	1.07 (1.02-1.12)	1.05 (1.00-1.10)
RRT (Tx)	12,735 (7,970)	67.3	2.05 (1.99-2.12)	2.37 (2.30-2.45)	2.44 (2.36-2.52)
**Primary care coding of kidney disease**					
CKD diagnostic code	439,595 (301,095)	82.2	1.02 (1.01-1.02)	1.03 (1.02-1.03)	1.05 (1.04-1.06)
**Risk group (occupation/access)**					
Care home resident	44,470 (19,620)	82.7	0.86 (0.85-0.87)	0.92 (0.90-0.93)	0.98 (0.96-0.99)
Health/social care worker	1,040 (745)	73.5	1.40 (1.30-1.51)	1.45 (1.35-1.56)	1.40 (1.30-1.51)
Housebound	50,195 (25,025)	77.2	0.72 (0.72-0.73)	0.75 (0.74-0.76)	0.77 (0.76-0.78)
End of life care	36,515 (15,080)	79.1	0.89 (0.87-0.90)	0.92 (0.90-0.93)	0.92 (0.90-0.94)
**Risk group (clinical)**					
Prior SARS-CoV-2	20,685 (9,985)	75.8	0.86 (0.84-0.88)	0.91 (0.89-0.93)	0.92 (0.90-0.94)
Immunosuppression	56,985 (35,705)	74.3	1.20 (1.19-1.22)	1.16 (1.14-1.18)	1.19 (1.18-1.21)
Moderate/severe obesity	53,820 (36,900)	78.9	0.89 (0.88-0.89)	0.93 (0.92-0.94)	0.94 (0.93-0.96)
Diabetes	190,955 (122,995)	79.1	0.88 (0.88-0.89)	0.94 (0.93-0.94)	0.95 (0.94-0.95)
CRD (inc. asthma)	92,340 (58,055)	80.8	0.97 (0.96-0.97)	0.98 (0.97-0.99)	0.98 (0.97-0.99)
Chronic heart disease	347,840 (229,640)	82.4	0.99 (0.98-0.99)	0.97 (0.97-0.98)	0.98 (0.98-0.99)
Chronic liver disease	18,560 (12,195)	78.9	1.00 (0.99-1.02)	1.02 (1.00-1.04)	1.02 (1.00-1.04)
Asplenia	5,960 (4,170)	83.2	1.10 (1.07-1.14)	1.08 (1.05-1.12)	1.08 (1.04-1.11)
Cancer (non-haematologic)	130,945 (88,215)	84.6	1.09 (1.09-1.10)	1.06 (1.05-1.07)	1.07 (1.06-1.07)
Haematologic cancer	20,430 (12,960)	79.2	1.29 (1.27-1.31)	1.13 (1.11-1.16)	1.17 (1.14-1.19)
Organ transplant (non-kidney)	2,190 (1,430)	73.2	1.51 (1.43-1.59)	1.56 (1.48-1.64)	1.99 (1.88-2.10)
CND (inc. learning disability)	135,005 (80,650)	80.4	0.89 (0.89-0.90)	0.90 (0.89-0.91)	0.90 (0.90-0.91)
Severe mental illness	7,075 (3,795)	69.3	0.70 (0.68-0.72)	0.75 (0.72-0.77)	0.77 (0.74-0.79)
CEV (other)	5,850 (4,005)	80.9	1.02 (0.99-1.05)	1.07 (1.04-1.10)	1.03 (1.00-1.07)

Cumulative coverage was determined based on Kaplan-Meier estimates, censoring at death, deregistration, or 31st August 2022. Minimally adjusted models included age, care home residence, and health and social care worker status given their use in vaccine prioritisation criteria. Partially adjusted models additionally included housebound status, receipt of end-of-life care, setting, sex, ethnicity, IMD quintile, prior SARS-CoV-2 infection, immunosuppression, and haematologic cancer. Fully adjusted models included all covariates. CI, confidence interval; CKD, chronic kidney disease; HR, hazard ratio; IMD, index of multiple deprivation; OR, odds ratio; RRT, renal replacement therapy; Tx, transplant.

Supplementary Table 7. Cox proportional hazards models for factors associated with completion of a 4-dose vaccination series in kidney disease subgroups.

	CKD3a		CKD3b		CKD4-5		RRT (dialysis)		RRT (Tx)	
**Variable**	**Cov. (%)**	**HR (95% CI)**	**Cov. (%)**	**HR (95% CI)**	**Cov. (%)**	**HR (95% CI)**	**Cov. (%)**	**HR (95% CI)**	**Cov. (%)**	**HR (95% CI)**
**Age**										
16–64 (ref)	47.4	1.00	51.1	1.00	54.7	1.00	51.8	1.00	61.9	1.00
65–69	55.6	1.19 (1.12-1.27)	61.0	1.23 (1.10-1.38)	62.1	1.26 (1.03-1.53)	61.6	1.27 (0.97-1.67)	80.4	1.63 (1.53-1.75)
70–74	68.4	1.62 (1.54-1.70)	70.0	1.44 (1.31-1.59)	68.1	1.31 (1.11-1.55)	71.8	1.49 (1.13-1.96)	84.3	1.72 (1.60-1.85)
75–79	84.7	2.69 (2.58-2.82)	82.1	2.10 (1.93-2.27)	79.0	1.73 (1.52-1.97)	75.3	1.64 (1.32-2.05)	87.1	1.72 (1.56-1.90)
80+	83.5	2.78 (2.66-2.90)	81.8	2.16 (2.00-2.34)	79.3	1.74 (1.53-1.99)	76.6	1.77 (1.42-2.20)	83.1	1.75 (1.49-2.06)
**Sex**										
Female (ref)	81.7	1.00	79.9	1.00	76.6	1.00	66.6	1.00	69.0	1.00
Male	84.7	1.13 (1.12-1.14)	83.5	1.16 (1.15-1.18)	81.2	1.19 (1.16-1.21)	73.0	1.14 (1.03-1.26)	66.1	0.92 (0.88-0.96)
**Ethnicity**										
White (ref)	84.4	1.00	82.9	1.00	80.8	1.00	75.5	1.00	72.9	1.00
Black	41.8	0.36 (0.35-0.38)	42.5	0.39 (0.36-0.41)	42.3	0.41 (0.37-0.47)	50.1	0.57 (0.44-0.72)	35.1	0.39 (0.34-0.46)
South Asian	51.1	0.46 (0.44-0.47)	48.3	0.45 (0.43-0.47)	44.2	0.44 (0.40-0.48)	49.1	0.54 (0.45-0.65)	43.1	0.51 (0.47-0.56)
Mixed	58.8	0.56 (0.52-0.60)	55.5	0.52 (0.47-0.59)	45.3	0.47 (0.37-0.60)	[R]	[R]	39.1	0.49 (0.36-0.65)
Other	65.2	0.66 (0.63-0.70)	65.0	0.68 (0.63-0.74)	63.9	0.79 (0.67-0.93)	[R]	[R]	54.0	0.63 (0.53-0.75)
**IMD**										
1 most deprived (ref)	71.9	1.00	71.1	1.00	66.7	1.00	54.9	1.00	48.7	1.00
2	79.7	1.20 (1.18-1.21)	78.1	1.17 (1.15-1.20)	75.5	1.19 (1.14-1.25)	68.6	1.37 (1.18-1.60)	61.1	1.35 (1.25-1.46)
3	84.0	1.31 (1.30-1.33)	83.1	1.30 (1.28-1.33)	80.1	1.31 (1.25-1.36)	76.1	1.47 (1.26-1.72)	71.6	1.65 (1.53-1.78)
4	86.1	1.40 (1.38-1.42)	84.7	1.38 (1.35-1.40)	83.2	1.42 (1.36-1.48)	78.8	1.56 (1.33-1.83)	76.6	1.82 (1.69-1.97)
5 least deprived	88.1	1.51 (1.49-1.53)	86.6	1.48 (1.45-1.51)	85.4	1.52 (1.46-1.58)	77.4	1.53 (1.30-1.80)	80.0	1.98 (1.84-2.15)
**Setting**										
Urban city or town (ref)	83.4	1.00	82.1	1.00	79.3	1.00	70.7	1.00	68.2	1.00
Urban conurbation	76.1	0.99 (0.98-1.00)	74.0	0.97 (0.95-0.98)	70.9	1.01 (0.98-1.05)	64.3	1.15 (1.00-1.32)	56.9	1.05 (0.98-1.13)
Rural town or village	86.9	1.02 (1.01-1.03)	85.4	1.01 (1.00-1.02)	83.8	1.04 (1.01-1.07)	80.2	1.09 (0.97-1.23)	78.6	1.13 (1.07-1.20)
**Primary care coding of kidney disease**										
CKD diagnostic code	83.6	1.03 (1.03-1.04)	82.0	1.08 (1.06-1.09)	79.0	1.08 (1.04-1.13)	72.4	1.10 (0.95-1.29)	69.0	1.15 (1.09-1.21)
**Risk group (occupation/access)**										
Care home resident	82.4	0.91 (0.89-0.92)	83.6	0.96 (0.94-0.99)	81.8	1.05 (1.00-1.11)	57.1	0.83 (0.62-1.12)	50.0	0.58 (0.40-0.83)
Health/social care worker	73.2	1.42 (1.28-1.57)	76.1	1.37 (1.11-1.70)	[R]	[R]	[R]	[R]	71.2	1.31 (1.15-1.48)
Housebound	77.8	0.74 (0.73-0.75)	77.6	0.77 (0.75-0.78)	74.2	0.78 (0.75-0.81)	68.3	0.93 (0.77-1.12)	72.6	0.96 (0.87-1.07)
End of life care	80.2	0.91 (0.89-0.93)	79.2	0.93 (0.90-0.96)	74.8	0.91 (0.86-0.96)	63.1	0.77 (0.64-0.94)	73.4	1.23 (1.00-1.51)
**Risk group (clinical)**										
Prior SARS-CoV-2	77.8	0.91 (0.89-0.94)	77.0	0.92 (0.89-0.95)	71.6	0.88 (0.82-0.95)	56.4	0.77 (0.65-0.91)	54.1	0.84 (0.74-0.95)
Immunosuppression	74.1	1.19 (1.17-1.22)	77.3	1.19 (1.15-1.23)	74.6	1.27 (1.19-1.36)	60.6	1.12 (0.91-1.37)	70.6	1.18 (1.13-1.24)
Moderate/severe obesity	79.8	0.93 (0.92-0.94)	79.2	0.94 (0.93-0.96)	75.8	0.94 (0.90-0.98)	62.3	0.90 (0.74-1.09)	64.5	1.00 (0.92-1.08)
Diabetes	79.9	0.93 (0.92-0.94)	79.3	0.96 (0.95-0.97)	76.8	0.97 (0.95-0.99)	68.3	0.96 (0.87-1.06)	67.5	1.05 (1.00-1.11)
CRD (inc. asthma)	81.6	0.98 (0.97-0.99)	80.3	0.98 (0.97-1.00)	78.1	1.00 (0.97-1.04)	72.3	1.04 (0.90-1.20)	68.3	0.95 (0.87-1.03)
Chronic heart disease	83.4	0.97 (0.97-0.98)	81.7	0.98 (0.97-0.99)	79.2	1.00 (0.97-1.02)	73.4	1.02 (0.93-1.13)	72.4	1.06 (1.01-1.11)
Chronic liver disease	79.5	1.03 (1.01-1.05)	79.2	1.03 (1.00-1.07)	76.1	1.03 (0.96-1.11)	66.6	1.19 (0.97-1.47)	69.5	1.19 (1.07-1.32)
Asplenia	84.5	1.09 (1.05-1.14)	83.4	1.10 (1.03-1.17)	75.3	1.01 (0.89-1.14)	[R]	[R]	63.8	0.93 (0.76-1.13)
Cancer (non-haematologic)	85.3	1.07 (1.06-1.08)	83.8	1.06 (1.05-1.08)	82.0	1.08 (1.05-1.11)	78.3	1.09 (0.97-1.22)	78.7	1.16 (1.07-1.26)
Haematologic cancer	79.0	1.17 (1.14-1.21)	80.3	1.17 (1.12-1.22)	77.3	1.13 (1.04-1.23)	72.2	1.38 (1.13-1.69)	73.4	1.00 (0.86-1.18)
Organ transplant (non-kidney)	72.5	2.08 (1.94-2.23)	74.1	1.86 (1.68-2.05)	69.0	1.52 (1.27-1.82)	61.5	1.63 (1.18-2.24)	[N]	[N]
CND (inc. learning disability)	81.2	0.90 (0.89-0.91)	79.9	0.90 (0.88-0.91)	76.9	0.92 (0.89-0.95)	67.5	0.90 (0.79-1.02)	71.4	1.04 (0.97-1.12)
Severe mental illness	68.4	0.74 (0.71-0.77)	71.6	0.78 (0.74-0.83)	71.4	0.87 (0.78-0.97)	35.2	0.58 (0.37-0.92)	54.3	0.87 (0.68-1.12)

, et al. *BMJ Open* 2023; 13:e066164. doi: 10.1136/bmjopen-2022-066164

CEV (other)	81.1	1.06 (1.02-1.11)	80.5	1.11 (1.05-1.18)	77.9	1.06 (0.95-1.18)	[N]	[N]	[N]	[N]

Individuals were included in the analysis if they were ≥75 years of age at baseline, care home residents, transplant recipients, or had a history of haematologic malignancy or immunosuppression. Counts are rounded to the nearest 5. Cumulative coverage was determined based on Kaplan-Meier estimates, censoring at death, deregistration, or 31st August 2022. Hazard ratios and 95% confidence intervals were derived from partially adjusted models that included age, care home residence, health and social care worker status, housebound status, receipt of end-of-life care, setting (urban/rural), sex, ethnicity, IMD quintile, prior SARS-CoV-2 infection, immunosuppression, and haematologic cancer. Data are redacted [R] for any rows in which there were >0 and ≤10 event or non-event counts after rounding to the nearest 5, with non-event counts calculated among uncensored individuals. CEV, clinically extremely vulnerable; CKD, chronic kidney disease; CND, chronic neurological disease; IMD, index of multiple deprivation; [N], covariate absent in all individuals by definition; [R] redacted; RRT, renal replacement therapy; Tx, transplant.

, et al. *BMJ Open* 2023; 13:e066164. doi: 10.1136/bmjopen-2022-066164

Supplementary Table 8. Cumulative 4-dose coverage in subgroups defined by deprivation index, ethnicity, age, and kidney disease severity.

	IMD 1 (most deprived)				IMD 2				IMD 3				IMD 4				IMD 5 (least deprived)			
	White		Minority ethnic groups		White		Minority ethnic groups		White		Minority ethnic groups		White		Minority ethnic groups		White		Minority ethnic groups	
**Characteristic**	N	%	N	%	N	%	N	%	N	%	N	%	N	%	N	%	N	%	N	%
**All**	94295	74.8	10880	35.3	119075	80.8	8620	47.8	153940	84.4	6370	56.5	153430	86.0	4310	62.8	144625	87.8	3205	70.8
**Age**																				
16–64	2805	47.0	1190	24.6	2860	58.5	815	32.0	3105	62.4	505	40.1	2830	66.6	340	42.7	2360	71.8	235	49.9
65–69	1030	54.1	235	29.1	1130	60.0	205	38.1	1340	64.9	130	43.1	1275	69.5	100	47.4	1120	72.9	65	66.7
70–74	1635	61.0	190	36.2	2000	70.2	165	39.2	2400	73.8	135	44.1	2270	74.0	100	60.9	2040	77.3	70	63.1
75–79	25380	76.4	2475	37.5	31565	82.5	2175	52.0	40160	86.2	1690	57.3	39405	88.0	1140	65.3	36295	89.7	920	73.3
80+	63440	76.2	6790	36.5	81520	81.6	5260	49.2	106935	84.9	3915	59.0	107650	86.2	2635	64.5	102815	87.8	1920	72.1
**Kidney disease subgroup**																				
CKD3a	55615	75.7	5845	36.3	72130	81.7	4670	49.1	95515	85.0	3600	57.3	97010	86.7	2460	63.6	93065	88.4	1920	70.7
CKD3b	28670	74.9	2890	35.2	35050	80.3	2330	46.5	44240	84.1	1665	57.9	42920	85.3	1130	62.8	39485	86.9	790	72.7
CKD4-5	7685	72.0	1105	30.8	9260	77.8	805	50.0	11155	81.6	580	51.0	10635	84.0	345	60.3	9500	85.9	235	67.7
RRT (dial.)	530	60.6	235	40.3	640	73.3	165	46.5	695	79.7	110	52.7	650	80.4	70	64.1	565	78.4	60	55.6
RRT (Tx)	1800	56.7	805	30.4	2000	67.8	650	39.7	2330	75.2	415	49.8	2215	79.5	305	54.2	2015	81.4	200	63.3

Cumulative coverage (%) was determined based on Kaplan-Meier estimates, censoring at death, deregistration, or 31st August 2022. Minority ethnic groups are combined due to their low combined prevalence within the study population. CKD, chronic kidney disease; dial., dialysis; IMD, index of multiple deprivation quintile; RRT, renal replacement therapy; Tx, transplant.

Key

	<50%
	50–75%

, *et al. BMJ Open* 2023; 13:e066164. doi: 10.1136/bmjopen-2022-066164
