# Supplementary material for: Factors associated with COVID-19 vaccine uptake in people with kidney disease: an OpenSAFELY cohort study
Source: BMJ Open. 2023 Jan 31;13(1):e066164. doi: 10.1136/bmjopen-2022-066164 (PMC9890277; doi:10.1136/bmjopen-2022-066164)
Supplement: Author's manuscript [file bmjopen-2022-066164.draft_revisions.pdf]

BMJ Open is committed to open peer review. As part of this commitment we make the peer review history of every article we publish publicly available.

When an article is published we post the peer reviewers' comments and the authors' responses online. We also post the versions of the paper that were used during peer review. These are the versions that the peer review comments apply to.

The versions of the paper that follow are the versions that were submitted during the peer review process. They are not the versions of record or the final published versions. They should not be cited or distributed as the published version of this manuscript.

BMJ Open is an open access journal and the full, final, typeset and author-corrected version of record of the manuscript is available on our site with no access controls, subscription charges or pay-per-view fees (http://bmjopen.bmj.com).

If you have any questions on BMJ Open's open peer review process please email info.bmjopen@bmj.com

BMJ Open

Factors associated with COVID-19 vaccine uptake in people with kidney disease: an OpenSAFELY cohort study

Journal:	*BMJ Open*
Manuscript ID	bmjopen-2022-066164
Article Type:	Original research
Date Submitted by the Author:	06-Jul-2022

Complete List of Authors:	Parker, Edward PK; London School of Hygiene & Tropical Medicine<br>Tazare, John; London School of Hygiene & Tropical Medicine<br>Hulme, William J; Oxford University<br>Bates, Christopher; TPP<br>Carr, Edward J; The Francis Crick Institute<br>Cockburn, Jonathan; TPP<br>Curtis, Helen; Oxford University<br>Fisher, Louis; Oxford University<br>Green, Amelia CA; Oxford University<br>Harper, Sam; TPP<br>Hester, Frank; TPP<br>Horne, Elsie MF; University of Bristol<br>Loud, Fiona; Kidney Care UK<br>Lyon, Susan; UK Kidney Association<br>Mahalingasivam, Viyaasan; London School of Hygiene & Tropical Medicine<br>Mehrkar, Amir; Oxford University<br>Nab, Linda; Oxford University<br>Parry, John; TPP<br>Santhakumaran, Shalini; UK Renal Registry<br>Steenkamp, Retha; UK Renal Registry<br>Sterne, Jonathan; University of Bristol<br>Walker, Alex; Oxford University<br>Williamson, Elizabeth; London School of Hygiene & Tropical Medicine<br>Willicombe, Michelle; Imperial College London<br>Zheng, Bang; London School of Hygiene & Tropical Medicine<br>Goldacre, Ben; Oxford University<br>Nitsch, Dorothea; London School of Hygiene & Tropical Medicine<br>Tomlinson, Laurie; London School of Hygiene & Tropical Medicine

Keywords:	COVID-19, Kidney & urinary tract disorders < UROLOGY, Public health < INFECTIOUS DISEASES

SCHOLARONE™
Manuscripts

I, the Submitting Author has the right to grant and does grant on behalf of all authors of the Work (as defined in the below author licence), an exclusive licence and/or a non-exclusive licence for contributions from authors who are: i) UK Crown employees; ii) where BMJ has agreed a CC-BY licence shall apply, and/or iii) in accordance with the terms applicable for US Federal Government officers or employees acting as part of their official duties; on a worldwide, perpetual, irrevocable, royalty-free basis to BMJ Publishing Group Ltd ("BMJ") its licensees and where the relevant Journal is co-owned by BMJ to the co-owners of the Journal, to publish the Work in this journal and any other BMJ products and to exploit all rights, as set out in our licence.

The Submitting Author accepts and understands that any supply made under these terms is made by BMJ to the Submitting Author unless you are acting as an employee on behalf of your employer or a postgraduate student of an affiliated institution which is paying any applicable article publishing charge ("APC") for Open Access articles. Where the Submitting Author wishes to make the Work available on an Open Access basis (and intends to pay the relevant APC), the terms of reuse of such Open Access shall be governed by a Creative Commons licence – details of these licences and which Creative Commons licence will apply to this Work are set out in our licence referred to above.

Other than as permitted in any relevant BMJ Author's Self Archiving Policies, I confirm this Work has not been accepted for publication elsewhere, is not being considered for publication elsewhere and does not duplicate material already published. I confirm all authors consent to publication of this Work and authorise the granting of this licence.

For peer review only

Factors associated with COVID-19 vaccine uptake in people with kidney disease: an OpenSAFELY cohort study

The OpenSAFELY Collaborative: Edward PK Parker[1], John Tazare[1], William J Hulme[2], Christopher Bates[3], Edward J Carr[4], Jonathan Cockburn[3], Helen J Curtis[2], Louis Fisher[2], Amelia CA Green[2], Sam Harper[3], Frank Hester[3], Elsie MF Horne[5,6], Fiona Loud[7], Susan Lyon[8], Viyaasan Mahalingasivam[1], Amir Mehrkar[2], Linda Nab[2], John Parry[3], Shalini Santhakumaran[9], Retha Steenkamp[9], Jonathan AC Sterne[5,6,10], Alex J Walker[2], Elizabeth J Williamson[1], Michelle Willicombe[11,12], Bang Zheng[1], Ben Goldacre[2], Dorothea Nitsch[1,9], Laurie A Tomlinson[1]

[1] London School of Hygiene and Tropical Medicine, Keppel Street, London, WC1E 7HT, UK
[2] Bennett Institute for Applied Data Science, Nuffield Department of Primary Care Health Sciences, University of Oxford, OX2 6GG, UK
[3] TPP, TPP House, 129 Low Lane, Horsforth, Leeds, LS18 5PX, UK
[4] The Francis Crick Institute, London, NW1 1AT, UK
[5] Population Health Sciences, University of Bristol, Oakfield House, Oakfield Grove, Bristol, BS8 2BN, UK
[6] NIHR Bristol Biomedical Research Centre, Bristol, UK
[7] Kidney Care UK, Alton, UK
[8] Patient Council, UK Kidney Association, Bristol, UK
[9] UK Renal Registry, Bristol, UK
[10] Health Data Research UK South-West, Bristol, UK
[11] Centre for Inflammatory Disease, Department of Immunology and Inflammation, Imperial College London, Hammersmith Campus, Du Cane Road London, W12 0NN, UK
[12] Imperial College Renal and Transplant Centre, Imperial College Healthcare NHS Trust, Hammersmith Hospital, London, W12 0HS, UK

Correspondence to: Laurie Tomlinson, London School of Hygiene and Tropical Medicine, Keppel Street, London, WC1E 7HT, UK; Email: laurie.tomlinson@lshtm.ac.uk

For peer review only - http://bmjopen.bmj.com/site/about/guidelines.xhtml

Abstract

Objective: To characterise factors associated with COVID-19 vaccine uptake among people with kidney disease in England.

Design: Retrospective cohort study using the OpenSAFELY-TPP platform, performed with the approval of NHS England.

Setting: Individual-level routine clinical data from 24 million people across GPs in England using TPP software. Primary care data were linked directly with COVID-19 vaccine records up to 11th May 2022 and with renal replacement therapy (RRT) status via the UK Renal Registry.

Participants: A cohort of adults with stage 3–5 chronic kidney disease (CKD) or receiving RRT at the start of the COVID-19 vaccine roll-out were identified based on evidence of reduced estimated glomerular filtration rate (eGFR) or inclusion in the UK Renal Registry.

Main outcome measures: Dose-specific vaccine coverage over time was determined from 1st December 2020 to 11th May 2022. Individual-level factors associated with receipt of a 3- or 4-dose vaccine series were explored via Cox proportional hazards models.

Results: 948,845 people with stage 3–5 CKD or receiving RRT were included. Cumulative vaccine coverage as of 11th May 2022 was 97.5%, 97.0%, and 93.5% for doses 1, 2, and 3, respectively, and 61.1% among individuals with one or more indications for receipt of a fourth dose. Delayed 3-dose vaccine uptake was associated with non-White ethnicity, social deprivation, and severe mental illness – associations that were consistent across CKD severity subgroups, dialysis patients, and kidney transplant recipients. Similar associations were observed for 4-dose uptake, which was also delayed among care home residents.

Conclusion: Although high primary vaccine and booster dose coverage has been achieved among people with kidney disease in England, key disparities in vaccine uptake remain across clinical and demographic groups and 4-dose coverage is suboptimal. Identifying how to address these disparities is a priority to reduce risk of severe disease in this vulnerable patient group.

Key words: COVID-19; chronic kidney disease; NHS England; SARS-CoV-2; vaccination; vaccine coverage.

For peer review only - http://bmjopen.bmj.com/site/about/guidelines.xhtml

What is already known on this topic

People with kidney disease are known to be at higher risk of severe COVID-19 and post-vaccination infection than the general population, and have been prioritised for extended primary series and booster doses in many countries.

Throughout the COVID-19 vaccine roll-out in England, vaccine uptake in the general population has been lower in non-White ethnic groups and in areas of higher social deprivation. These factors have also been linked with higher vaccine hesitancy among haemodialysis and kidney transplant recipients. However, their impact on vaccine coverage among people with kidney disease remains unknown.

What this study adds

Among 948,845 people in England who had stage 3–5 chronic kidney disease or were receiving renal replacement therapy at the start of the COVID-19 vaccine roll-out, cumulative coverage with at least 3 doses was 93.5% as of 11th May 2022, while coverage with at least 4 doses (among those eligible) was 61.1%.

Vaccine uptake was slower in non-White ethnic groups, in areas of higher social deprivation, and in people with a history of severe mental illness – findings that were highly consistent across people with kidney disease of varying severity, including dialysis and transplant recipients.

These findings demonstrate that high-risk clinical risk groups do not escape the entrenched inequities shaping vaccine acceptance and uptake.

For peer review only

Background

Since the emergence of SARS-CoV-2, individuals with kidney disease – in particular those requiring renal replacement therapy (RRT) – have been at particular risk of enduring the most harmful effects of COVID-19[1 2]. While vaccination has the potential to mitigate this risk, people with kidney disease exhibit impaired COVID-19 vaccine immunogenicity and effectiveness compared to healthy adults[3 4]. Among individuals who have received a 2-dose primary vaccine series, chronic kidney disease (CKD) and receipt of RRT have been identified as key risk factors for subsequent infection, COVID-19-related hospitalisation, and COVID-19-related mortality[5].

Given their continued risk of COVID-19 after primary vaccination, people with kidney disease have been prioritised for additional vaccine doses in many countries. In the UK, the Joint Committee on Vaccination and Immunisation (JCVI) guidelines recommended that individuals with substantial immunosuppression (including primary or acquired immunodeficiency and recent or ongoing immunosuppressive therapy[6]) receive a third primary vaccine dose from 1st September 2021. A subset of individuals with kidney disease, such as those receiving immunosuppressive therapy following kidney transplant, would have been eligible for this third primary dose. Subsequently, all individuals with CKD were included in the initial JCVI priority groups to be offered a booster dose from 14th September 2021[7], with eligibility later extending to all adults as of 29th November 2021[8].

Within the UK's guidelines there are two routes to being offered a fourth dose of COVID-19 vaccine. Firstly, severely immunosuppressed individuals who received a third primary dose would become eligible for a booster (fourth) dose at an interval of 3 months, and a second booster (fifth) dose at least 3 months later. Secondly, since February 2022[9], individuals who are >75 years of age, care home residents, or immunosuppressed become eligible for a fourth dose as a 2-dose primary course followed by autumn 2021 and spring 2022 boosters. As CKD incidence climbs with age, the majority of people with kidney disease in the UK would now be eligible for a fourth dose via this second route.

Global and national variation in COVID-19 vaccine uptake is monitored via several online dashboards[10-12]. In England, relative vaccine uptake among demographic and clinical subgroups, including people with CKD, is reported in weekly coverage reports using the OpenSAFELY platform[12 13]. However, in these summary reports it remains unclear whether COVID-19 vaccine coverage varies according to kidney disease severity or in relation to other demographic or clinical factors in this high-risk group.

To address these issues, we conducted a retrospective cohort study within the OpenSAFELY-TPP database (https://opensafely.org). We report on the status of COVID-19 vaccination among people with kidney disease up to 11th May 2022 and highlight individual-level characteristics associated with vaccine uptake.

Methods

Data sources

All data were linked, stored, and analysed securely within the OpenSAFELY platform https://opensafely.org/. OpenSAFELY is a data analytics platform created by our team on behalf of NHS England to address urgent COVID-19 research questions. The dataset analysed within OpenSAFELY-TPP is based on 24 million people currently registered with GP surgeries using TPP SystmOne software. Data include pseudonymised data such as coded diagnoses, medications, and physiological parameters. No free text data are included. All code is shared openly for review and re-use under MIT open license (https://github.com/opensafely/ckd-coverage-ve). Detailed pseudonymised patient data is potentially re-identifiable and therefore not shared. Primary care data are linked through OpenSAFELY with other pseudonymised datasets, including COVID-19 testing

For peer review only - http://bmjopen.bmj.com/site/about/guidelines.xhtml

records via the Second Generation Surveillance System (SGSS), A&E attendance and hospital records via NHS Digital's Hospital Episode Statistics (HES), national death registry records from the Office for National Statistics (ONS), and RRT status via the UK Renal Registry (UKRR). Vaccination status is directly available within GP records via the National Immunisation Management System (NIMS).

Study population

Baseline clinical and demographic characteristics were defined as of 1st December 2020 – the month in which the UK COVID-19 vaccination programme commenced – with the exception of age, which was calculated as of 31st March 2021 as per UK Health Security Agency recommendations[14]. We assessed eligibility for inclusion in the study cohort among individuals who were at least 16 years of age and had been registered in OpenSAFELY-TPP for at least 3 months at baseline. We excluded individuals if their sex, ethnicity, geographical region, or index of multiple deprivation (IMD) were unknown, or if they had an atypical vaccine record (more than five doses recorded by 11th May 2022 or any two doses recorded within a period of 14 days).

We determined kidney function based on the most recent serum creatinine measurement in the 2 years preceding 1st December 2020 (where available) and converted this into estimated glomerular filtration rate (eGFR) using the CKD epidemiology collaboration (CKD-EPI) equation without specification of ethnicity[15]. Individuals receiving RRT were identified based on their treatment modality (dialysis or transplant) within the UKRR as of 31st December 2020 (the UKRR status update closest to the baseline date).

We included individuals who were receiving RRT as well as people with CKD based on evidence of reduced kidney function (eGFR <60 ml/min/1.73 m^2) in the absence of RRT. Individuals with primary care codes suggesting prior receipt of dialysis or kidney transplant but absent from the UKRR cohort were excluded. To assess CKD severity, we distinguished between stage 3a (eGFR of 45–59 ml/min/1.73 m^2), stage 3b (eGFR of 30–44 ml/min/1.73 m^2), and stage 4–5 CKD (eGFR <30 ml/min/1.73 m^2).

Outcomes

We report on cumulative coverage for doses 1, 2, 3, 4, and 5 (via any combination of vaccine products) up to 11th May 2022. For the analysis of individual-level factors associated with vaccine uptake, our primary outcome was receipt of a 3-dose series of any vaccine product. For the purposes of this study, we do not distinguish between third primary and booster doses, or between 100 µg (full) or 50 µg (half) doses of the vaccine product mRNA-1273 (with the latter recommended for booster doses).

Receipt of a 4-dose series of any vaccine product was considered as a secondary outcome. Notably, not all members of our cohort would be eligible for a fourth dose based on current UK guidelines (described above). We therefore limited our secondary outcome analysis to a subset of the population with at least one indicator for receipt of a fourth dose (3 primary + 1 booster or 2 primary + 2 boosters), including individuals who were ≥75 years of age at baseline, care home residents, transplant recipients, or had a history of haematologic malignancy or immunosuppression.

Covariates

Covariates of interest in relation to demography, vaccine prioritisation, and population accessibility included: age (five categories defined by JCVI priority group cut-offs); sex; ethnicity (White, Black or Black British, Asian or Asian British, Mixed, or other); social deprivation based on IMD quintile (derived from an individual's postcode at Lower Super Output Area); setting (urban, urban conurbation, or rural); care home residence; health and social care worker status; housebound status; and receipt of end-of-life care.

Clinical covariates of interest included kidney disease subgroup (CKD3a, CKD3b, CKD4–5, RRT [dialysis], and RRT [transplant]) and presence or absence of pre-existing conditions including: prior

For peer review only - http://bmjopen.bmj.com/site/about/guidelines.xhtml

SARS-CoV-2 infection; immunosuppression; moderate/severe obesity (body mass index ≥30 kg/m^2 based on the most recent measurement in the 5 years before baseline); diabetes; chronic respiratory disease (including asthma); chronic heart disease; chronic liver disease; asplenia; non-haematologic cancer; haematologic cancer; non-kidney organ transplant; chronic neurological disease (including learning disability); severe mental illness; and classification as clinically extremely vulnerable[16] in the absence of any of the conditions listed above (including RRT). To assess the potential influence of primary care coding relating to kidney disease, we included the presence or absence of diagnostic codes associated with stage 3–5 CKD at baseline.

Statistical analysis

We used Kaplan-Meier estimates to determine cumulative dose-specific vaccine coverage over time, censoring at death, deregistration, or the end of the study period (11th May 2022). Individuals remained in the at-risk population for receiving doses 2–5 regardless of their vaccination status for prior doses. To identify demographic and clinical covariates associated with time to receipt of a 3-dose (primary outcome) or 4-dose (secondary outcome) vaccine series, we used Cox proportional hazards models, stratified by region, and censoring at death, deregistration, or the end of the study period. For each covariate, we determined hazard ratios (HRs) and 95% confidence intervals (CIs) with sequential adjustment for baseline covariates. Minimally adjusted models included age, care home residence, and health and social care worker status given their use in JCVI prioritisation criteria. Partially adjusted models extended these models to include additional variables potentially linked with vaccine access or uptake, including housebound status, receipt of end-of-life care, setting, sex, ethnicity, IMD quintile, prior SARS-CoV-2 infection, immunosuppression, and haematologic cancer. Fully adjusted models included all covariates. Schoenfeld residual plots were used to assess validity of the proportional hazards assumption. As a precaution against small-number disclosures, we rounded counts to the nearest 5, redacted non-zero rounded counts or non-counts of ≤10, and delayed Kaplan-Meier steps until ≥10 events had occurred.

To determine whether HRs differed according to kidney disease severity, partially adjusted models were explored in the following subgroups: CKD3a, CKD3b, CKD4–5, RRT (dialysis), and RRT (transplant). To assess whether conclusions were robust to Cox model assumptions and representative of factors associated with vaccine coverage at the time of the analysis cut-off (11th May 2022), we performed a sensitivity analysis for the primary outcome in which we used partially adjusted logistic regression models (using the same variables above but with region as an additional covariate) among individuals who remained alive and registered throughout follow-up.

Patient and public involvement

Patients were not directly involved in developing this study. We maintain a publicly available website (https://opensafely.org) through which members of the public or patient groups are invited to contact us regarding this study or the broader OpenSAFELY project.

Results

Vaccine coverage and product profile by dose

A total of 948,845 individuals met the criteria for inclusion in the cohort (**Supplementary Figure 1**). Of these, 66.1% had CKD3a, 24.9% had CKD3b, 6.7% had CKD4–5, 1.0% were receiving RRT via dialysis, and 1.3% were receiving RRT via kidney transplant. Clinical and demographic characteristics of the overall cohort and kidney disease subgroups are summarised in **Table 1**. In comparison to people with CKD, RRT recipients were markedly younger (with associated declines in several age-associated comorbidities) and were more likely to be male and non-White.

For peer review only - http://bmjopen.bmj.com/site/about/guidelines.xhtml

Table 1. Baseline characteristics of study population.

Characteristic	All N = 948,845	CKD3a N = 627,265	CKD3b N = 236,205	CKD4–5 N = 63,165	RRT (dialysis) N = 9,440	RRT (transplant) N = 12,770
**Age**						
16–64	104,770 (11.0%)	71,970 (11.5%)	13,245 (5.6%)	5,870 (9.3%)	4,390 (46.5%)	9,295 (72.8%)
65–69	70,790 (7.5%)	54,435 (8.7%)	10,815 (4.6%)	3,020 (4.8%)	1,095 (11.6%)	1,425 (11.2%)
70–74	135,900 (14.3%)	102,975 (16.4%)	24,730 (10.5%)	5,865 (9.3%)	1,145 (12.1%)	1,180 (9.2%)
75–79	172,530 (18.2%)	124,030 (19.8%)	38,245 (16.2%)	8,410 (13.3%)	1,220 (12.9%)	620 (4.9%)
80+	464,860 (49.0%)	273,855 (43.7%)	149,170 (63.2%)	40,000 (63.3%)	1,590 (16.8%)	250 (2.0%)
**Sex**						
Female	530,685 (55.9%)	355,460 (56.7%)	134,120 (56.8%)	32,700 (51.8%)	3,480 (36.9%)	4,925 (38.6%)
Male	418,160 (44.1%)	271,805 (43.3%)	102,085 (43.2%)	30,465 (48.2%)	5,960 (63.1%)	7,845 (61.4%)
**Ethnicity**						
White	888,875 (93.7%)	590,315 (94.1%)	222,900 (94.4%)	57,975 (91.8%)	7,295 (77.3%)	10,390 (81.4%)
Mixed	5,110 (0.5%)	3,415 (0.5%)	1,065 (0.5%)	350 (0.6%)	140 (1.5%)	140 (1.1%)
Asian or Asian British	32,245 (3.4%)	18,720 (3.0%)	7,595 (3.2%)	3,120 (4.9%)	1,295 (13.7%)	1,510 (11.8%)
Black or Black British	16,345 (1.7%)	10,840 (1.7%)	3,240 (1.4%)	1,225 (1.9%)	570 (6.0%)	470 (3.7%)
Other ethnic groups	6,270 (0.7%)	3,975 (0.6%)	1,405 (0.6%)	495 (0.8%)	135 (1.4%)	260 (2.0%)
**Index of multiple deprivation quintile**						
1 most deprived	158,960 (16.8%)	100,965 (16.1%)	40,770 (17.3%)	12,015 (19.0%)	2,610 (27.6%)	2,600 (20.4%)
2	180,015 (19.0%)	116,850 (18.6%)	45,630 (19.3%)	12,685 (20.1%)	2,185 (23.1%)	2,665 (20.9%)
3	214,280 (22.6%)	141,655 (22.6%)	53,770 (22.8%)	14,205 (22.5%)	1,890 (20.0%)	2,760 (21.6%)
4	207,080 (21.8%)	139,220 (22.2%)	50,790 (21.5%)	13,015 (20.6%)	1,530 (16.2%)	2,520 (19.7%)
5 least deprived	188,510 (19.9%)	128,575 (20.5%)	45,240 (19.2%)	11,245 (17.8%)	1,230 (13.0%)	2,225 (17.4%)
**Setting**						
Urban city or town	506,660 (53.4%)	334,925 (53.4%)	126,760 (53.7%)	33,625 (53.2%)	4,735 (50.2%)	6,615 (51.8%)
Urban conurbation	199,215 (21.0%)	128,630 (20.5%)	49,545 (21.0%)	14,540 (23.0%)	3,000 (31.8%)	3,495 (27.4%)
Rural	242,975 (25.6%)	163,705 (26.1%)	59,900 (25.4%)	15,000 (23.7%)	1,705 (18.1%)	2,660 (20.8%)
**Primary care coding of kidney disease**						
CKD3–5 diagnostic code	563,335 (59.4%)	306,820 (48.9%)	183,225 (77.6%)	55,645 (88.1%)	7,990 (84.6%)	9,655 (75.6%)
Dialysis code	15,740 (1.7%)	[N]	[N]	[N]	7,435 (78.8%)	8,305 (65.0%)
Kidney transplant code	13,650 (1.4%)	[N]	[N]	[N]	1,290 (13.7%)	12,360 (96.8%)
**Risk group (occupation/access)**						
Care home resident	43,385 (4.6%)	23,700 (3.8%)	14,845 (6.3%)	4,605 (7.3%)	165 (1.7%)	65 (0.5%)
Health/social care worker	5,175 (0.5%)	3,975 (0.6%)	585 (0.2%)	195 (0.3%)	65 (0.7%)	355 (2.8%)
Housebound	53,520 (5.6%)	26,735 (4.3%)	18,770 (7.9%)	6,800 (10.8%)	600 (6.4%)	615 (4.8%)
End of life care	39,430 (4.2%)	20,070 (3.2%)	13,185 (5.6%)	5,385 (8.5%)	600 (6.4%)	180 (1.4%)
**Risk group (clinical)**						
Prior SARS-CoV-2 *	25,490 (2.7%)	14,290 (2.3%)	7,135 (3.0%)	2,545 (4.0%)	1,000 (10.6%)	515 (4.0%)
Immunosuppression	80,025 (8.4%)	43,575 (6.9%)	18,245 (7.7%)	5,525 (8.7%)	2,065 (21.9%)	10,615 (83.1%)
Moderate/severe obesity	108,950 (11.5%)	71,070 (11.3%)	27,390 (11.6%)	8,000 (12.7%)	1,325 (14.0%)	1,160 (9.1%)
Diabetes	269,895 (28.4%)	152,745 (24.4%)	81,390 (34.5%)	27,680 (43.8%)	4,040 (42.8%)	4,040 (31.6%)

For peer review only - http://bmjopen.bmj.com/site/about/guidelines.xhtml

Chronic respiratory disease (inc. asthma)	210,095 (22.1%)	136,395 (21.7%)	54,735 (23.2%)	14,675 (23.2%)	2,035 (21.6%)	2,255 (17.7%)
Chronic heart disease	425,575 (44.9%)	255,835 (40.8%)	125,250 (53.0%)	35,895 (56.8%)	4,740 (50.2%)	3,860 (30.2%)
Chronic liver disease	31,285 (3.3%)	20,250 (3.2%)	7,650 (3.2%)	2,245 (3.6%)	550 (5.8%)	585 (4.6%)
Asplenia	8,405 (0.9%)	5,485 (0.9%)	2,035 (0.9%)	575 (0.9%)	140 (1.5%)	170 (1.3%)
Cancer (non-haematologic)	164,110 (17.3%)	103,955 (16.6%)	45,090 (19.1%)	12,800 (20.3%)	1,305 (13.8%)	960 (7.5%)
Haematologic cancer	19,990 (2.1%)	11,885 (1.9%)	5,705 (2.4%)	1,845 (2.9%)	310 (3.3%)	240 (1.9%)
Organ transplant (non-kidney) †	2,170 (0.2%)	1,195 (0.2%)	685 (0.3%)	215 (0.3%)	75 (0.8%)	[N]
CND (inc. learning disability)	158,200 (16.7%)	96,415 (15.4%)	46,255 (19.6%)	12,905 (20.4%)	1,440 (15.3%)	1,185 (9.3%)
Severe mental illness	16,270 (1.7%)	10,465 (1.7%)	4,105 (1.7%)	1,310 (2.1%)	220 (2.3%)	170 (1.3%)
Clinically extremely vulnerable (other) §	5,750 (0.6%)	3,480 (0.6%)	1,465 (0.6%)	810 (1.3%)	[N]	[N]
**Region**						
London	30,045 (3.2%)	18,640 (3.0%)	7,200 (3.0%)	2,530 (4.0%)	765 (8.1%)	910 (7.1%)
East of England	212,465 (22.4%)	141,680 (22.6%)	52,210 (22.1%)	13,535 (21.4%)	2,070 (21.9%)	2,970 (23.3%)
East Midlands	180,590 (19.0%)	120,660 (19.2%)	44,370 (18.8%)	11,645 (18.4%)	1,660 (17.6%)	2,250 (17.6%)
North East	43,350 (4.6%)	28,385 (4.5%)	10,990 (4.7%)	2,845 (4.5%)	535 (5.7%)	595 (4.7%)
North West	87,040 (9.2%)	57,085 (9.1%)	22,155 (9.4%)	5,950 (9.4%)	685 (7.3%)	1,165 (9.1%)
South East	56,310 (5.9%)	37,160 (5.9%)	14,080 (6.0%)	3,755 (5.9%)	580 (6.1%)	735 (5.8%)
South West	166,805 (17.6%)	111,260 (17.7%)	41,645 (17.6%)	10,905 (17.3%)	1,205 (12.8%)	1,790 (14.0%)
West Midlands	36,265 (3.8%)	23,340 (3.7%)	9,225 (3.9%)	2,605 (4.1%)	630 (6.7%)	465 (3.6%)
Yorkshire and the Humber	135,975 (14.3%)	89,055 (14.2%)	34,335 (14.5%)	9,385 (14.9%)	1,310 (13.9%)	1,885 (14.8%)

Data are n (%) after rounding to the nearest 5. Primary care codes and risk groups are coded by separate binary variables; percentages under these table subheadings therefore do not sum to 100. Among people with CKD4–5, 57,890 had stage 4 CKD (eGFR 15–29 ml/min/1.73 m^2) and 5,275 had stage 5 CKD (eGFR <15ml/min/1.73 m^2). CKD, chronic kidney disease; CND, chronic neurological disease; [N], covariate absent in all individuals by definition; RRT, renal replacement therapy. * Based on prior evidence of a positive SARS-CoV-2 test, COVID-19-related primary care code, or COVID-19-related hospitalisation as of 1st December 2020; † Excludes individuals with kidney transplants based on primary care coding or UK Renal Registry status; § Classified as clinically extremely vulnerable in the absence of any of the comorbidities listed above (including RRT).

For peer review only

For peer review only - http://bmjopen.bmj.com/site/about/guidelines.xhtml

Cumulative coverage in people with kidney disease as of 11th May 2022 was 97.5% for dose 1, 97.0% for dose 2, and 93.5% for dose 3 (**Figure 1**). Dose 4 coverage was 45.7% for the cohort as a whole and 61.1% among the subset of individuals with at least one indicator for receipt of a third primary or second booster dose. Only 3,045 individuals had received a fifth dose, corresponding to a cumulative coverage of 0.4% in the population as a whole.

For their primary vaccine schedule (doses 1 and 2), most individuals received a homologous series of BNT162b2 (53.4% of recorded schedules) or ChAdOx1-S (45.7%), with <1% receiving either heterologous schedules or mRNA-1273 (**Supplementary Table 1**). For subsequent doses, individuals primarily received BNT162b2 (92.1% and 47.0% of recorded doses 3 and 4, respectively) or mRNA-1273 (7.6% and 52.9% of recorded doses 3 and 4, respectively), reflecting JCVI recommendations that RNA vaccines be preferentially used for third primary or booster doses. Accordingly, heterologous third doses were prevalent, primarily involving the administration of BNT162b2 or mRNA-1273 to individuals who had received two prior doses of ChAdOx1-S (39.7% and 5.1% of recorded 3-dose schedules, respectively), though homologous vaccination with BNT162b2 remained the most common 3-dose schedule overall (52.0%; **Supplementary Table 1**).

Factors associated with 3-dose vaccine uptake

Associations between individual-level factors and time to receipt of a 3-dose series are shown in **Figure 2** and **Supplementary Table 2**. HRs were generally stable across minimally, partially, and fully adjusted models (**Supplementary Table 2**). To mitigate potential overadjustment bias, we report on partially adjusted models hereafter.

Vaccine uptake was faster in older individuals and in health and social care workers, reflecting the inclusion of these groups in JCVI prioritisation criteria. By contrast, 3-dose uptake was slower among individuals living in more deprived areas (HR [95% CI] of 1.43 [1.42–1.44] for comparison of least deprived with most deprived IMD quintile), among non-White ethnic groups (HRs of 0.48–0.69 for comparisons of non-White with White individuals), and individuals who were housebound (HR 0.61 [0.61–0.62]) or receiving end-of-life care (HR 0.87 [0.85–0.88]). These associations were highly consistent across kidney disease subgroups, including RRT recipients (**Figure 3** and **Supplementary Table 3**).

In comparison to people with CKD3a, 3-dose vaccine uptake was somewhat slower among people with CKD3b (HR 0.94 [0.93–0.94]) and CKD4–5 (0.87 [0.87–0.88]), but faster among individuals receiving RRT (HRs of 1.09 [1.07–1.12] and 1.45 [1.42–1.49] for dialysis and transplant recipients, respectively). Having a primary care code associated with stage 3–5 CKD was not strongly associated with vaccine uptake in the cohort as a whole (HR 1.04 [1.03–1.04]), but was associated with faster vaccine uptake in more severe kidney disease subgroups (HRs of <1.1 for CKD3a and CKD3b, 1.17 [1.13–1.20] for CKD4–5, 1.14 [1.07–1.22] for dialysis recipients, and 1.10 [1.05–1.15] for transplant recipients; **Figure 3**).

Other clinical covariates tended to have modest associations with vaccine uptake (HRs of 0.9–1.1). However, immunosuppression (HR 1.19 [1.18–1.20]) and receipt of non-kidney organ transplants (HR 1.31 [1.26–1.38]) were associated with faster 3-dose uptake, while severe mental illness was associated with slower uptake (HR 0.79 [0.78–0.81]). These associations were highly consistent across CKD and RRT subgroups, albeit less pronounced in kidney transplant recipients (**Figure 3**).

Schoenfeld residual plots revealed no major violations of the proportional hazards assumption. Associations were generally consistent when explored via logistic regression among 821,670 individuals who were uncensored throughout follow-up (**Supplementary Table 2**), with the exception that having a clinical code associated with stage 3–5 CKD was positively correlated with vaccine uptake in the overall cohort using this approach (odds ratio 1.23 [1.21–1.25]).

Factors associated with 4-dose vaccine uptake

For peer review only - http://bmjopen.bmj.com/site/about/guidelines.xhtml

A total of 679,405 individuals (71.6% of the overall cohort) had at least one indicator for receipt of a fourth dose (3 primary + 1 booster or 2 primary + 2 boosters) and were included in the secondary outcome analysis. Baseline clinical and demographic characteristics for these individuals are summarised in **Supplementary Table 4**.

Factors associated with 4-dose uptake were generally consistent with those observed for the primary outcome analyses. Vaccine uptake was faster in older individuals and in health and social care workers, but slower in individuals living in more deprived areas (HR of 1.54 [1.53–1.56] for comparison of least deprived with most deprived IMD quintile), in non-White ethnic groups (HRs of 0.35–0.71 for comparisons of non-White with White individuals), and in individuals who were housebound (HR 0.60 [0.59–0.61]) or receiving end-of-life care (HR 0.88 [0.86–0.89]; **Figure 4** and **Supplementary Table 5**). Four-dose uptake was also slower among care home residents (HR 0.73 [0.72–0.74]), contrary to the findings observed for 3-dose uptake.

Clinical factors associated with eligibility for a third primary dose were also associated with faster 4-dose uptake, including immunosuppression (HR 1.26 [1.24–1.28]), haematologic cancer (HR 1.23 [1.20–1.26]) and transplant (HRs for 2.96 [2.86–3.06] and 1.82 [1.72–1.93] for kidney and non-kidney transplants, respectively). On the other hand, severe mental illness (HR 0.72 [0.69–0.74]) and chronic neurological disease including learning disability (HR 0.87 [0.86–0.88]) were associated with slower 4-dose uptake. Associations for demographic and clinical covariates were highly consistent across kidney disease subgroups, including RRT recipients (**Supplementary Figure 2** and **Supplementary Table 6**). Having a primary care code associated with stage 3–5 CKD was not associated with 4-dose uptake (HRs of 0.98–1.07 for the overall cohort, CKD subgroups, and dialysis recipients), but was associated with faster uptake in kidney transplant recipients (HR 1.13 [1.07–1.19]).

Discussion

Summary

The principal aim of the COVID-19 vaccine roll-out is to protect those at greatest risk of severe disease. Based on the data presented here, considerable progress has been made towards achieving this goal among people with kidney disease in England, with 97.0% of individuals having received at least 2 doses of COVID-19 vaccine, 93.5% having received 3 doses, and 61.1% of eligible individuals having received 4 doses as of 11th May 2022.

Although high coverage has been achieved across people with kidney disease, considerable variation in vaccine uptake was evident in relation to the clinical and demographic characteristics of these individuals. Non-White ethnicity and social deprivation were negatively associated with vaccine uptake, even in high-risk groups such as dialysis and kidney transplant recipients. Among clinical covariates, uptake was higher in individuals with immunosuppression, but lower in individuals with a history of severe mental illness. These patterns were consistent across stage 3–5 CKD and RRT subgroups, and continue to impact 4-dose uptake during the ongoing spring booster campaign.

Findings in context

Inequities in COVID-19 vaccine uptake according to ethnicity and social deprivation have previously been reported for the general population[13], with similar disparities also impacting past national vaccination campaigns in the UK[17]. While our study is among the first to explore variation in COVID-19 vaccine uptake in people with kidney disease, several surveys of haemodialysis patients and kidney transplant recipients in France[18] and the USA[19-21] have reported higher COVID-19 vaccine hesitancy among people with non-White ethnicity and those living in more deprived areas – findings consistent with the delayed vaccine uptake in these groups reported here.

While prior studies have offered inconsistent findings with respect to the link between mental illness and COVID-19 vaccine uptake[13 22], these typically focused on 1-dose coverage in the early months of

For peer review only - http://bmjopen.bmj.com/site/about/guidelines.xhtml

the vaccine roll-out, whereas the current study extends the window of follow-up throughout the primary and booster campaigns and focuses on a high-risk clinical group, limiting comparability.

Strengths and limitations

By harnessing the OpenSAFELY-TPP database, we incorporated linked health records across a population of 948,845 people with kidney disease, providing a comprehensive and timely view of the clinical and demographics factors associated with COVID-19 vaccine uptake in this high-risk population. We used eGFR levels to define CKD, thereby enabling us to avoid potential bias or imprecision associated with the inconsistent use of diagnostic codes across practices and regions, though the use and reporting of creatinine measurements may also be subject to regional inconsistency. The use of UKRR data also enabled us to audit vaccine uptake among high-risk groups while reducing potential misclassification of current RRT status. Finally, we used complementary statistical modelling strategies to ensure our conclusions were robust to the assumptions of any single approach.

Nonetheless, several limitations of our study should be considered. First, the OpenSAFELY-TPP database does not offer a geographically representative sample of England, with regions such as London (3% of the present cohort) notably under-represented. Thus, region-specific variation in vaccine uptake may not be captured. Second, by considering completion of a 3-dose series as the primary endpoint, our Cox models may contravene the assumption of non-informative censoring, with the receipt of vaccine doses 1 and 2 resulting in a reduced likelihood of COVID-19-related mortality (and associated censoring). However, a sensitivity analysis restricted to uncensored individuals offered a consistent view of the factors associated with vaccine uptake at the end of follow-up. Finally, pre-existing conditions were considered as either present or absent, thereby failing to capture potentially informative details regarding the timing and severity of these conditions. This may have impacted our ability to accurately determine individuals eligible for a fourth COVID-19 vaccine dose, given that factors such as the timing of immunosuppressive therapy relative to vaccination are included in JCVI eligibility criteria.

Policy implications and interpretation

Our findings suggest that high-risk clinical risk groups do not escape the entrenched inequities shaping vaccine acceptance and uptake. Targeted activities to promote vaccine uptake among under-immunised communities have increasingly been used to expand the reach of the COVID-19 vaccine roll-out[23 24], and may be crucial towards enhancing booster dose coverage in people with kidney disease over the coming months.

The prevalence of stage 3–5 CKD diagnostic codes varied from 49% in people with CKD3a to 88% in people with CKD4–5. These primary care codes may act as a proxy for GP, NHS, or personal awareness of CKD, which is often asymptomatic in its early stages. Indeed, coding status has previously been linked with improved primary care management of CKD, including delivery of influenza and pneumococcal vaccines[25]. Although we did not observe a strong link between CKD3–5 code and vaccine uptake in people with CKD3a or CKD3b, the presence of a diagnostic code was associated with earlier 3-dose uptake in people with CKD4–5 and those receiving RRT. This finding highlights the importance of consistent diagnostic code usage by GPs to promote timely vaccination of high-risk individuals.

Given the reduced immunogenicity of COVID-19 vaccines in people with kidney disease alongside the lower effectiveness of vaccines against the Omicron variant and subvariants [4 26], 3 doses may be insufficient to provide long-term protection in this high-risk population. A gradual increase in dose 4 coverage was apparent in early 2022, with a notable uptick following the launch of the spring booster campaign in February 2022[13]. Although not all individuals in the present cohort would be eligible for a 4-dose series, 71% had at least one indicator for receipt of a fourth dose. Cumulative dose-4 coverage in these individuals was 61.1%, suggesting that concerted efforts to promote booster dose uptake among people with kidney disease remains essential. Evolving evidence regarding the risk of infection after primary vaccination among people with kidney disease serves as a clear mandate for

For peer review only - http://bmjopen.bmj.com/site/about/guidelines.xhtml

the necessity of booster doses in these patients[5]. Vaccine-hesitant individuals report personal concerns over vaccine safety; therefore, clear communication about existing and ongoing research into the safety profile of primary and booster COVID-19 vaccine doses in individuals with kidney disease may help promote vaccine uptake in this population.

Conclusion

This study offers a comprehensive view of the status of the COVID-19 vaccine roll-out in a large cohort of people with kidney disease. To our knowledge, this is the first study to explore the link between individual-level characteristics and COVID-19 vaccine uptake at scale in this high-risk clinical group. Vaccine uptake was significantly slower among people with non-White ethnicity, in areas of higher social deprivation, and in people with severe mental illness. Addressing these disparities during ongoing and future booster campaigns will be essential to protect people with kidney disease from the most harmful sequelae of COVID-19.

Acknowledgements

We are very grateful for all the support received from the TPP Technical Operations team throughout this work, and for generous assistance from the information governance and database teams at NHS England/NHS Transformation Directorate.

Contributors

Conceptualisation: EPKP, EJC, FL, DN, and LAT; Methodology: EPKP, JT, WJH, LF, ACAG, VM, SS, EJW, BG, DN, and LAT; Software: WJH, CB, JC, SH, FH, JP, and AJW; Formal analysis, EPKP and JT; Data curation: CB, JC, SH, FH, AM, JP, SS, RS, AJW, and DN; Writing–original draft, EPKP; Writing–review & editing, EJC, HJC, EMFH, FL, SL, LN, SS, MW, BZ, DN, and LAT; Visualisation, EPKP; Supervision, DN and LAT; Project administration: EPKP and AM; Funding acquisition, EPKP, BG, DN, and LAT.

Funding

This work was jointly funded by the Wellcome Trust (222097/Z/20/Z); MRC (MR/V015757/1, MC_PC-20059, MR/W016729/1); NIHR (NIHR135559, COV-LT2-0073), and Health Data Research UK (HDRUK2021.000, 2021.0157). EPKP received funding from the UKRI COVID-19 Longitudinal Health and Wellbeing National Core Study (Phase 1 LHW-NCS, MC_PC-20059) through a secondment scheme. The views expressed are those of the authors and not necessarily those of the NIHR, NHS England, UK Health Security Agency or the Department of Health and Social Care. Funders had no role in the study design, collection, analysis, and interpretation of data; in the writing of the report; and in the decision to submit the article for publication.

Competing interests

BG's work on better use of data in healthcare more broadly is currently funded in part by: the Bennett Foundation, the Wellcome Trust, NIHR Oxford Biomedical Research Centre, NIHR Applied Research Collaboration Oxford and Thames Valley, the Mohn-Westlake Foundation; all Bennett Institute staff are supported by BG's grants on this work. BG is a Non-Executive Director at NHS Digital. EJW holds grants from MRC

Ethical approval

This study was approved by the Health Research Authority (REC reference 20/LO/0651) and by the London School of Hygiene & Tropical Medicine's Ethics Board (reference 21863

Information governance

NHS England is the data controller for OpenSAFELY-TPP; TPP is the data processor; all study authors using OpenSAFELY have the approval of NHS England. This implementation of OpenSAFELY is hosted within the TPP environment which is accredited to the ISO 27001 information security standard and is NHS IG Toolkit compliant. Patient data has been pseudonymised for analysis and linkage using industry standard cryptographic hashing techniques; all pseudonymised datasets transmitted for linkage onto OpenSAFELY are encrypted; access to the platform is via a

For peer review only - http://bmjopen.bmj.com/site/about/guidelines.xhtml

virtual private network (VPN) connection, restricted to a small group of researchers; the researchers hold contracts with NHS England and only access the platform to initiate database queries and statistical models; all database activity is logged; only aggregate statistical outputs leave the platform environment following best practice for anonymisation of results such as statistical disclosure control for low cell counts.

The OpenSAFELY research platform adheres to the obligations of the UK General Data Protection Regulation (GDPR) and the Data Protection Act 2018. In March 2020, the Secretary of State for Health and Social Care used powers under the UK Health Service (Control of Patient Information) Regulations 2002 (COPI) to require organisations to process confidential patient information for the purposes of protecting public health, providing healthcare services to the public and monitoring and managing the COVID-19 outbreak and incidents of exposure; this sets aside the requirement for patient consent.

Taken together, these provide the legal bases to link patient datasets on the OpenSAFELY platform. GP practices, from which the primary care data are obtained, are required to share relevant health information to support the public health response to the pandemic, and have been informed of the OpenSAFELY analytics platform.

This project includes data from the UKRR derived from patient-level information collected by the NHS as part of the care and support of kidney patients. We thank all kidney patients and kidney centres involved. The data are collated, maintained, and quality assured by the UKRR, which is part of the UK Kidney Association. Access to the data was facilitated by the UKRR's Data Release Group. UKRR data are used within OpenSAFELY to address a limited number of critical audit and service delivery questions related to the impact of COVID-19 on patients with kidney disease.

Data sharing

Detailed pseudonymised patient data is potentially re-identifiable and therefore not shared. Access to the underlying identifiable and potentially re-identifiable pseudonymised electronic health record data is tightly governed by various legislative and regulatory frameworks, and restricted by best practice. The data in OpenSAFELY is drawn from General Practice data across England where TPP is the data processor. TPP developers initiate an automated process to create pseudonymised records in the core OpenSAFELY database, which are copies of key structured data tables in the identifiable records. These pseudonymised records are linked onto key external data resources that have also been pseudonymised via SHA-512 one-way hashing of NHS numbers using a shared salt. Bennett Institute for Applied Data Science developers and PIs holding contracts with NHS England have access to the OpenSAFELY pseudonymised data tables as needed to develop the OpenSAFELY tools. These tools in turn enable researchers with OpenSAFELY data access agreements to write and execute code for data management and data analysis without direct access to the underlying raw pseudonymised patient data, and to review the outputs of this code. All code for the full data management pipeline – from raw data to completed results for this analysis – and for the OpenSAFELY platform as a whole is available for review at github.com/OpenSAFELY.

Code availability

Data management was performed using Python, with analysis carried out using R 4.0.2. Code for data management and analysis, as well as codelists, are archived online (https://github.com/opensafely/ckd-coverage-ve).

Figure legends

Figure 1. Dose-specific coverage and product profile among people with kidney disease. (A) Cumulative dose-specific vaccine coverage over time. One minus Kaplan-Meier estimates are plotted

For peer review only - http://bmjopen.bmj.com/site/about/guidelines.xhtml

for the receipt of any combination of vaccine products, censoring at death, deregistration, or 11th May 2022. For dose 4, we report overall coverage (solid line) and coverage among the subset of the cohort with at least one indicator for receipt of a fourth dose (3 primary + 1 booster or 2 primary + 2 boosters; dotted line). (B) Vaccine product distribution by dose. Fifth doses were excluded given the small number (N = 3,045) recorded in the cohort.

Figure 2. Factors associated with completion of a 3-dose vaccine series in people with kidney disease. The left-hand panels display hazard ratios and 95% confidence intervals from partially adjusted models that included age, care home residence, health and social care worker status, housebound status, receipt of end-of-life care, setting (urban/rural), sex, ethnicity, IMD quintile, prior SARS-CoV-2 infection, immunosuppression, and haematologic cancer. Confidence intervals are generally too narrow to be visible. See **Supplementary Table 2** for minimally and fully adjusted model outputs. The right-hand panels display the cumulative coverage based on Kaplan-Meier estimates, censoring on death, deregistration, or 11th May 2022. CKD, chronic kidney disease; IMD, index of multiple deprivation; RRT, renal replacement therapy.

Figure 3. Factors associated with completion of a 3-dose vaccine series in kidney disease subgroups. Hazard ratios and 95% confidence intervals were derived from partially adjusted models that included age, care home residence, health and social care worker status, housebound status, receipt of end-of-life care, setting (urban/rural), sex, ethnicity, IMD quintile, prior SARS-CoV-2 infection, immunosuppression, and haematologic cancer. Data are redacted [R] for any rows in which there were >0 and ≤10 event or non-event counts after rounding to the nearest 5, with non-event counts calculated among uncensored individuals. CKD, chronic kidney disease; IMD, index of multiple deprivation; [N], covariate absent in all individuals by definition; [R], redacted; RRT, renal replacement therapy.

Figure 4. Factors associated with completion of a 4-dose vaccine series in people with kidney disease. The left-hand panels display hazard ratios and 95% confidence intervals from partially adjusted models that included age, care home residence, health and social care worker status, housebound status, receipt of end-of-life care, setting (urban/rural), sex, ethnicity, IMD quintile, prior SARS-CoV-2 infection, immunosuppression, and haematologic cancer. Confidence intervals are generally too narrow to be visible. See Supplementary Table 4 for minimally and fully adjusted model outputs. The right-hand panels display the cumulative coverage based on Kaplan-Meier estimates, censoring on death, deregistration, or 11th May 2022. CKD, chronic kidney disease; IMD, index of multiple deprivation; RRT, renal replacement therapy.

For peer review only - http://bmjopen.bmj.com/site/about/guidelines.xhtml

References

1. Williamson EJ, Walker AJ, Bhaskaran K, et al. Factors associated with COVID-19-related death using OpenSAFELY. *Nature* 2020;584(7821):430-36.
2. Ng JH, Hirsch JS, Wanchoo R, et al. Outcomes of patients with end-stage kidney disease hospitalized with COVID-19. *Kidney Int* 2020;98(6):1530-39.
3. Chen JJ, Lee TH, Tian YC, et al. Immunogenicity rates after SARS-CoV-2 vaccination in people with end-stage kidney disease: a systematic review and meta-analysis. *JAMA Netw Open* 2021;4(10):e2131749.
4. Carr EJ, Wu M, Harvey R, et al. Neutralising antibodies after COVID-19 vaccination in UK haemodialysis patients. *Lancet* 2021;398(10305):1038-41.
5. The OpenSAFELY Collaborative: Green A, Curtis H, et al. Describing the population experiencing COVID-19 vaccine breakthrough following second vaccination in England: A cohort study from OpenSAFELY. *medRxiv* 2021:2021.11.08.21265380.
6. JCVI. Joint Committee on Vaccination and Immunisation (JCVI) advice on third primary dose vaccination. Available from: https://www.gov.uk/government/publications/third-primary-covid-19-vaccine-dose-for-people-who-are-immunosuppressed-jcvi-advice/joint-committee-on-vaccination-and-immunisation-jcvi-advice-on-third-primary-dose-vaccination (accessed 12 April 2022).
7. JCVI. JCVI statement regarding a COVID-19 booster vaccine programme for winter 2021 to 2022. Available from: https://www.gov.uk/government/publications/jcvi-statement-september-2021-covid-19-booster-vaccine-programme-for-winter-2021-to-2022/jcvi-statement-regarding-a-covid-19-booster-vaccine-programme-for-winter-2021-to-2022 (accessed 12 April 2022).
8. JCVI. JCVI advice on COVID-19 booster vaccines for those aged 18 to 39 and a second dose for ages 12 to 15. Available from: https://www.gov.uk/government/news/jcvi-advice-on-covid-19-booster-vaccines-for-those-aged-18-to-39-and-a-second-dose-for-ages-12-to-15 (accessed 31 May 2022).
9. JCVI. JCVI advises a spring COVID-19 vaccine dose for the most vulnerable. Available from: https://www.gov.uk/government/news/jcvi-advises-a-spring-covid-19-vaccine-dose-for-the-most-vulnerable (accessed 14 April 2022).
10. Mathieu E, Ritchie H, Ortiz-Ospina E, et al. A global database of COVID-19 vaccinations. *Nat Hum Behav* 2021;5(7):947-53.
11. WHO. WHO Coronavirus (COVID-19) Dashboard. Available from: https://covid19.who.int (accessed 12 April 2022).
12. OpenSAFELY. NHS COVID-19 vaccine coverage: weekly report. Available from: https://opensafely.org/research/2021/covid-vaccine-coverage (accessed 12 April 2021).
13. Curtis HJ, Inglesby P, Morton CE, et al. Trends and clinical characteristics of COVID-19 vaccine recipients: a federated analysis of 57.9 million patients' primary care records in situ using OpenSAFELY. *Br J Gen Pract* 2022;72(714):e51-e62.
14. PRIMIS, University of Nottingham. SARS-CoV2 (COVID-19) vaccination uptake reporting specification (version 1). Available from: https://www.nottingham.ac.uk/primis/covid-19/covid-19.aspx (accessed 14 October 2021).
15. Levey AS, Stevens LA, Schmid CH, et al. A new equation to estimate glomerular filtration rate. *Ann Intern Med* 2009;150(9):604-12.
16. UK Health Security Agency. COVID-19: the green book, chapter 14a. Available from: https://www.gov.uk/government/publications/covid-19-the-green-book-chapter-14a (accessed 14 June 2022).
17. Roberts D, Matthews V, Saliba V, et al. National Immunisation Programme: health equity audit. Available from: https://assets.publishing.service.gov.uk/government/uploads/system/uploads/attachment_data/file/957670/immnstn-equity_AUDIT_v11.pdf (accessed 14 April 2022).
18. Andrian T, Koppe L, Novel E, et al. COVID-19 vaccine acceptance among haemodialysis patients: a French survey. *Clin Kidney J* 2021;14(8):1985-86.
19. Garcia P, Montez-Rath ME, Moore H, et al. SARS-CoV-2 vaccine acceptability in patients on hemodialysis: a nationwide survey. *J Am Soc Nephrol* 2021 doi: 10.1681/ASN.2021010104.

For peer review only - http://bmjopen.bmj.com/site/about/guidelines.xhtml

20. Tsapepas D, Husain SA, King KL, et al. Perspectives on COVID-19 vaccination among kidney and pancreas transplant recipients living in New York City. *Am J Health Syst Pharm* 2021;78(22):2040-45.
21. Tummalapalli SL, Cukor D, Bohmart A, et al. A mobile health-based survey to assess COVID-19 vaccine intent and uptake among patients on dialysis. *Kidney Int Rep* 2022;7(3):633-37.
22. Hassan L, Sawyer C, Peek N, et al. COVID-19 vaccination uptake in people with severe mental illness: a UK-based cohort study. *World Psychiatry* 2022;21(1):153-54.
23. England N. Bridging the Uptake Gap – COVID-19 vaccination toolkit for Black African and Black African Caribbean communities. Available from: https://www.england.nhs.uk/coronavirus/publication/bridging-the-uptake-gap-covid-19-vaccination-toolkit-for-black-african-and-black-african-caribbean-communities/ (accessed 14 April 2022).
24. Griffin S. Covid-19: Ethnicity vaccination gap narrows in England, but concerns remain. *BMJ* 2021;372:n505.
25. Kim LG, Cleary F, Wheeler DC, et al. How do primary care doctors in England and Wales code and manage people with chronic kidney disease? Results from the National Chronic Kidney Disease Audit. *Nephrol Dial Transplant* 2018;33(8):1373-79.
26. Andrews N, Stowe J, Kirsebom F, et al. Covid-19 Vaccine effectiveness against the Omicron (B.1.1.529) variant. *N Engl J Med* 2022;386(16):1532-46.

For peer review only

For peer review only - http://bmjopen.bmj.com/site/about/guidelines.xhtml

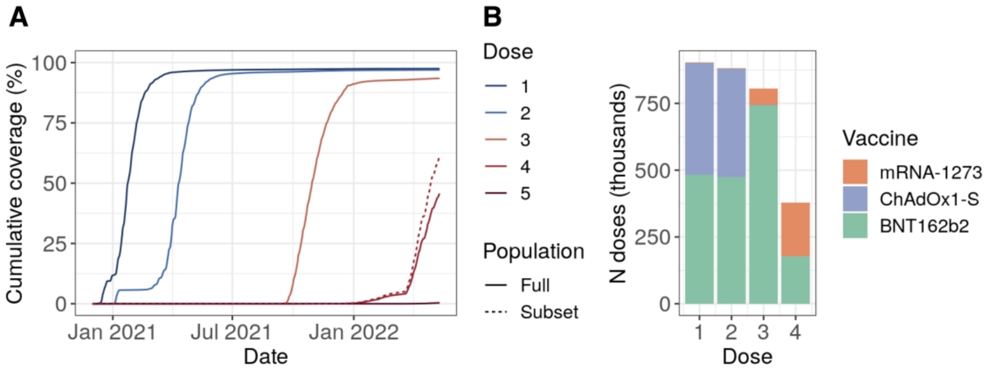

Figure 1

3156x1213mm (72 x 72 DPI)

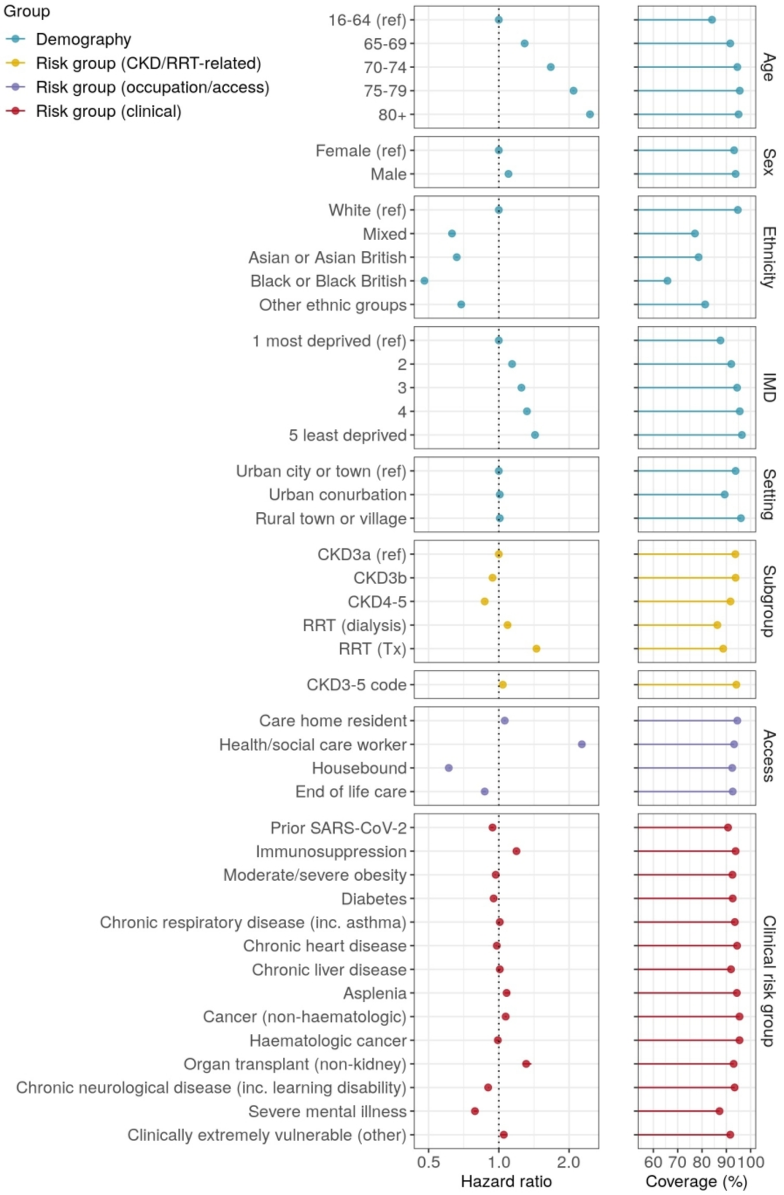

Figure 2

203x304mm (192 x 192 DPI)

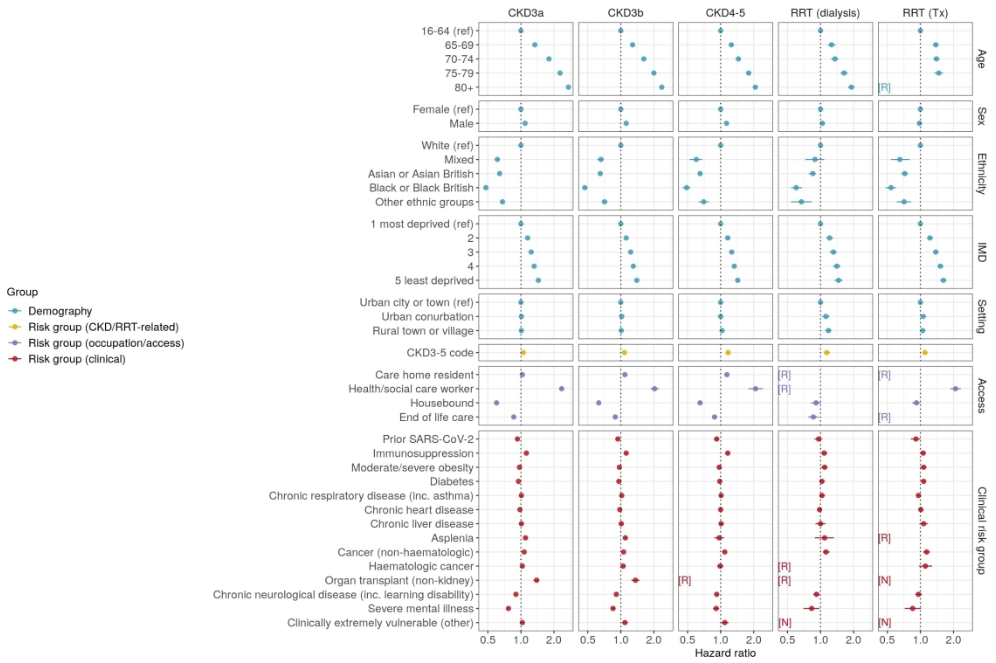

Figure 3

381x254mm (192 x 192 DPI)

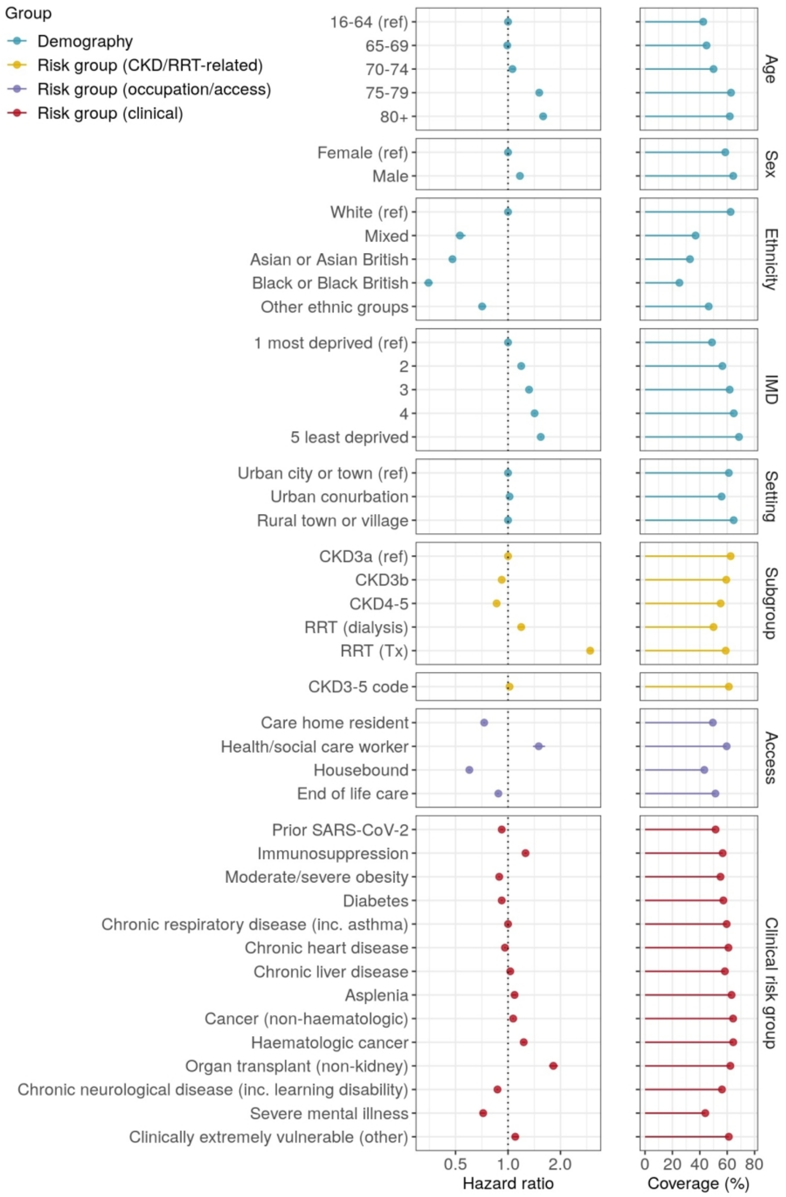

Figure 4

203x304mm (192 x 192 DPI)

Supplementary Appendix

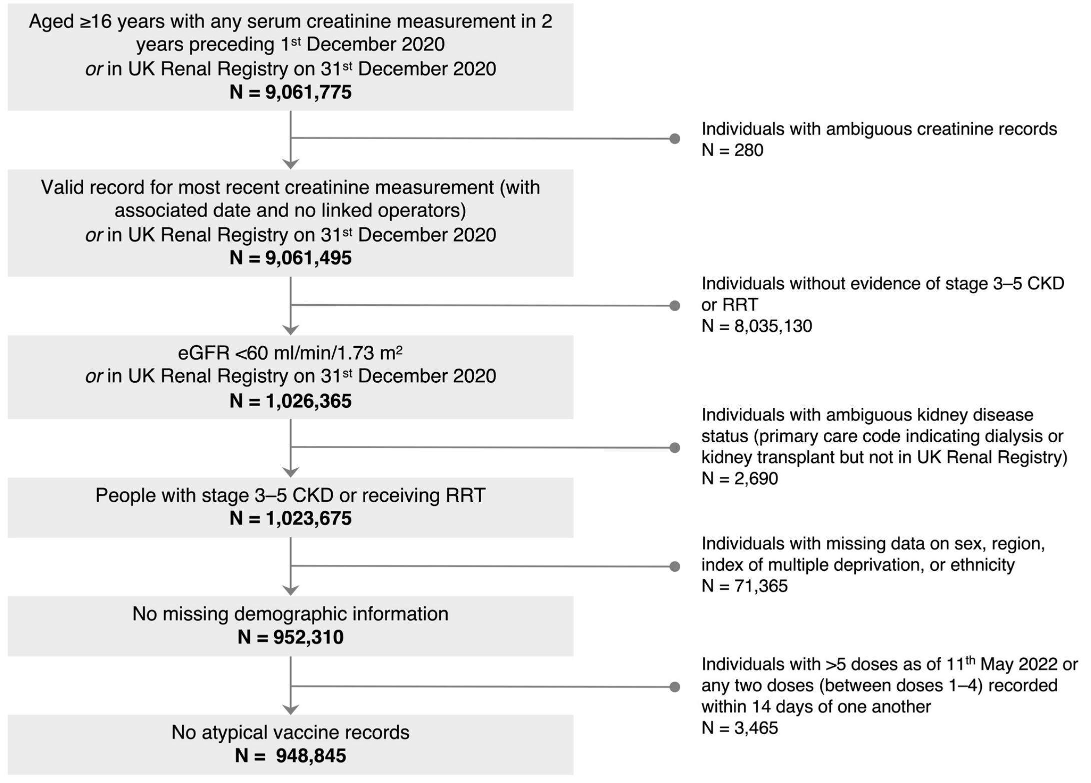

Supplementary Figure 1. Flow chart of study eligibility. Frequencies are rounded to the nearest 5. CKD, chronic kidney disease; eGFR, estimated glomerular filtrate rate; RRT, renal replacement therapy.

review only

For peer review only - http://bmjopen.bmj.com/site/about/guidelines.xhtml

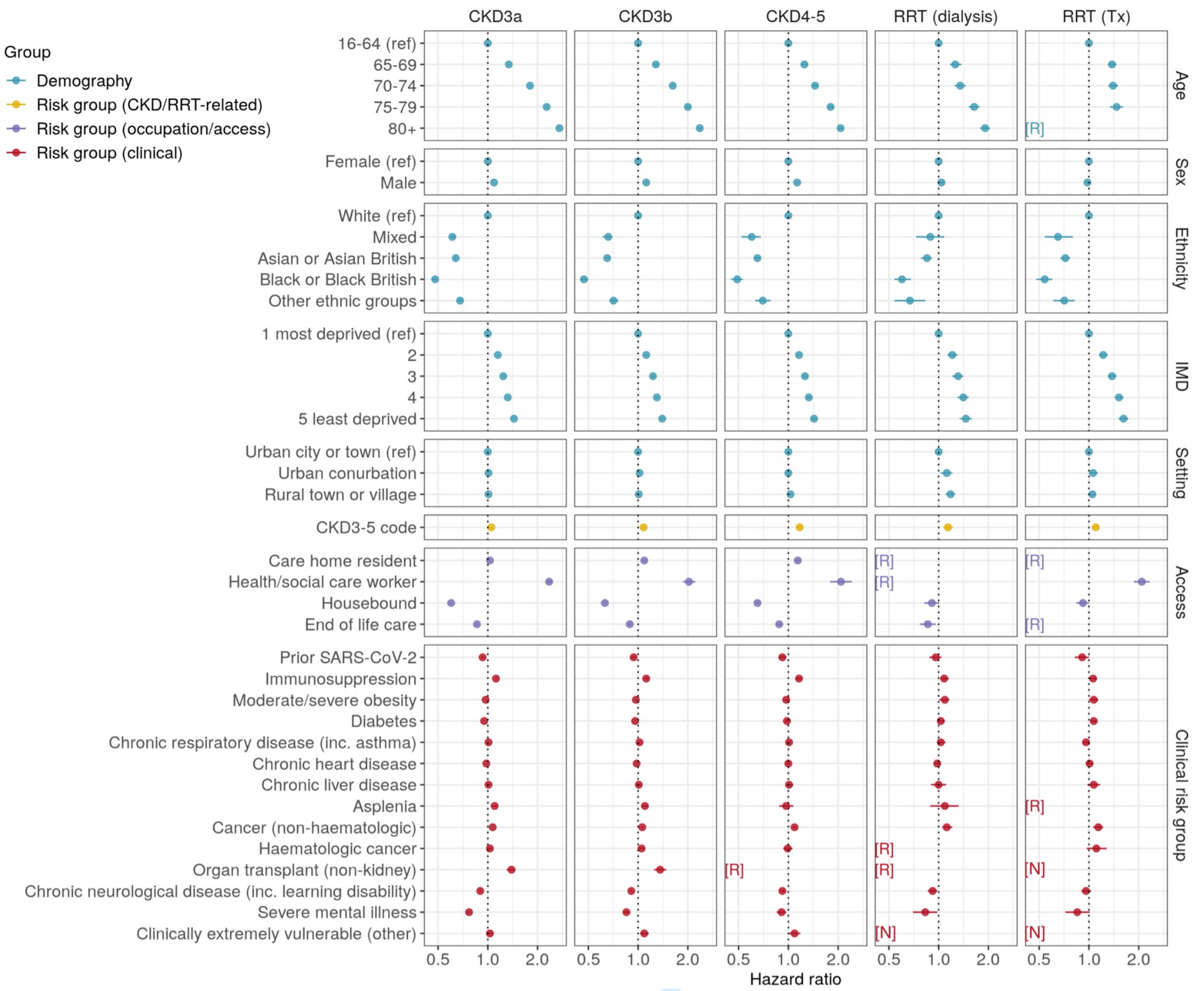

Supplementary Figure 2. Factors associated with completion of a 4-dose vaccine series in kidney disease subgroups. Individuals were included in the analysis if they were ≥75 years of age at baseline, care home residents, transplant recipients, or had a history of haematologic malignancy or immunosuppression. Hazard ratios and 95% confidence intervals were derived from partially adjusted models that included age, care home residence, health and social care worker status, housebound status, receipt of end-of-life care, setting (urban/rural), sex, ethnicity, IMD quintile, prior SARS-CoV-2 infection, immunosuppression, and haematologic cancer. Data are redacted [R] for any rows in which there were >0 and ≤10 event or non-event counts after rounding to the nearest 5, with non-event counts calculated among uncensored individuals. CKD, chronic kidney disease; IMD, index of multiple deprivation; [N], covariate absent in all individuals by definition; [R], redacted; RRT, renal replacement therapy.

only

Supplementary Table 1. Product profile by dose.

Subgroup	Dose 1	Dose 2	Dose 3	Dose 4
**Product**				
BNT	483,280 (53.5%)	473,820 (53.8%)	742,985 (92.1%)	178,065 (47.0%)
AZ	418,795 (46.4%)	405,975 (46.1%)	1,910 (0.2%)	110 (0.0%)
MOD	570 (0.1%)	905 (0.1%)	61,515 (7.6%)	200,445 (52.9%)
**2-dose product combination**				
BNT-BNT	–	470,655 (53.4%)	–	–
AZ-AZ	–	402,395 (45.7%)	–	–
BNT-AZ	–	3,580 (0.4%)	–	–
AZ-BNT	–	3,125 (0.4%)	–	–
Other	–	945 (0.1%)	–	–
**3-dose product combination**				
BNT-BNT-BNT	–	–	419,020 (52.0%)	–
AZ-AZ-BNT	–	–	320,100 (39.7%)	–
AZ-AZ-MOD	–	–	40,875 (5.1%)	–
BNT-BNT-MOD	–	–	20,015 (2.5%)	–
BNT-AZ-BNT	–	–	2,490 (0.3%)	–
AZ-AZ-AZ	–	–	1,670 (0.2%)	–
AZ-BNT-BNT	–	–	1,185 (0.1%)	–
Other	–	–	1,060 (0.1%)	–

Frequencies are rounded to the nearest 5. Any dose combinations given to <1000 individuals were pooled as 'Other'. AZ, ChAdOx1-S (AstraZeneca); BNT, BNT162b2 (Pfizer-BioNTech); MOD, mRNA-1273 (Moderna).

For peer review only

Supplementary Table 2. Cox proportional hazards model and logistic regression outputs for factors associated with completion of a 3-dose vaccination series in people with kidney disease.

	Cox models					Logistic regression	
**Variable**	**N (n events)**	**Cov. (%)**	**HR (95% CI), minimally adjusted**	**HR (95% CI), partially adjusted**	**HR (95% CI), fully adjusted**	**N (n events)**	**OR (95% CI), partially adjusted**
***†Age**							
16–64 (ref)	104,770 (84,815)	84.1	1.00	1.00	1.00	99,200 (83,700)	1.00
65–69	70,790 (62,075)	91.6	1.32 (1.31–1.33)	1.29 (1.27–1.30)	1.31 (1.30–1.33)	66,485 (61,115)	1.90 (1.83–1.96)
70–74	135,900 (122,380)	94.5	1.75 (1.73–1.77)	1.67 (1.66–1.69)	1.71 (1.70–1.73)	126,665 (120,080)	2.69 (2.61–2.78)
75–79	172,530 (155,085)	95.5	2.18 (2.16–2.20)	2.09 (2.07–2.11)	2.15 (2.13–2.17)	157,535 (150,935)	3.30 (3.20–3.40)
80+	464,860 (381,885)	95.0	2.45 (2.43–2.47)	2.46 (2.44–2.48)	2.55 (2.53–2.57)	371,785 (355,415)	3.20 (3.12–3.28)
**†Sex**							
Female (ref)	530,685 (451,975)	93.2	1.00	1.00	1.00	462,955 (433,405)	1.00
Male	418,160 (354,265)	93.8	1.11 (1.11–1.12)	1.10 (1.09–1.10)	1.10 (1.10–1.11)	358,715 (337,840)	1.18 (1.16–1.20)
**†Ethnicity**							
White (ref)	888,875 (764,800)	94.7	1.00	1.00	1.00	768,730 (731,110)	1.00
Mixed	5,110 (3,670)	77.1	0.61 (0.59–0.63)	0.63 (0.61–0.65)	0.63 (0.61–0.65)	4,590 (3,560)	0.30 (0.28–0.32)
Asian or Asian British	32,245 (23,100)	78.6	0.62 (0.61–0.63)	0.66 (0.65–0.67)	0.66 (0.65–0.67)	28,185 (22,340)	0.33 (0.32–0.35)
Black or Black British	16,345 (10,035)	65.8	0.46 (0.45–0.47)	0.48 (0.47–0.49)	0.48 (0.47–0.49)	14,725 (9,780)	0.20 (0.20–0.21)
Other ethnic groups	6,270 (4,640)	81.3	0.68 (0.66–0.70)	0.69 (0.67–0.71)	0.69 (0.67–0.71)	5,440 (4,455)	0.34 (0.31–0.36)
**†IMD**							
1 most deprived (ref)	158,960 (125,290)	87.6	1.00	1.00	1.00	135,750 (119,600)	1.00
2	180,015 (149,635)	92.0	1.18 (1.17–1.19)	1.14 (1.14–1.15)	1.14 (1.13–1.15)	154,825 (143,160)	1.44 (1.40–1.48)
3	214,280 (184,140)	94.4	1.31 (1.30–1.32)	1.25 (1.24–1.26)	1.24 (1.23–1.25)	185,775 (176,125)	1.83 (1.78–1.88)
4	207,080 (180,565)	95.5	1.40 (1.39–1.41)	1.32 (1.31–1.33)	1.31 (1.30–1.32)	180,325 (172,735)	2.10 (2.03–2.16)
5 least deprived	188,510 (166,605)	96.4	1.52 (1.51–1.53)	1.43 (1.42–1.44)	1.42 (1.41–1.43)	164,995 (159,625)	2.60 (2.52–2.69)
**†Setting**							
Urban city or town (ref)	506,660 (432,255)	93.8	1.00	1.00	1.00	439,015 (413,745)	1
Urban conurbation	199,215 (160,590)	89.3	0.95 (0.94–0.96)	1.01 (1.01–1.02)	1.01 (1.00–1.02)	170,700 (153,335)	0.87 (0.85–0.90)
Rural town or village	242,975 (213,390)	96.0	1.08 (1.08–1.09)	1.01 (1.01–1.02)	1.01 (1.01–1.02)	211,955 (204,170)	1.20 (1.16–1.23)
**Kidney disease subgroup**							
CKD3a (ref)	627,265 (547,740)	93.7	1.00	1.00	1.00	563,295 (529,545)	1.00
CKD3b	236,205 (194,455)	93.8	0.92 (0.92–0.93)	0.94 (0.93–0.94)	0.93 (0.92–0.93)	193,880 (182,825)	0.94 (0.92–0.96)
CKD4–5	63,165 (46,400)	91.7	0.84 (0.83–0.85)	0.87 (0.87–0.88)	0.86 (0.85–0.87)	45,450 (42,055)	0.84 (0.81–0.88)
RRT (dialysis)	9,440 (6,880)	86.3	1.03 (1.00–1.05)	1.09 (1.07–1.12)	1.07 (1.05–1.10)	7,280 (6,335)	1.06 (0.99–1.15)
RRT (transplant)	12,770 (10,765)	88.7	1.54 (1.51–1.57)	1.45 (1.42–1.49)	1.44 (1.41–1.47)	11,765 (10,490)	1.09 (1.01–1.17)
**Primary care coding of kidney disease**							
CKD diagnostic code	563,335 (478,860)	94.1	1.03 (1.02–1.03)	1.04 (1.03–1.04)	1.06 (1.06–1.07)	482,525 (456,065)	1.23 (1.21–1.25)
**Risk group (occupation/access)**							
*†Care home resident	43,385 (27,150)	94.5	1.01 (1.00–1.02)	1.06 (1.05–1.07)	1.11 (1.10–1.12)	23,070 (21,910)	1.02 (0.96–1.09)
*†Health/social care worker	5,175 (4,770)	93.2	2.15 (2.09–2.21)	2.27 (2.21–2.34)	2.25 (2.19–2.32)	5,060 (4,730)	2.66 (2.36–2.99)
†Housebound	53,520 (37,175)	92.4	0.60 (0.59–0.60)	0.61 (0.61–0.62)	0.63 (0.62–0.63)	35,160 (32,795)	0.79 (0.76–0.83)

For peer review only - http://bmjopen.bmj.com/site/about/guidelines.xhtml

†End of life care	39,430 (23,495)	92.6	0.82 (0.81–0.83)	0.87 (0.85–0.88)	0.87 (0.86–0.88)	21,080 (19,740)	0.86 (0.81–0.91)
**Risk group (clinical)**							
†Prior SARS-CoV-2	25,490 (17,785)	90.7	0.89 (0.88–0.91)	0.94 (0.92–0.95)	0.94 (0.93–0.96)	17,785 (16,230)	0.96 (0.91–1.02)
†Immunosuppression	80,025 (67,140)	93.8	1.17 (1.16–1.18)	1.19 (1.18–1.20)	1.11 (1.10–1.12)	67,605 (63,680)	1.39 (1.34–1.45)
Moderate/severe obesity	108,950 (94,075)	92.5	0.93 (0.92–0.94)	0.97 (0.96–0.97)	0.98 (0.97–0.99)	98,525 (91,440)	1.11 (1.08–1.14)
Diabetes	269,895 (222,880)	92.6	0.91 (0.90–0.91)	0.95 (0.95–0.96)	0.96 (0.96–0.97)	227,180 (211,585)	1.06 (1.04–1.08)
CRD (inc. asthma)	210,095 (174,610)	93.5	0.98 (0.98–0.99)	1.01 (1.00–1.01)	1.01 (1.01–1.02)	176,195 (165,520)	1.05 (1.03–1.08)
Chronic heart disease	425,575 (352,775)	94.4	0.98 (0.98–0.99)	0.98 (0.97–0.98)	0.99 (0.98–0.99)	349,835 (331,995)	1.06 (1.04–1.09)
Chronic liver disease	31,285 (26,060)	91.9	1.00 (0.99–1.01)	1.01 (0.99–1.02)	1.01 (1.00–1.02)	27,045 (24,990)	1.01 (0.96–1.06)
Asplenia	8,405 (7,180)	94.3	1.10 (1.07–1.12)	1.08 (1.06–1.11)	1.08 (1.06–1.11)	7,270 (6,875)	1.19 (1.07–1.32)
Cancer (non-haematologic)	164,110 (136,440)	95.4	1.09 (1.08–1.10)	1.07 (1.06–1.08)	1.07 (1.06–1.08)	134,180 (128,730)	1.30 (1.27–1.34)
†Haematologic cancer	19,990 (16,325)	95.4	1.16 (1.14–1.18)	0.99 (0.97–1.00)	1.04 (1.03–1.06)	15,800 (15,175)	1.07 (0.98–1.18)
Organ transplant (non-kidney)	2,170 (1,865)	93.0	1.46 (1.39–1.52)	1.31 (1.26–1.38)	1.39 (1.33–1.46)	1,910 (1,795)	1.41 (1.16–1.72)
CND (inc. learning disability)	158,200 (124,130)	93.4	0.89 (0.88–0.89)	0.90 (0.90–0.91)	0.91 (0.90–0.91)	121,505 (114,275)	0.90 (0.87–0.92)
Severe mental illness	16,270 (12,665)	87.2	0.76 (0.75–0.77)	0.79 (0.78–0.81)	0.80 (0.78–0.81)	13,735 (12,055)	0.67 (0.63–0.70)
CEV (other)	5,750 (4,815)	91.6	1.01 (0.98–1.03)	1.05 (1.02–1.08)	1.03 (1.00–1.06)	4,965 (4,580)	0.93 (0.83–1.04)

Cumulative coverage was determined based on Kaplan-Meier estimates, censoring on death, deregistration, or 11th May 2022. Logistic regression models included 821,670 individuals who were uncensored (registered and alive) as of the analysis cut-off (11th May 2022). * variable included in all minimally adjusted models; † variable included in all partially adjusted models. CI, confidence interval; CKD, chronic kidney disease; HR, hazard ratio; IMD, index of multiple deprivation; OR, odds ratio; RRT, renal replacement therapy.

For peer review only - http://bmjopen.bmj.com/site/about/guidelines.xhtml

Supplementary Table 3. Cox proportional hazards models for factors associated with completion of a 3-dose vaccination series in kidney disease subgroups.

	CKD3a			CKD3b			CKD4–5			RRT (dialysis)			RRT (transplant)		
Variable	N (n events)	Cov. (%)	HR (95% CI)	N (n events)	Cov. (%)	HR (95% CI)	N (n events)	Cov. (%)	HR (95% CI)	N (n events)	Cov. (%)	HR (95% CI)	N (n events)	Cov. (%)	HR (95% CI)
***†Age**															
16–64 (ref)	71,970 (59,135)	84.5	1.00	13,245 (10,360)	82.5	1.00	5,870 (4,385)	80.7	1.00	4,390 (3,165)	79.9	1.00	9,295 (7,770)	86.7	1.00
65–69	54,435 (48,425)	92.0	1.34 (1.32–1.36)	10,815 (9,205)	90.5	1.28 (1.25–1.32)	3,020 (2,355)	88.1	1.25 (1.19–1.32)	1,095 (830)	88.0	1.26 (1.17–1.37)	1,425 (1,260)	94.3	1.38 (1.30–1.47)
70–74	102,975 (94,125)	94.8	1.80 (1.78–1.82)	24,730 (21,670)	93.8	1.62 (1.58–1.65)	5,865 (4,700)	91.2	1.45 (1.39–1.51)	1,145 (855)	90.3	1.35 (1.25–1.46)	1,180 (1,035)	94.2	1.40 (1.31–1.50)
75–79	124,030 (113,350)	95.7	2.27 (2.25–2.29)	38,245 (33,655)	95.0	2.00 (1.96–2.05)	8,410 (6,645)	93.5	1.80 (1.73–1.87)	1,220 (920)	94.5	1.64 (1.52–1.77)	620 (510)	94.0	1.47 (1.34–1.61)
80+	273,855 (232,705)	95.3	2.71 (2.69–2.74)	149,170 (119,565)	94.8	2.36 (2.31–2.41)	40,000 (28,315)	93.5	2.07 (2.00–2.14)	1,590 (1,110)	93.1	1.91 (1.78–2.05)	[R]	[R]	[R]
**†Sex**															
Female (ref)	355,460 (310,735)	93.4	1.00	134,120 (110,490)	93.4	1.00	32,700 (24,050)	91.3	1.00	3,480 (2,530)	84.7	1.00	4,925 (4,175)	88.5	1.00
Male	271,805 (237,005)	94.0	1.09 (1.08–1.09)	102,085 (83,965)	94.2	1.12 (1.11–1.14)	30,465 (22,355)	92.1	1.13 (1.11–1.15)	5,960 (4,355)	87.0	1.04 (0.99–1.09)	7,845 (6,590)	88.6	0.98 (0.95–1.02)
**†Ethnicity**															
White (ref)	590,315 (521,735)	94.9	1.00	222,900 (185,385)	94.9	1.00	57,975 (43,250)	93.5	1.00	7,295 (5,415)	88.9	1.00	10,390 (9,015)	91.2	1.00
Mixed	3,415 (2,470)	76.1	0.61 (0.59–0.64)	1,065 (770)	79.8	0.66 (0.61–0.70)	350 (220)	73.6	0.60 (0.52–0.68)	140 (105)	76.9	0.89 (0.73–1.08)	140 (100)	71.4	0.65 (0.54–0.80)
Asian or Asian British	18,720 (13,805)	79.0	0.64 (0.63–0.65)	7,595 (5,310)	78.3	0.65 (0.64–0.67)	3,120 (1,915)	74.7	0.65 (0.62–0.68)	1,295 (920)	80.6	0.85 (0.78–0.91)	1,510 (1,145)	79.4	0.72 (0.67–0.77)
Black or Black British	10,840 (6,710)	65.1	0.48 (0.46–0.49)	3,240 (1,965)	66.8	0.47 (0.45–0.50)	1,225 (695)	65.5	0.49 (0.45–0.53)	570 (355)	67.5	0.60 (0.54–0.68)	470 (310)	68.9	0.54 (0.48–0.60)
Other ethnic groups	3,975 (3,015)	81.6	0.68 (0.65–0.70)	1,405 (1,025)	82.0	0.71 (0.67–0.76)	495 (320)	76.1	0.70 (0.63–0.78)	135 (85)	66.2	0.67 (0.54–0.83)	260 (190)	76.7	0.71 (0.61–0.82)
**†IMD**															
1 most deprived (ref)	100,965 (82,025)	87.9	1.00	40,770 (31,390)	88.5	1.00	12,015 (8,120)	84.9	1.00	2,610 (1,770)	78.8	1.00	2,600 (1,990)	80.2	1.00
2	116,850 (99,945)	92.2	1.15 (1.14–1.16)	45,630 (36,860)	92.5	1.12 (1.11–1.14)	12,685 (9,090)	90.2	1.16 (1.12–1.19)	2,185 (1,555)	84.8	1.21 (1.13–1.30)	2,665 (2,185)	86.0	1.22 (1.15–1.30)
3	141,655 (124,995)	94.6	1.24 (1.23–1.26)	53,770 (44,755)	94.8	1.23 (1.21–1.25)	14,205 (10,595)	92.9	1.26 (1.22–1.30)	1,890 (1,425)	88.2	1.31 (1.21–1.41)	2,760 (2,370)	90.2	1.38 (1.30–1.47)
4	139,220 (124,375)	95.6	1.32 (1.31–1.33)	50,790 (42,810)	95.6	1.30 (1.28–1.32)	13,015 (9,975)	94.5	1.33 (1.29–1.37)	1,530 (1,195)	91.2	1.41 (1.30–1.52)	2,520 (2,210)	92.2	1.52 (1.43–1.62)
5 least deprived	128,575 (116,400)	96.6	1.44 (1.42–1.45)	45,240 (38,635)	96.4	1.40 (1.37–1.42)	11,245 (8,625)	95.4	1.43 (1.38–1.48)	1,230 (935)	91.6	1.46 (1.34–1.59)	2,225 (2,010)	93.8	1.62 (1.51–1.73)
**†Setting**															
Urban city or town (ref)	334,925 (293,565)	94.0	1.00	126,760 (104,820)	94.2	1.00	33,625 (24,845)	92.4	1.00	4,735 (3,400)	86.0	1.00	6,615 (5,630)	89.3	1.00
Urban conurbation	128,630 (106,790)	89.7	1.01 (1.00–1.02)	49,545 (38,800)	89.8	1.02 (1.00–1.03)	14,540 (10,095)	86.3	1.00 (0.97–1.03)	3,000 (2,135)	82.4	1.12 (1.03–1.21)	3,495 (2,770)	83.1	1.06 (1.00–1.13)
Rural town or village	163,705 (147,385)	96.1	1.01 (1.00–1.02)	59,900 (50,830)	96.1	1.01 (1.00–1.02)	15,000 (11,460)	95.2	1.03 (1.01–1.05)	1,705 (1,345)	93.2	1.18 (1.10–1.26)	2,660 (2,370)	93.5	1.05 (1.00–1.10)
**Primary care coding of kidney disease**															
CKD diagnostic code	306,820 (270,530)	94.5	1.05 (1.04–1.05)	183,225 (152,815)	94.3	1.08 (1.07–1.09)	55,645 (41,425)	92.2	1.17 (1.13–1.20)	7,990 (5,895)	87.6	1.14 (1.07–1.22)	9,655 (8,200)	89.6	1.10 (1.05–1.15)
**Risk group (occupation/access)**															
*†Care home resident	23,700 (15,635)	94.5	1.03 (1.02–1.05)	14,845 (8,955)	94.6	1.09 (1.06–1.11)	4,605 (2,425)	93.2	1.14 (1.09–1.19)	[R]	[R]	[R]	[R]	[R]	[R]
*†Health/social care worker	3,975 (3,680)	93.5	2.35 (2.27–2.43)	585 (530)	89.7	2.03 (1.87–2.22)	195 (170)	84.2	2.08 (1.79–2.42)	[R]	[R]	[R]	355 (335)	91.7	2.09 (1.87–2.33)
†Housebound	26,735 (19,485)	92.7	0.60 (0.59–0.61)	18,770 (12,815)	92.4	0.63 (0.62–0.64)	6,800 (3,985)	90.4	0.65 (0.63–0.67)	600 (370)	88.4	0.91 (0.82–1.01)	615 (520)	89.2	0.92 (0.84–1.00)
†End of life care	20,070 (12,665)	92.9	0.86 (0.85–0.88)	13,185 (7,700)	92.9	0.89 (0.87–0.91)	5,385 (2,640)	90.7	0.88 (0.85–0.92)	600 (355)	84.7	0.86 (0.77–0.96)	[R]	[R]	[R]
**Risk group (clinical)**															
†Prior SARS-CoV-2	14,290 (10,625)	91.4	0.93 (0.92–0.95)	7,135 (4,690)	91.5	0.94 (0.91–0.97)	2,545 (1,400)	88.1	0.92 (0.87–0.97)	1,000 (670)	82.0	0.96 (0.88–1.04)	515 (400)	81.5	0.91 (0.82–1.00)
Immunosuppression	43,575 (37,735)	94.9	1.12 (1.11–1.14)	18,245 (14,895)	94.8	1.12 (1.10–1.15)	5,525 (3,980)	92.7	1.16 (1.11–1.20)	2,065 (1,540)	85.6	1.08 (1.02–1.15)	10,615 (8,995)	89.1	1.06 (1.01–1.12)
Moderate/severe obesity	71,070 (62,710)	92.6	0.97 (0.97–0.98)	27,390 (23,215)	92.9	0.97 (0.96–0.98)	8,000 (6,145)	90.7	0.97 (0.95–1.00)	1,325 (1,005)	87.5	1.09 (1.01–1.16)	1,160 (995)	89.0	1.07 (1.00–1.14)
Diabetes	152,745 (130,485)	92.8	0.95 (0.95–0.96)	81,390 (66,145)	92.9	0.96 (0.96–0.97)	27,680 (20,000)	91.0	0.98 (0.96–1.00)	4,040 (2,860)	87.5	1.03 (0.98–1.09)	4,040 (3,390)	89.9	1.07 (1.02–1.11)
CRD (inc. asthma)	136,395 (116,745)	93.6	1.01 (1.00–1.01)	54,735 (44,070)	93.9	1.02 (1.01–1.03)	14,675 (10,480)	91.7	1.01 (0.98–1.03)	2,035 (1,440)	86.9	1.03 (0.98–1.10)	2,255 (1,880)	88.0	0.96 (0.91–1.01)
Chronic heart disease	255,835 (219,880)	94.7	0.98 (0.98–0.99)	125,250 (100,865)	94.4	0.98 (0.97–0.99)	35,895 (25,480)	92.8	1.00 (0.98–1.02)	4,740 (3,355)	87.7	0.98 (0.94–1.03)	3,860 (3,195)	90.1	1.01 (0.96–1.05)
Chronic liver disease	20,250 (17,365)	92.1	1.01 (1.00–1.03)	7,650 (6,190)	92.3	1.01 (0.98–1.04)	2,245 (1,630)	90.3	1.01 (0.97–1.07)	550 (380)	80.5	1.00 (0.90–1.11)	585 (490)	88.8	1.07 (0.98–1.17)
Asplenia	5,485 (4,835)	94.7	1.10 (1.07–1.13)	2,035 (1,695)	93.5	1.10 (1.05–1.15)	575 (400)	87.4	0.97 (0.88–1.07)	140 (100)	83.3	1.09 (0.89–1.32)	[R]	[R]	[R]
Cancer (non-haematologic)	103,955 (89,290)	95.6	1.07 (1.07–1.08)	45,090 (36,250)	95.5	1.06 (1.05–1.08)	12,800 (9,115)	94.1	1.09 (1.06–1.11)	1,305 (965)	91.4	1.12 (1.05–1.21)	960 (820)	92.0	1.14 (1.06–1.22)
Haematologic cancer	11,885 (10,075)	95.6	1.03 (1.01–1.06)	5,705 (4,560)	95.5	1.05 (1.01–1.09)	1,845 (1,265)	92.7	0.99 (0.93–1.06)	[R]	[R]	[R]	240 (205)	89.6	1.11 (0.97–1.28)
Organ transplant (non-kidney)	1,195 (1,055)	92.0	1.39 (1.30–1.47)	685 (575)	93.0	1.36 (1.25–1.48)	[R]	[R]	[R]	[R]	[R]	[R]	[N]	[N]	[N]
CND (inc. learning disability)	96,415 (78,640)	93.7	0.90 (0.89–0.91)	46,255 (34,965)	93.4	0.91 (0.90–0.92)	12,905 (8,540)	91.6	0.92 (0.90–0.95)	1,440 (995)	86.0	0.92 (0.86–0.98)	1,185 (985)	89.2	0.96 (0.90–1.03)
Severe mental illness	10,465 (8,255)	86.4	0.77 (0.75–0.78)	4,105 (3,205)	89.6	0.85 (0.82–0.88)	1,310 (925)	87.1	0.91 (0.85–0.97)	220 (145)	72.2	0.83 (0.70–0.98)	170 (135)	81.2	0.85 (0.72–1.01)
CEV (other)	3,480 (2,990)	92.1	1.03 (0.99–1.07)	1,465 (1,195)	92.1	1.09 (1.03–1.16)	810 (630)	87.3	1.09 (1.01–1.18)	[N]	[N]	[N]	[N]	[N]	[N]

Counts are rounded to the nearest 5. Cumulative coverage was determined based on Kaplan-Meier estimates, censoring on death, deregistration, or 11th May 2022. Hazard ratios and 95% confidence intervals were derived from partially adjusted models that included age, care home residence, health and social care worker status, housebound status, receipt of end-of-life care, setting (urban/rural), sex, ethnicity, IMD quintile, prior SARS-CoV-2 infection, immunosuppression, and haematologic cancer. Data are redacted [R] for any rows in which there were >0 and ≤10 event or non-event counts after rounding to the nearest 5, with non-event counts calculated among uncensored individuals. CEV, clinically extremely vulnerable; CKD, chronic kidney disease; CND, chronic neurological disease; IMD, index of multiple deprivation; [N], covariate absent in all individuals by definition; [R] redacted; RRT, renal replacement therapy.

For peer review only - http://bmjopen.bmj.com/site/about/guidelines.xhtml

Supplementary Table 4. Baseline characteristics of secondary outcome analysis population (dose 4 uptake).

Characteristic	All N = 679,405	CKD3a N = 418,095	CKD3b N = 193,475	CKD4–5 N = 50,465	RRT (dialysis) N = 4,595	RRT (transplant) N = 12,770
**Age**						
16–64	20,015 (2.9%)	6,775 (1.6%)	1,845 (1.0%)	800 (1.6%)	1,300 (28.3%)	9,295 (72.8%)
65–69	8,210 (1.2%)	4,705 (1.1%)	1,395 (0.7%)	435 (0.9%)	245 (5.3%)	1,425 (11.2%)
70–74	13,795 (2.0%)	8,730 (2.1%)	2,815 (1.5%)	825 (1.6%)	245 (5.3%)	1,180 (9.2%)
75–79	172,530 (25.4%)	124,030 (29.7%)	38,245 (19.8%)	8,410 (16.7%)	1,220 (26.6%)	620 (4.9%)
80+	464,860 (68.4%)	273,855 (65.5%)	149,170 (77.1%)	40,000 (79.3%)	1,590 (34.6%)	250 (2.0%)
**Sex**						
Female	387,500 (57.0%)	241,015 (57.6%)	112,810 (58.3%)	27,035 (53.6%)	1,710 (37.2%)	4,925 (38.6%)
Male	291,905 (43.0%)	177,080 (42.4%)	80,665 (41.7%)	23,430 (46.4%)	2,885 (62.8%)	7,845 (61.4%)
**Ethnicity**						
White	645,830 (95.1%)	399,635 (95.6%)	184,715 (95.5%)	47,335 (93.8%)	3,755 (81.7%)	10,390 (81.4%)
Mixed	2,740 (0.4%)	1,620 (0.4%)	710 (0.4%)	215 (0.4%)	60 (1.3%)	140 (1.1%)
Asian or Asian British	18,695 (2.8%)	9,955 (2.4%)	4,920 (2.5%)	1,820 (3.6%)	490 (10.7%)	1,510 (11.8%)
Black or Black British	8,275 (1.2%)	4,630 (1.1%)	2,155 (1.1%)	780 (1.5%)	235 (5.1%)	470 (3.7%)
Other ethnic groups	3,865 (0.6%)	2,255 (0.5%)	980 (0.5%)	315 (0.6%)	55 (1.2%)	260 (2.0%)
**Index of multiple deprivation quintile**						
1 most deprived	103,695 (15.3%)	60,420 (14.5%)	30,950 (16.0%)	8,705 (17.2%)	1,015 (22.1%)	2,600 (20.4%)
2	124,970 (18.4%)	74,870 (17.9%)	36,495 (18.9%)	9,935 (19.7%)	1,005 (21.9%)	2,665 (20.9%)
3	155,525 (22.9%)	95,730 (22.9%)	44,525 (23.0%)	11,530 (22.8%)	980 (21.3%)	2,760 (21.6%)
4	152,900 (22.5%)	95,975 (23.0%)	42,745 (22.1%)	10,810 (21.4%)	845 (18.4%)	2,520 (19.7%)
5 least deprived	142,315 (20.9%)	91,100 (21.8%)	38,755 (20.0%)	9,485 (18.8%)	745 (16.2%)	2,225 (17.4%)
**Setting**						
Urban city or town	362,390 (53.3%)	222,365 (53.2%)	103,970 (53.7%)	27,115 (53.7%)	2,320 (50.5%)	6,615 (51.8%)
Urban conurbation	138,875 (20.4%)	83,815 (20.0%)	39,355 (20.3%)	10,895 (21.6%)	1,320 (28.7%)	3,495 (27.4%)
Rural	178,145 (26.2%)	111,915 (26.8%)	50,150 (25.9%)	12,460 (24.7%)	960 (20.9%)	2,660 (20.8%)
**Primary care coding of kidney disease**						
CKD3–5 diagnostic code	424,480 (62.5%)	214,160 (51.2%)	151,790 (78.5%)	44,890 (89.0%)	3,985 (86.7%)	9,655 (75.6%)
Dialysis code	12,025 (1.8%)	0 (0.0%)	0 (0.0%)	0 (0.0%)	3,720 (81.0%)	8,305 (65.0%)
Kidney transplant code	13,435 (2.0%)	0 (0.0%)	0 (0.0%)	0 (0.0%)	1,070 (23.3%)	12,360 (96.8%)
**Risk group (occupation/access)**						
Care home resident	43,385 (6.4%)	23,700 (5.7%)	14,845 (7.7%)	4,605 (9.1%)	165 (3.6%)	65 (0.5%)
Health/social care worker	1,095 (0.2%)	565 (0.1%)	120 (0.1%)	35 (0.1%)	20 (0.4%)	355 (2.8%)
Housebound	49,420 (7.3%)	24,345 (5.8%)	17,740 (9.2%)	6,345 (12.6%)	375 (8.2%)	615 (4.8%)
End of life care	35,765 (5.3%)	17,935 (4.3%)	12,290 (6.4%)	4,995 (9.9%)	365 (7.9%)	180 (1.4%)
**Risk group (clinical)**						
Prior SARS-CoV-2 *	19,170 (2.8%)	10,080 (2.4%)	6,040 (3.1%)	2,065 (4.1%)	475 (10.3%)	515 (4.0%)
Immunosuppression	80,025 (11.8%)	43,575 (10.4%)	18,245 (9.4%)	5,525 (10.9%)	2,065 (44.9%)	10,615 (83.1%)
Moderate/severe obesity	55,040 (8.1%)	31,670 (7.6%)	16,970 (8.8%)	4,880 (9.7%)	360 (7.8%)	1,160 (9.1%)
Diabetes	190,140 (28.0%)	100,110 (23.9%)	62,990 (32.6%)	21,315 (42.2%)	1,685 (36.7%)	4,040 (31.6%)
Chronic respiratory disease (inc. asthma)	151,400 (22.3%)	91,725 (21.9%)	44,605 (23.1%)	11,810 (23.4%)	1,005 (21.9%)	2,255 (17.7%)
Chronic heart disease	340,605 (50.1%)	195,385 (46.7%)	107,965 (55.8%)	30,860 (61.2%)	2,535 (55.2%)	3,860 (30.2%)

For peer review only

Chronic liver disease	18,715 (2.8%)	11,090 (2.7%)	5,290 (2.7%)	1,505 (3.0%)	240 (5.2%)	585 (4.6%)
Asplenia	5,930 (0.9%)	3,650 (0.9%)	1,575 (0.8%)	455 (0.9%)	80 (1.7%)	170 (1.3%)
Cancer (non-haematologic)	128,475 (18.9%)	77,375 (18.5%)	38,315 (19.8%)	11,020 (21.8%)	805 (17.5%)	960 (7.5%)
Haematologic cancer	19,990 (2.9%)	11,885 (2.8%)	5,705 (2.9%)	1,845 (3.7%)	310 (6.7%)	240 (1.9%)
Organ transplant (non-kidney) †	2,170 (0.3%)	1,195 (0.3%)	685 (0.4%)	215 (0.4%)	75 (1.6%)	[N]
CND (inc. learning disability)	131,900 (19.4%)	77,535 (18.5%)	41,125 (21.3%)	11,295 (22.4%)	765 (16.6%)	1,185 (9.3%)
Severe mental illness	9,190 (1.4%)	5,405 (1.3%)	2,700 (1.4%)	835 (1.7%)	80 (1.7%)	170 (1.3%)
Clinically extremely vulnerable (other) §	3,990 (0.6%)	2,370 (0.6%)	1,170 (0.6%)	450 (0.9%)	[N]	[N]
**Region**						
London	20,255 (3.0%)	11,740 (2.8%)	5,490 (2.8%)	1,800 (3.6%)	315 (6.9%)	910 (7.1%)
East of England	152,635 (22.5%)	94,895 (22.7%)	42,880 (22.2%)	10,840 (21.5%)	1,055 (23.0%)	2,970 (23.3%)
East Midlands	125,850 (18.5%)	77,615 (18.6%)	35,975 (18.6%)	9,215 (18.3%)	790 (17.2%)	2,250 (17.6%)
North East	31,955 (4.7%)	19,750 (4.7%)	9,150 (4.7%)	2,215 (4.4%)	240 (5.2%)	595 (4.7%)
North West	62,430 (9.2%)	38,315 (9.2%)	17,940 (9.3%)	4,700 (9.3%)	305 (6.6%)	1,165 (9.1%)
South East	42,580 (6.3%)	26,645 (6.4%)	11,810 (6.1%)	3,100 (6.1%)	290 (6.3%)	735 (5.8%)
South West	122,945 (18.1%)	76,210 (18.2%)	35,105 (18.1%)	9,145 (18.1%)	695 (15.1%)	1,790 (14.0%)
West Midlands	24,805 (3.7%)	14,830 (3.5%)	7,255 (3.7%)	1,975 (3.9%)	275 (6.0%)	465 (3.6%)
Yorkshire and the Humber	95,950 (14.1%)	58,095 (13.9%)	27,870 (14.4%)	7,480 (14.8%)	620 (13.5%)	1,885 (14.8%)

Individuals were included in the analysis if they were ≥75 years of age at baseline, care home residents, transplant recipients, or had a history of haematologic malignancy or immunosuppression. Data are n (%) after rounding to the nearest 5. Primary care codes and risk groups are coded by separate binary variables; percentages under these table subheadings therefore do not sum to 100. Among people with CKD4–5, 46,830 had stage 4 CKD (eGFR 15–29 ml/min/1.73 m^2) and 3,635 had stage 5 CKD (eGFR <15ml/min/1.73 m^2). CKD, chronic kidney disease; CND, chronic neurological disease; [N], covariate absent in all individuals by definition; RRT, renal replacement therapy. * Based on prior evidence of a positive SARS-CoV-2 test, COVID-19-related primary care code, or COVID-19-related hospitalisation as of 1st December 2020; † Excludes individuals with kidney transplants based on primary care coding or UK Renal Registry status; § Classified as clinically extremely vulnerable in the absence of any of the comorbidities listed above (including RRT).

For peer review only - http://bmjopen.bmj.com/site/about/guidelines.xhtml

Supplementary Table 5. Cox proportional hazards models for factors associated with completion of a 4-dose vaccination series in people with kidney disease.

Variable	N (n events)	Cov. (%)	HR (95% CI), minimally adjusted	HR (95% CI), partially adjusted	HR (95% CI), fully adjusted
***†Age**					
16–64 (ref)	20,015 (8,005)	42.5	1.00	1.00	1.00
65–69	8,210 (3,340)	44.9	1.08 (1.04-1.12)	0.99 (0.95-1.03)	1.52 (1.45-1.58)
70–74	13,795 (6,090)	50.0	1.22 (1.18-1.26)	1.06 (1.03-1.10)	1.80 (1.73-1.87)
75–79	172,530 (99,315)	62.8	1.38 (1.35-1.41)	1.51 (1.47-1.54)	2.76 (2.67-2.85)
80+	464,860 (232,695)	61.9	1.41 (1.38-1.45)	1.59 (1.55-1.64)	2.95 (2.86-3.05)
**†Sex**					
Female (ref)	387,500 (192,935)	58.6	1.00	1.00	1.00
Male	291,905 (156,510)	64.4	1.19 (1.18-1.19)	1.17 (1.16-1.17)	1.16 (1.16-1.17)
**†Ethnicity**					
White (ref)	645,830 (340,050)	62.5	1.00	1.00	1.00
Mixed	2,740 (875)	36.9	0.51 (0.47-0.54)	0.53 (0.50-0.57)	0.54 (0.50-0.57)
Asian or Asian British	18,695 (5,205)	32.8	0.44 (0.43-0.45)	0.48 (0.46-0.49)	0.47 (0.45-0.48)
Black or Black British	8,275 (1,805)	25.1	0.32 (0.31-0.34)	0.35 (0.33-0.37)	0.36 (0.34-0.37)
Other ethnic groups	3,865 (1,510)	46.5	0.69 (0.66-0.73)	0.71 (0.68-0.75)	0.69 (0.66-0.73)
**†IMD**					
1 most deprived (ref)	103,695 (41,680)	48.9	1.00	1.00	1.00
2	124,970 (58,805)	56.5	1.23 (1.22-1.25)	1.19 (1.18-1.21)	1.19 (1.17-1.20)
3	155,525 (81,110)	61.8	1.40 (1.38-1.41)	1.32 (1.30-1.34)	1.31 (1.29-1.33)
4	152,900 (84,305)	64.8	1.51 (1.49-1.53)	1.42 (1.40-1.44)	1.40 (1.39-1.42)
5 least deprived	142,315 (83,545)	68.6	1.65 (1.63-1.67)	1.54 (1.53-1.56)	1.52 (1.50-1.54)
**†Setting**					
Urban city or town (ref)	362,390 (186,715)	61.2	1.00	1.00	1.00
Urban conurbation	138,875 (64,560)	55.9	0.96 (0.95-0.97)	1.02 (1.01-1.03)	1.01 (1.00-1.03)
Rural town or village	178,145 (98,170)	64.7	1.08 (1.07-1.09)	1.00 (0.99-1.01)	1.00 (0.99-1.01)
**Kidney disease subgroup**					
CKD3a (ref)	418,095 (228,710)	62.5	1.00	1.00	1.00
CKD3b	193,475 (92,595)	59.3	0.91 (0.90-0.91)	0.92 (0.92-0.93)	0.92 (0.91-0.93)
CKD4–5	50,465 (19,400)	55.2	0.83 (0.82-0.84)	0.86 (0.85-0.87)	0.86 (0.85-0.87)
RRT (dialysis)	4,595 (1,715)	50.0	1.10 (1.05-1.15)	1.19 (1.13-1.24)	1.18 (1.12-1.23)
RRT (transplant)	12,770 (7,025)	58.9	2.71 (2.62-2.80)	2.96 (2.86-3.06)	3.08 (2.98-3.18)
**Primary care coding of kidney disease**					
CKD diagnostic code	424,480 (217,290)	61.2	1.00 (1.00-1.01)	1.02 (1.01-1.02)	1.04 (1.04-1.05)
**Risk group (occupation/access)**					
*†Care home resident	43,385 (11,695)	49.5	0.67 (0.65-0.68)	0.73 (0.72-0.74)	0.79 (0.77-0.80)
*†Health/social care worker	1,095 (645)	59.6	1.46 (1.35-1.58)	1.50 (1.39-1.63)	1.46 (1.35-1.58)
†Housebound	49,420 (14,125)	43.3	0.57 (0.56-0.58)	0.60 (0.59-0.61)	0.61 (0.60-0.62)
†End of life care	35,765 (9,855)	51.4	0.83 (0.82-0.85)	0.88 (0.86-0.89)	0.88 (0.86-0.90)
**Risk group (clinical)**					
†Prior SARS-CoV-2	19,170 (6,395)	51.5	0.86 (0.84-0.89)	0.92 (0.90-0.94)	0.93 (0.90-0.95)
†Immunosuppression	80,025 (38,925)	56.7	1.32 (1.30-1.33)	1.26 (1.24-1.28)	1.21 (1.20-1.23)
Moderate/severe obesity	55,040 (26,605)	55.1	0.84 (0.83-0.85)	0.89 (0.88-0.90)	0.92 (0.91-0.93)
Diabetes	190,140 (89,055)	57.2	0.87 (0.87-0.88)	0.92 (0.91-0.93)	0.93 (0.92-0.94)
CRD (inc. asthma)	151,400 (73,560)	59.6	0.97 (0.96-0.98)	1.00 (0.99-1.01)	1.01 (1.00-1.02)
Chronic heart disease	340,605 (167,470)	60.9	0.97 (0.97-0.98)	0.96 (0.95-0.97)	0.97 (0.97-0.98)
Chronic liver disease	18,715 (9,190)	58.3	1.01 (0.99-1.03)	1.03 (1.01-1.05)	1.03 (1.01-1.05)
Asplenia	5,930 (3,175)	63.2	1.12 (1.08-1.16)	1.09 (1.05-1.12)	1.08 (1.05-1.12)
Cancer (non-haematologic)	128,475 (66,290)	64.3	1.10 (1.09-1.11)	1.07 (1.06-1.08)	1.07 (1.06-1.08)
†Haematologic cancer	19,990 (10,400)	64.4	1.47 (1.44-1.50)	1.23 (1.20-1.26)	1.33 (1.30-1.36)
Organ transplant (non-kidney)	2,170 (1,210)	62.3	1.81 (1.71-1.92)	1.82 (1.72-1.93)	2.41 (2.28-2.56)
CND (inc. learning disability)	131,900 (55,625)	56.2	0.86 (0.85-0.87)	0.87 (0.86-0.88)	0.88 (0.87-0.89)
Severe mental illness	9,190 (3,195)	44.0	0.67 (0.65-0.69)	0.72 (0.69-0.74)	0.74 (0.71-0.76)
CEV (other)	3,990 (2,045)	61.2	1.04 (1.00-1.09)	1.10 (1.05-1.15)	1.05 (1.01-1.10)

Cumulative coverage was determined based on Kaplan-Meier estimates, censoring on death, deregistration, or 11th May 2022. * variable included in all minimally adjusted models; † variable included in all partially adjusted models. CI, confidence interval; CKD, chronic kidney disease; HR, hazard ratio; IMD, index of multiple deprivation; OR, odds ratio; RRT, renal replacement therapy.

For peer review only - http://bmjopen.bmj.com/site/about/guidelines.xhtml

Supplementary Table 6. Cox proportional hazards models for factors associated with completion of a 3-dose vaccination series in kidney disease subgroups.

	CKD3a			CKD3b			CKD4–5			RRT (dialysis)			RRT (transplant)		
**Variable**	**N (n events)**	**Cov. (%)**	**HR (95% CI)**	**N (n events)**	**Cov. (%)**	**HR (95% CI)**	**N (n events)**	**Cov. (%)**	**HR (95% CI)**	**N (n events)**	**Cov. (%)**	**HR (95% CI)**	**N (n events)**	**Cov. (%)**	**HR (95% CI)**
**Age**															
16–64 (ref)	6,775 (2,000)	31.0	1.00	1,845 (585)	34.4	1.00	800 (255)	35.4	1.00	1,300 (440)	37.3	1.00	9,295 (4,730)	53.3	1.00
65–69	4,705 (1,630)	37.2	1.21 (1.13–1.29)	1,395 (515)	41.3	1.21 (1.08–1.37)	435 (165)	43.7	1.36 (1.11–1.65)	245 (85)	40.8	1.22 (0.97–1.54)	1,425 (945)	72.1	1.60 (1.49–1.72)
70–74	8,730 (3,790)	47.9	1.61 (1.52–1.70)	2,815 (1,105)	46.0	1.27 (1.15–1.41)	825 (305)	48.1	1.37 (1.16–1.62)	245 (90)	48.1	1.35 (1.07–1.70)	1,180 (800)	75.9	1.67 (1.55–1.81)
75–79	124,030 (74,145)	63.9	2.58 (2.46–2.71)	38,245 (20,495)	59.9	1.97 (1.81–2.15)	8,410 (3,765)	56.3	1.76 (1.54–2.02)	1,220 (495)	55.6	1.45 (1.19–1.75)	620 (410)	76.2	1.67 (1.51–1.85)
80+	273,855 (147,145)	63.7	2.79 (2.66–2.92)	149,170 (69,900)	59.9	2.12 (1.95–2.30)	40,000 (14,910)	55.7	1.82 (1.59–2.08)	1,590 (605)	58.1	1.62 (1.33–1.96)	250 (140)	73.5	1.68 (1.42–1.99)
**Sex**															
Female (ref)	241,015 (127,790)	60.2	1.00	112,810 (51,900)	56.4	1.00	27,035 (9,830)	51.4	1.00	1,710 (585)	44.9	1.00	4,925 (2,825)	60.8	1.00
Male	177,080 (100,920)	65.8	1.15 (1.14–1.16)	80,665 (40,695)	63.3	1.19 (1.18–1.21)	23,430 (9,565)	59.8	1.24 (1.20–1.27)	2,885 (1,130)	52.8	1.19 (1.07–1.31)	7,845 (4,200)	57.7	0.92 (0.87–0.96)
**Ethnicity**															
White (ref)	399,635 (223,240)	63.9	1.00	184,715 (90,355)	60.6	1.00	47,335 (18,735)	57.0	1.00	3,755 (1,480)	54.0	1.00	10,390 (6,240)	64.3	1.00
Mixed	1,620 (550)	38.1	0.54 (0.50–0.59)	710 (210)	35.5	0.51 (0.45–0.58)	215 (55)	33.3	0.59 (0.45–0.77)	60 (15)	20.0	0.59 (0.35–0.97)	140 (45)	30.8	0.49 (0.36–0.65)
Asian or Asian British	9,955 (2,965)	33.8	0.46 (0.45–0.48)	4,920 (1,220)	30.5	0.45 (0.43–0.48)	1,820 (360)	28.1	0.46 (0.41–0.51)	490 (140)	35.6	0.59 (0.49–0.71)	1,510 (515)	36.1	0.51 (0.47–0.56)
Black or Black British	4,630 (1,020)	24.4	0.34 (0.32–0.36)	2,155 (455)	24.9	0.37 (0.33–0.40)	780 (150)	23.1	0.39 (0.33–0.46)	235 (60)	32.6	0.53 (0.40–0.69)	470 (120)	27.3	0.38 (0.32–0.46)
Other ethnic groups	2,255 (935)	47.4	0.70 (0.66–0.75)	980 (355)	44.2	0.70 (0.63–0.78)	315 (100)	42.8	0.75 (0.61–0.91)	55 (15)	20.0	0.59 (0.35–0.98)	260 (105)	41.7	0.63 (0.51–0.76)
**IMD**															
1 most deprived (ref)	60,420 (26,045)	50.4	1.00	30,950 (11,780)	48.2	1.00	8,705 (2,590)	43.7	1.00	1,015 (285)	36.5	1.00	2,600 (985)	40.3	1.00
2	74,870 (37,585)	58.0	1.19 (1.17–1.21)	36,495 (16,045)	55.0	1.17 (1.14–1.20)	9,935 (3,525)	51.5	1.19 (1.13–1.25)	1,005 (355)	47.4	1.40 (1.19–1.64)	2,665 (1,290)	51.5	1.37 (1.26–1.49)
3	95,730 (52,840)	63.0	1.31 (1.29–1.33)	44,525 (21,715)	60.2	1.30 (1.27–1.33)	11,530 (4,530)	56.3	1.30 (1.23–1.37)	980 (395)	53.1	1.54 (1.31–1.81)	2,760 (1,625)	63.0	1.69 (1.55–1.83)
4	95,975 (55,950)	66.1	1.41 (1.39–1.43)	42,745 (21,840)	62.8	1.39 (1.36–1.42)	10,810 (4,560)	59.3	1.40 (1.33–1.47)	845 (355)	55.4	1.61 (1.37–1.91)	2,520 (1,605)	67.9	1.86 (1.71–2.02)
5 least deprived	91,100 (56,295)	69.7	1.53 (1.51–1.55)	38,755 (21,215)	66.6	1.51 (1.48–1.55)	9,485 (4,195)	62.8	1.49 (1.42–1.57)	745 (320)	57.7	1.73 (1.46–2.05)	2,225 (1,525)	72.7	2.05 (1.88–2.23)
**Setting**															
Urban city or town (ref)	222,365 (121,865)	62.7	1.00	103,970 (50,030)	59.6	1.00	27,115 (10,385)	55.1	1.00	2,320 (805)	46.6	1.00	6,615 (3,630)	58.8	1.00
Urban conurbation	83,815 (41,785)	57.7	1.02 (1.01–1.04)	39,355 (16,900)	53.9	1.01 (0.99–1.03)	10,895 (3,770)	50.1	1.04 (0.99–1.09)	1,320 (480)	47.4	1.08 (0.92–1.26)	3,495 (1,625)	49.5	1.05 (0.97–1.14)
Rural town or village	111,915 (65,065)	65.8	1.00 (0.99–1.01)	50,150 (25,665)	62.7	0.99 (0.98–1.01)	12,460 (5,245)	60.0	1.03 (0.99–1.06)	960 (430)	59.9	1.19 (1.06–1.35)	2,660 (1,770)	71.0	1.17 (1.10–1.23)
**Primary care coding of kidney disease**															
CKD diagnostic code	214,160 (118,570)	63.2	1.03 (1.02–1.03)	151,790 (74,260)	59.9	1.07 (1.05–1.08)	44,890 (17,535)	55.6	1.06 (1.01–1.12)	3,985 (1,485)	50.7	0.98 (0.85–1.14)	9,655 (5,435)	60.6	1.13 (1.07–1.19)
**Risk group (occupation/access)**															
Care home resident	23,700 (6,855)	49.5	0.72 (0.70–0.74)	14,845 (3,800)	49.4	0.76 (0.74–0.79)	4,605 (995)	49.9	0.86 (0.80–0.92)	165 (30)	22.2	0.64 (0.44–0.94)	65 (20)	33.3	0.46 (0.30–0.72)
Health/social care worker	565 (325)	58.1	1.53 (1.37–1.70)	120 (70)	50.0	1.36 (1.08–1.73)	[R]	[R]	[R]	[R]	[R]	[R]	355 (225)	61.1	1.31 (1.15–1.50)
Housebound	24,345 (7,520)	44.2	0.59 (0.58–0.60)	17,740 (4,835)	42.1	0.59 (0.58–0.61)	6,345 (1,320)	38.8	0.60 (0.57–0.64)	375 (100)	44.9	0.88 (0.72–1.08)	615 (345)	61.5	0.95 (0.85–1.06)
End of life care	17,935 (5,395)	52.4	0.87 (0.84–0.89)	12,290 (3,240)	50.5	0.89 (0.86–0.92)	4,995 (1,035)	48.5	0.92 (0.86–0.98)	365 (90)	43.5	0.77 (0.63–0.96)	180 (90)	68.3	1.26 (1.02–1.56)
**Risk group (clinical)**															
Prior SARS-CoV-2	10,080 (3,705)	53.2	0.92 (0.89–0.95)	6,040 (1,840)	50.4	0.92 (0.87–0.96)	2,065 (485)	47.0	0.91 (0.83–1.00)	475 (140)	41.7	0.85 (0.71–1.02)	515 (230)	46.7	0.88 (0.77–1.00)
Immunosuppression	43,575 (21,400)	55.8	1.22 (1.19–1.24)	18,245 (8,595)	58.1	1.26 (1.22–1.29)	5,525 (2,200)	55.9	1.34 (1.26–1.42)	2,065 (750)	44.2	1.05 (0.88–1.25)	10,615 (5,975)	60.3	1.17 (1.10–1.26)
Moderate/severe obesity	31,670 (16,170)	56.1	0.90 (0.89–0.92)	16,970 (7,815)	54.1	0.91 (0.89–0.93)	4,880 (1,865)	50.4	0.92 (0.87–0.96)	360 (130)	45.9	0.99 (0.83–1.19)	1,160 (630)	56.7	1.03 (0.95–1.12)
Diabetes	100,110 (50,045)	58.2	0.91 (0.90–0.92)	62,990 (28,520)	56.6	0.94 (0.93–0.96)	21,315 (7,790)	53.1	0.95 (0.92–0.98)	1,685 (555)	48.2	0.96 (0.86–1.06)	4,040 (2,145)	58.4	1.03 (0.98–1.09)
CRD (inc. asthma)	91,725 (47,415)	60.9	1.00 (0.99–1.01)	44,605 (20,325)	58.2	1.01 (0.99–1.02)	11,810 (4,285)	54.3	1.01 (0.98–1.05)	1,005 (355)	50.1	1.08 (0.96–1.21)	2,255 (1,175)	56.1	0.93 (0.87–0.99)
Chronic heart disease	195,385 (103,225)	62.5	0.96 (0.95–0.97)	107,965 (49,800)	59.2	0.97 (0.96–0.99)	30,860 (11,355)	55.5	0.98 (0.95–1.01)	2,535 (910)	51.4	0.98 (0.89–1.08)	3,860 (2,185)	63.3	1.05 (0.99–1.10)
Chronic liver disease	11,090 (5,770)	59.4	1.05 (1.02–1.08)	5,290 (2,410)	56.3	1.02 (0.98–1.06)	1,505 (585)	55.0	1.06 (0.98–1.16)	240 (85)	44.4	1.18 (0.94–1.47)	585 (340)	60.9	1.22 (1.09–1.36)
Asplenia	3,650 (2,075)	64.5	1.09 (1.04–1.14)	1,575 (795)	61.1	1.10 (1.02–1.18)	455 (180)	55.5	1.08 (0.93–1.25)	80 (35)	50.0	1.51 (1.07–2.14)	170 (90)	56.2	0.98 (0.80–1.20)
Cancer (non-haematologic)	77,375 (42,645)	65.7	1.07 (1.06–1.09)	38,315 (18,430)	62.4	1.07 (1.05–1.09)	11,020 (4,295)	59.0	1.08 (1.05–1.12)	805 (320)	54.7	1.05 (0.93–1.19)	960 (600)	70.7	1.16 (1.07–1.26)
Haematologic cancer	11,885 (6,460)	64.3	1.33 (1.29–1.36)	5,705 (2,895)	64.8	1.33 (1.27–1.39)	1,845 (765)	61.5	1.32 (1.20–1.44)	310 (135)	58.6	1.70 (1.40–2.06)	240 (145)	63.6	1.06 (0.90–1.25)
Organ transplant (non-kidney)	1,195 (700)	62.4	2.57 (2.38–2.77)	685 (365)	60.8	2.26 (2.03–2.52)	215 (110)	57.2	1.97 (1.62–2.40)	75 (35)	42.9	1.72 (1.23–2.41)	[N]	[N]	[N]
CND (inc. learning disability)	77,535 (35,190)	57.6	0.87 (0.86–0.88)	41,125 (16,030)	54.3	0.87 (0.85–0.88)	11,295 (3,520)	51.6	0.90 (0.87–0.94)	765 (235)	44.6	0.87 (0.76–1.01)	1,185 (645)	60.1	1.01 (0.93–1.09)
Severe mental illness	5,405 (1,945)	43.8	0.70 (0.67–0.74)	2,700 (895)	43.6	0.74 (0.70–0.79)	835 (250)	44.9	0.85 (0.75–0.96)	80 (20)	28.6	0.71 (0.46–1.09)	170 (85)	50.0	0.99 (0.79–1.22)
CEV (other)	2,370 (1,280)	61.8	1.09 (1.03–1.15)	1,170 (575)	60.5	1.15 (1.06–1.25)	450 (190)	55.3	1.10 (0.96–1.27)	[N]	[N]	[N]	[N]	[N]	[N]

Individuals were included in the analysis if they were ≥75 years of age at baseline, care home residents, transplant recipients, or had a history of haematologic malignancy or immunosuppression. Counts are rounded to the nearest 5. Cumulative coverage was determined based on Kaplan-Meier estimates, censoring on death, deregistration, or 11th May 2022. Hazard ratios and 95% confidence intervals were derived from partially adjusted models that included age, care home residence, health and social care worker status, housebound status, receipt of end-of-life care, setting (urban/rural), sex, ethnicity, IMD quintile, prior SARS-CoV-2 infection, immunosuppression, and haematologic cancer. Data are redacted [R] for any rows in which there were >0 and ≤10 event or non-event counts after rounding to the nearest 5, with non-event counts calculated among uncensored individuals. CEV, clinically extremely vulnerable; CKD, chronic kidney disease; CND, chronic neurological disease; IMD, index of multiple deprivation; [N], covariate absent in all individuals by definition; [R] redacted; RRT, renal replacement therapy.

For peer review only - http://bmjopen.bmj.com/site/about/guidelines.xhtml

STROBE Statement—checklist of items that should be included in reports of observational studies

	Item No.	Recommendation	Page No.	Relevant text from manuscript
**Title and abstract**	1	(*a*) Indicate the study's design with a commonly used term in the title or the abstract	1–2	"cohort study" referred to in title and abstract
		(*b*) Provide in the abstract an informative and balanced summary of what was done and what was found	2	N/A
**Introduction**				
Background/rationale	2	Explain the scientific background and rationale for the investigation being reported	3	N/A
Objectives	3	State specific objectives, including any prespecified hypotheses	3	Knowledge gaps addressed by current study identified in paragraph 3 of Background.
**Methods**				
Study design	4	Present key elements of study design early in the paper	3	Summary of approach provided in paragraph 4 of Background.
Setting	5	Describe the setting, locations, and relevant dates, including periods of recruitment, exposure, follow-up, and data collection	3–4 & Table 1	'Data sources' subheading provides details of databases supporting analyses. 'Study population' subheading defines relevant dates. 'Outcomes' subheading defines relevant exposures. Table 1 provides a breakdown of the study population by location.
Participants	6	(*a*) *Cohort study*—Give the eligibility criteria, and the sources and methods of selection of participants. Describe methods of follow-up<br>*Case-control study*—Give the eligibility criteria, and the sources and methods of case ascertainment and control selection. Give the rationale for the choice of cases and controls<br>*Cross-sectional study*—Give the eligibility criteria, and the sources and methods of selection of participants	4 & Supp Fig 1	Inclusion criteria are defined under 'Study population' subheading and in Supp Fig 1, which provides a flowchart of cohort selection. Databases are defined under the 'Data sources' subheading.
		(*b*) *Cohort study*—For matched studies, give matching criteria and number of exposed and unexposed	N/A	No matching undertaken.

For peer review only - http://bmjopen.bmj.com/site/about/guidelines.xhtml

		Case-control study—For matched studies, give matching criteria and the number of controls per case		
Variables	7	Clearly define all outcomes, exposures, predictors, potential confounders, and effect modifiers. Give diagnostic criteria, if applicable	4–5	Outcomes are defined under 'Outcomes' subheading. All covariates included in models are defined under 'Covariates' subheading. The sequential adjustment process adopted to account for potential confounding is defined in the first paragraph of the 'Statistical analysis' subheading.
Data sources/ measurement	8*	For each variable of interest, give sources of data and details of methods of assessment (measurement). Describe comparability of assessment methods if there is more than one group	3	All variables were defined using the OpenSAFELY-TPP database described under the 'Data sources' subheading.
Bias	9	Describe any efforts to address potential sources of bias	4–5	The sequential adjustment process adopted to account for potential confounding is defined under the first paragraph of the 'Statistical analysis' subheading. Exclusion of individuals with missing demographic data (which is often an indicator of poor data quality in electronic health records) is described in paragraph 1 under the 'Study population' subheading.
Study size	10	Explain how the study size was arrived at	Supp Fig 1	Supp Fig 1 provides a full flow chart of the study selection process
Quantitative variables	11	Explain how quantitative variables were handled in the analyses. If applicable, describe which groupings were chosen and why	4	Definition of chronic kidney disease stage in relation to eGFR followed standard diagnostic thresholds, as described in paragraph 3 of the 'Study population' subheading. Age cut-offs were defined based on vaccine eligibility criteria, as described in paragraph 1 under the 'Covariates' subheading.
Statistical methods	12	(*a*) Describe all statistical methods, including those used to control for confounding	5	The sequential adjustment process adopted to account for potential confounding is defined under

For peer review only - http://bmjopen.bmj.com/site/about/guidelines.xhtml

				the first paragraph of the 'Statistical analysis' subheading.
		(*b*) Describe any methods used to examine subgroups and interactions	5	Subgroup analyses are described in paragraph 2 under the 'Statistical analysis' subheading.
		(*c*) Explain how missing data were addressed	4	Exclusion of individuals with missing demographic data (which is often an indicator of poor data quality in electronic health records) is described in paragraph 1 under the 'Study population' subheading. Data were complete for all other covariates.
		(*d*) *Cohort study*—If applicable, explain how loss to follow-up was addressed<br>*Case-control study*—If applicable, explain how matching of cases and controls was addressed<br>*Cross-sectional study*—If applicable, describe analytical methods taking account of sampling strategy	5	As noted in paragraph 1 under the 'Statistical analysis' subheading, we censored at death, deregistration, or the end of the study period (11th May 2022).
		(*e*) Describe any sensitivity analyses	5	The logistic regression approach used as a sensitivity analysis is described in paragraph 2 under this subheading.
**Results**				
Participants	13*	(a) Report numbers of individuals at each stage of study—eg numbers potentially eligible, examined for eligibility, confirmed eligible, included in the study, completing follow-up, and analysed	Supp Fig 1 and Supp Table 2	Supp Fig 1 provides a full flow chart of the study selection process. The number of individuals uncensored at the end of follow-up is defined in Supp Table 2 (penultimate column).
		(b) Give reasons for non-participation at each stage	N/A	N/A
		(c) Consider use of a flow diagram	Supp Fig 1	Supp Fig 1 provides a full flow chart of the study selection process.
Descriptive data	14*	(a) Give characteristics of study participants (eg demographic, clinical, social) and information on exposures and potential confounders	Table 1 and Supp Table 4	Table 1 and Supp Table 4 provide a summary of the covariate profile for the study population as a whole and analysis subgroups.
		(b) Indicate number of participants with missing data for each variable of interest	N/A	Data were complete for all covariates in the final study population.

For peer review only - http://bmjopen.bmj.com/site/about/guidelines.xhtml
For peer review only

		(c) *Cohort study*—Summarise follow-up time (eg, average and total amount)	Fig 1	Fig 1A provides a summary of the event distribution for each outcome over the course of follow-up. The analysis cut-off (11th May 2022) is specified in the figure legend and elsewhere in the manuscript.
Outcome data	15*	*Cohort study*—Report numbers of outcome events or summary measures over time	Supp Tables 2, 3, 5, and 6	Supp Tables provide a breakdown of the number of events by subgroup for all analyses
		Case-control study—Report numbers in each exposure category, or summary measures of exposure	N/A	N/A
		Cross-sectional study—Report numbers of outcome events or summary measures	N/A	N/A
Main results	16	(*a*) Give unadjusted estimates and, if applicable, confounder-adjusted estimates and their precision (eg, 95% confidence interval). Make clear which confounders were adjusted for and why they were included	Supp Tables 2 and 5	Supp Tables 2 and 5 present results of sequentially adjusted models with 95% CIs. The variables included in the models are indicated via the symbols * (minimally adjusted models) and † (partially adjusted models)
		(*b*) Report category boundaries when continuous variables were categorized	Table 1 and Supp Tables 2–6	The boundaries used to define age groups are specified in all results tables. No other covariates required category boundaries to be specified.
		(*c*) If relevant, consider translating estimates of relative risk into absolute risk for a meaningful time period	N/A	N/A
Other analyses	17	Report other analyses done—eg analyses of subgroups and interactions, and sensitivity analyses	Supp Tables 2, 3, and 6	Subgroup analyses are summarised in Supp Tables 3 and 6. Sensitivity analyses (via logistic regression) are summarised in Supp Table 2.
**Discussion**				
Key results	18	Summarise key results with reference to study objectives	7	'Summary' subheading
Limitations	19	Discuss limitations of the study, taking into account sources of potential bias or imprecision. Discuss both direction and magnitude of any potential bias	8	'Strengths and limitations' subheading

For peer review only - http://bmjopen.bmj.com/site/about/guidelines.xhtml

Interpretation	20	Give a cautious overall interpretation of results considering objectives, limitations, multiplicity of analyses, results from similar studies, and other relevant evidence	7–8	'Findings in context' subheading
Generalisability	21	Discuss the generalisability (external validity) of the study results	9	'Policy implications and interpretation' subheading
**Other information**				
Funding	22	Give the source of funding and the role of the funders for the present study and, if applicable, for the original study on which the present article is based	9	'Funding' subheading

*Give information separately for cases and controls in case-control studies and, if applicable, for exposed and unexposed groups in cohort and cross-sectional studies.

Note: An Explanation and Elaboration article discusses each checklist item and gives methodological background and published examples of transparent reporting. The STROBE checklist is best used in conjunction with this article (freely available on the Web sites of PLoS Medicine at http://www.plosmedicine.org/, Annals of Internal Medicine at http://www.annals.org/, and Epidemiology at http://www.epidem.com/). Information on the STROBE Initiative is available at www.strobe-statement.org.

For peer review only

BMJ Open

Factors associated with COVID-19 vaccine uptake in people with kidney disease: an OpenSAFELY cohort study

Journal:	*BMJ Open*
Manuscript ID	bmjopen-2022-066164.R1
Article Type:	Original research
Date Submitted by the Author:	21-Dec-2022
Complete List of Authors:	Parker, Edward PK; London School of Hygiene & Tropical Medicine<br>Tazare, John; London School of Hygiene & Tropical Medicine<br>Hulme, William J; Oxford University<br>Bates, Christopher; TPP<br>Carr, Edward J; The Francis Crick Institute<br>Cockburn, Jonathan; TPP<br>Curtis, Helen; Oxford University<br>Fisher, Louis; Oxford University<br>Green, Amelia CA; Oxford University<br>Harper, Sam; TPP<br>Hester, Frank; TPP<br>Horne, Elsie MF; University of Bristol<br>Loud, Fiona; Kidney Care UK<br>Lyon, Susan; UK Kidney Association<br>Mahalingasivam, Viyaasan; London School of Hygiene & Tropical Medicine<br>Mehrkar, Amir; Oxford University<br>Nab, Linda; Oxford University<br>Parry, John; TPP<br>Santhakumaran, Shalini; UK Renal Registry<br>Steenkamp, Retha; UK Renal Registry<br>Sterne, Jonathan; University of Bristol<br>Walker, Alex; Oxford University<br>Williamson, Elizabeth; London School of Hygiene & Tropical Medicine<br>Willicombe, Michelle; Imperial College London<br>Zheng, Bang; London School of Hygiene & Tropical Medicine<br>Goldacre, Ben; Oxford University<br>Nitsch, Dorothea; London School of Hygiene & Tropical Medicine<br>Tomlinson, Laurie; London School of Hygiene & Tropical Medicine
**Primary Subject Heading**:	Public health
Secondary Subject Heading:	Infectious diseases
Keywords:	COVID-19, Kidney & urinary tract disorders < UROLOGY, Public health < INFECTIOUS DISEASES

For peer review only - http://bmjopen.bmj.com/site/about/guidelines.xhtml

SCHOLARONE™
Manuscripts

I, the Submitting Author has the right to grant and does grant on behalf of all authors of the Work (as defined in the below author licence), an exclusive licence and/or a non-exclusive licence for contributions from authors who are: i) UK Crown employees; ii) where BMJ has agreed a CC-BY licence shall apply, and/or iii) in accordance with the terms applicable for US Federal Government officers or employees acting as part of their official duties; on a worldwide, perpetual, irrevocable, royalty-free basis to BMJ Publishing Group Ltd ("BMJ") its licensees and where the relevant Journal is co-owned by BMJ to the co-owners of the Journal, to publish the Work in this journal and any other BMJ products and to exploit all rights, as set out in our licence.

The Submitting Author accepts and understands that any supply made under these terms is made by BMJ to the Submitting Author unless you are acting as an employee on behalf of your employer or a postgraduate student of an affiliated institution which is paying any applicable article publishing charge ("APC") for Open Access articles. Where the Submitting Author wishes to make the Work available on an Open Access basis (and intends to pay the relevant APC), the terms of reuse of such Open Access shall be governed by a Creative Commons licence – details of these licences and which Creative Commons licence will apply to this Work are set out in our licence referred to above.

Other than as permitted in any relevant BMJ Author's Self Archiving Policies, I confirm this Work has not been accepted for publication elsewhere, is not being considered for publication elsewhere and does not duplicate material already published. I confirm all authors consent to publication of this Work and authorise the granting of this licence.

For peer review only

Factors associated with COVID-19 vaccine uptake in people with kidney disease: an OpenSAFELY cohort study

The OpenSAFELY Collaborative: Edward PK Parker[1], John Tazare[1], William J Hulme[2], Christopher Bates[3], Edward J Carr[4], Jonathan Cockburn[3], Helen J Curtis[2], Louis Fisher[2], Amelia CA Green[2], Sam Harper[3], Frank Hester[3], Elsie MF Horne[5,6], Fiona Loud[7], Susan Lyon[8], Viyaasan Mahalingasivam[1], Amir Mehrkar[2], Linda Nab[2], John Parry[3], Shalini Santhakumaran[9], Retha Steenkamp[9], Jonathan AC Sterne[5,6,10], Alex J Walker[2], Elizabeth J Williamson[1], Michelle Willicombe[11,12], Bang Zheng[1], Ben Goldacre[2], Dorothea Nitsch[1,9], Laurie A Tomlinson[1]

[1] London School of Hygiene and Tropical Medicine, Keppel Street, London, WC1E 7HT, UK
[2] Bennett Institute for Applied Data Science, Nuffield Department of Primary Care Health Sciences, University of Oxford, OX2 6GG, UK
[3] TPP, TPP House, 129 Low Lane, Horsforth, Leeds, LS18 5PX, UK
[4] The Francis Crick Institute, London, NW1 1AT, UK
[5] Population Health Sciences, University of Bristol, Oakfield House, Oakfield Grove, Bristol, BS8 2BN, UK
[6] NIHR Bristol Biomedical Research Centre, Bristol, UK
[7] Kidney Care UK, Alton, UK
[8] Patient Council, UK Kidney Association, Bristol, UK
[9] UK Renal Registry, Bristol, UK
[10] Health Data Research UK South-West, Bristol, UK
[11] Centre for Inflammatory Disease, Department of Immunology and Inflammation, Imperial College London, Hammersmith Campus, Du Cane Road London, W12 0NN, UK
[12] Imperial College Renal and Transplant Centre, Imperial College Healthcare NHS Trust, Hammersmith Hospital, London, W12 0HS, UK

Correspondence to: Laurie Tomlinson, London School of Hygiene and Tropical Medicine, Keppel Street, London, WC1E 7HT, UK; Email: laurie.tomlinson@lshtm.ac.uk

For peer review only - http://bmjopen.bmj.com/site/about/guidelines.xhtml

Abstract

Objective: To characterise factors associated with COVID-19 vaccine uptake among people with kidney disease in England.

Design: Retrospective cohort study using the OpenSAFELY-TPP platform, performed with the approval of NHS England.

Setting: Individual-level routine clinical data from 24 million people across GPs in England using TPP software. Primary care data were linked directly with COVID-19 vaccine records up to 31st August 2022 and with renal replacement therapy (RRT) status via the UK Renal Registry.

Participants: A cohort of adults with stage 3–5 chronic kidney disease (CKD) or receiving RRT at the start of the COVID-19 vaccine roll-out were identified based on evidence of reduced estimated glomerular filtration rate (eGFR) or inclusion in the UK Renal Registry.

Main outcome measures: Dose-specific vaccine coverage over time was determined from 1st December 2020 to 31st August 2022. Individual-level factors associated with receipt of a 3- or 4-dose vaccine series were explored via Cox proportional hazards models.

Results: 992,205 people with stage 3–5 CKD or receiving RRT were included. Cumulative vaccine coverage as of 31st August 2022 was 97.5%, 97.0%, and 93.9% for doses 1, 2, and 3, respectively, and 81.9% for dose 4 among individuals with one or more indications for eligibility. Delayed 3-dose vaccine uptake was associated with younger age, minority ethnicity, social deprivation, and severe mental illness – associations that were consistent across CKD severity subgroups, dialysis patients, and kidney transplant recipients. Similar associations were observed for 4-dose uptake.

Conclusion: Although high primary vaccine and booster dose coverage has been achieved among people with kidney disease in England, key disparities in vaccine uptake remain across clinical and demographic groups and 4-dose coverage is suboptimal. Targeted interventions are needed to identify barriers to vaccine uptake among under-vaccinated subgroups identified in the present study.

Key words: COVID-19; chronic kidney disease; NHS England; SARS-CoV-2; vaccination; vaccine coverage.

For peer review only - http://bmjopen.bmj.com/site/about/guidelines.xhtml

Strengths and limitations of this study

- We harnessed unique electronic health data linkages to provide a comprehensive overview of demographic and clinical factors associated with COVID-19 vaccine coverage in a large cohort (*n* = 992,205) of people with moderate-to-severe kidney disease.
- We used a gold-standard registry of people receiving treatment for end-stage kidney disease (the UK Renal Registry) to identify dialysis and transplant recipients at the start of the UK COVID-19 vaccine roll-out.
- We estimated cumulative 3- and 4-dose coverage estimates in subgroups defined by ethnicity, social deprivation status, age, and kidney disease severity to identify under-vaccinated populations.
- The under-representation of certain regions (such as London) in the OpenSAFELY-TPP database may limit generalisability of our findings.
- Eligibility for a fourth COVID-19 vaccine dose could not be precisely determined based on diagnosis and prescription codes in electronic health records.

For peer review only

Background

Since the emergence of SARS-CoV-2, individuals with kidney disease – in particular those requiring renal replacement therapy (RRT) – have been at particular risk of enduring the most harmful effects of COVID-19[1 2]. While vaccination has the potential to mitigate this risk, people with kidney disease exhibit impaired COVID-19 vaccine immunogenicity and effectiveness compared to healthy adults[3 4]. Among individuals who have received a 2-dose primary vaccine series, chronic kidney disease (CKD) and receipt of RRT have been identified as key risk factors for subsequent infection, COVID-19-related hospitalisation, and COVID-19-related mortality[5].

Approximately 2.6 million individuals over 16 years of age are estimated to be affected by stage 3–5 CKD in England (prevalence of ~6%)[6]. Given their continued risk of COVID-19 after primary vaccination, people with kidney disease have been prioritised for additional vaccine doses. The UK Joint Committee on Vaccination and Immunisation (JCVI) recommended that individuals with substantial immunosuppression (including primary or acquired immunodeficiency and recent or ongoing immunosuppressive therapy[7]) receive a third primary vaccine dose from 1st September 2021. A subset of individuals with kidney disease, such as those receiving immunosuppressive therapy following kidney transplant, would have been eligible for this third primary dose. Subsequently, all individuals with CKD were included in the initial JCVI priority groups to be offered a booster dose from 14th September 2021[8], with eligibility later extending to all adults as of 29th November 2021[9].

Within the UK's guidelines there were two routes to being offered a fourth dose of COVID-19 vaccine before September 2022. Firstly, severely immunosuppressed individuals who received a third primary dose would become eligible for a booster (fourth) dose at an interval of 3 months, and a second booster (fifth) dose at least 3 months later. Secondly, from February 2022[10], individuals who were >75 years of age, care home residents, or immunosuppressed become eligible for a fourth dose as a 2-dose primary course followed by autumn 2021 and spring 2022 boosters. As CKD incidence climbs with age, the majority of people with kidney disease in the UK became eligible for a fourth dose via this second route.

Global and national variation in COVID-19 vaccine uptake is monitored via several online dashboards[11-13]. In England, relative vaccine uptake among demographic and clinical subgroups, including people with CKD, is reported in weekly coverage reports using the OpenSAFELY platform[13 14]. However, in these summary reports it remains unclear whether COVID-19 vaccine coverage varies according to kidney disease severity or in relation to other demographic or clinical factors in this high-risk group.

To address these issues, we conducted a retrospective cohort study within the OpenSAFELY-TPP database (https://opensafely.org). We report on the status of COVID-19 vaccination among people with kidney disease up to 31st August 2022 and highlight individual-level characteristics associated with vaccine uptake.

Methods

For peer review only - http://bmjopen.bmj.com/site/about/guidelines.xhtml

Data sources

All data were linked, stored, and analysed securely within the OpenSAFELY platform https://opensafely.org/. OpenSAFELY is a data analytics platform created by our team on behalf of NHS England to address urgent COVID-19 research questions. The dataset analysed within OpenSAFELY-TPP is based on 24 million people currently registered with GP surgeries using TPP SystmOne software. Data include pseudonymised data such as coded diagnoses, medications, and physiological parameters. No free text data are included. All code is shared openly for review and re-use under MIT open license (https://github.com/opensafely/ckd-coverage-ve). Detailed pseudonymised patient data is potentially re-identifiable and therefore not shared. Primary care data are linked through OpenSAFELY with other pseudonymised datasets, including COVID-19 testing records via the Second Generation Surveillance System (SGSS), A&E attendance and hospital records via NHS Digital's Hospital Episode Statistics (HES), national death registry records from the Office for National Statistics (ONS), and RRT status via the UK Renal Registry (UKRR). Vaccination status is directly available within GP records via the National Immunisation Management System (NIMS).

Study population

Baseline clinical and demographic characteristics were defined as of 1st December 2020 – the month in which the UK COVID-19 vaccination programme commenced – with the exception of age, which was calculated as of 31st March 2021 as per UK Health Security Agency recommendations[15]. We assessed eligibility for inclusion in the study cohort among individuals who were at least 16 years of age and had been registered in OpenSAFELY-TPP for at least 3 months at baseline. We excluded individuals if their sex, ethnicity, geographical region, or index of multiple deprivation (IMD) were unknown, or if they had an atypical vaccine record (more than five doses recorded by 31st August 2022 or any two doses recorded within a period of 14 days).

We determined kidney function based on the most recent serum creatinine measurement in the 2 years preceding 1st December 2020 (where available) and converted this into estimated glomerular filtration rate (eGFR) using the CKD epidemiology collaboration (CKD-EPI) equation without specification of ethnicity[16]. Individuals receiving RRT were identified based on their treatment modality (dialysis or transplant) within the UKRR as of 31st December 2020 (the UKRR status update closest to the baseline date).

We included individuals who were receiving RRT as well as people with CKD based on evidence of reduced kidney function (eGFR <60 ml/min/1.73 m^2) in the absence of RRT. Individuals with primary care codes suggesting prior receipt of dialysis or kidney transplant but absent from the UKRR cohort were excluded. To assess CKD severity, we distinguished between stage 3a (eGFR of 45–59 ml/min/1.73 m^2), stage 3b (eGFR of 30–44 ml/min/1.73 m^2), and stage 4–5 CKD (eGFR <30 ml/min/1.73 m^2).

Outcomes

We report on cumulative coverage for doses 1, 2, 3, 4, and 5 (via any combination of vaccine products) up to 31st August 2022. For the analysis of individual-level factors associated with

For peer review only

For peer review only - http://bmjopen.bmj.com/site/about/guidelines.xhtml

vaccine uptake, our primary outcome was receipt of a 3-dose series of any vaccine product. For the purposes of this study, we do not distinguish between third primary and booster doses, or between 100 µg (full) or 50 µg (half) doses of the vaccine product mRNA-1273 (with the latter recommended for booster doses).

Receipt of a 4-dose series of any vaccine product was considered as a secondary outcome. Notably, not all members of our cohort would have been eligible for a fourth dose before September 2022 based on UK guidelines (described above). We therefore limited our secondary outcome analysis to a subset of the population with at least one indicator for receipt of a fourth dose (3 primary + 1 booster or 2 primary + 2 boosters), including individuals who were ≥75 years of age at baseline, care home residents, transplant recipients, or had a history of haematologic malignancy or immunosuppression.

Covariates

Covariates of interest in relation to demography, vaccine prioritisation, and population accessibility included: age (five categories defined by JCVI priority group cut-offs); sex; ethnicity (White, Black or Black British, Asian or Asian British, Mixed, or other); social deprivation based on IMD quintile (derived from an individual's postcode at Lower Super Output Area); setting (urban, urban conurbation, or rural); care home residence; health and social care worker status; housebound status; and receipt of end-of-life care.

Clinical covariates of interest included kidney disease subgroup (CKD3a, CKD3b, CKD4–5, RRT [dialysis], and RRT [transplant]) and presence or absence of pre-existing conditions including: prior SARS-CoV-2 infection; immunosuppression (defined by any prior immunosuppressive diagnosis or receipt of immunosuppressive therapy in the 6 months preceding baseline); moderate/severe obesity (body mass index ≥30 kg/m^2 based on the most recent measurement in the 5 years before baseline); diabetes; chronic respiratory disease (including asthma); chronic heart disease; chronic liver disease; asplenia; non-haematologic cancer; haematologic cancer; non-kidney organ transplant; chronic neurological disease (including learning disability); severe mental illness; and classification as clinically extremely vulnerable[17] in the absence of any of the conditions listed above (including RRT). To assess the potential influence of primary care coding relating to kidney disease, we included the presence or absence of diagnostic codes associated with stage 3–5 CKD at baseline.

Statistical analysis

We used Kaplan-Meier estimates to determine cumulative dose-specific vaccine coverage over time, censoring at death, deregistration, or the end of the study period (31st August 2022). Individuals remained in the at-risk population for receiving doses 2–5 regardless of their vaccination status for prior doses. To identify demographic and clinical covariates associated with time to receipt of a 3-dose (primary outcome) or 4-dose (secondary outcome) vaccine series, we used Cox proportional hazards models, stratified by region, and censoring at death, deregistration, or the end of the study period. For each covariate, we determined hazard ratios (HRs) and 95% confidence intervals (CIs) with sequential adjustment for baseline covariates. Minimally adjusted models included age, care home residence, and health and social care worker status given their use in JCVI prioritisation criteria. Partially adjusted models extended these models to include additional variables

For peer review only - http://bmjopen.bmj.com/site/about/guidelines.xhtml

potentially linked with vaccine access or uptake, including housebound status, receipt of end-of-life care, setting, sex, ethnicity, IMD quintile, prior SARS-CoV-2 infection, immunosuppression, and haematologic cancer. Fully adjusted models included all covariates. Schoenfeld residual plots were used to assess validity of the proportional hazards assumption. As a precaution against small-number disclosures, we rounded counts to the nearest 5, redacted non-zero rounded counts or non-counts of ≤10, and delayed Kaplan-Meier steps until ≥5 events had occurred.

To determine whether HRs differed according to kidney disease severity, partially adjusted models were explored in the following subgroups: CKD3a, CKD3b, CKD4–5, RRT (dialysis), and RRT (transplant). To assess whether conclusions were robust to Cox model assumptions and representative of factors associated with vaccine coverage at the time of the analysis cut-off (31st August 2022), we performed a sensitivity analysis for the primary outcome in which we used partially adjusted logistic regression models (using the same variables above but with region as an additional covariate) among individuals who remained alive and registered throughout follow-up. Finally, we calculated cumulative 3- and 4-dose coverage in subgroups defined by ethnicity, IMD quintile, age, and kidney disease severity to identify populations at particular risk of under-vaccination at the end of follow-up. While recognising their heterogeneity, ethnic minority groups are combined in this subgroup analysis due to their low combined prevalence (<10%) within the study population.

Patient and public involvement

Patients were not directly involved in developing this study. We maintain a publicly available website (https://opensafely.org) through which members of the public or patient groups are invited to contact us regarding this study or the broader OpenSAFELY project.

Results

Vaccine coverage and product profile by dose

A total of 992,205 individuals met the criteria for inclusion in the cohort (**Supplementary Figure 1**). Of these, 66.5% had CKD3a, 24.7% had CKD3b, 6.5% had CKD4–5, 1.0% were receiving RRT via dialysis, and 1.3% were receiving RRT via kidney transplant. Clinical and demographic characteristics of the overall cohort and kidney disease subgroups are summarised in **Table 1**. In comparison to people with CKD, RRT recipients were markedly younger (with associated declines in several age-associated comorbidities) and were more likely to be male and of minority ethnicity.

For peer review only - http://bmjopen.bmj.com/site/about/guidelines.xhtml

Table 1. Baseline characteristics of study population.

Characteristic	All, n (%)	CKD3a, n (%)	CKD3b, n (%)	CKD4–5, n (%)	RRT (dialysis), n (%)	RRT (transplant), n (%)
**N**	992,205 (100)	659,785 (100)	245,380 (100)	64,855 (100)	9,455 (100)	12,735 (100)
**Age**						
16–64	109,815 (11.1)	76,390 (11.6)	13,750 (5.6)	5,990 (9.2)	4,395 (46.5)	9,285 (72.9)
65–69	74,740 (7.5)	57,870 (8.8)	11,270 (4.6)	3,090 (4.8)	1,095 (11.6)	1,415 (11.1)
70–74	143,575 (14.5)	109,375 (16.6)	25,860 (10.5)	6,025 (9.3)	1,145 (12.1)	1,170 (9.2)
75–79	181,200 (18.3)	130,805 (19.8)	39,940 (16.3)	8,620 (13.3)	1,225 (13.0)	615 (4.8)
80+	482,870 (48.7)	285,345 (43.2)	154,560 (63.0)	41,135 (63.4)	1,590 (16.8)	245 (1.9)
**Sex**						
Female	555,365 (56.0)	373,790 (56.7)	139,545 (56.9)	33,635 (51.9)	3,490 (36.9)	4,910 (38.6)
Male	436,840 (44.0)	285,995 (43.3)	105,835 (43.1)	31,220 (48.1)	5,965 (63.1)	7,825 (61.4)
**Ethnicity**						
White	930,565 (93.8)	621,555 (94.2)	231,735 (94.4)	59,610 (91.9)	7,305 (77.3)	10,355 (81.3)
Mixed	17,675 (1.8)	11,895 (1.8)	3,440 (1.4)	1,270 (2.0)	575 (6.1)	490 (3.8)
Asian or Asian British	32,620 (3.3)	19,015 (2.9)	7,720 (3.1)	3,115 (4.8)	1,280 (13.5)	1,490 (11.7)
Black or Black British	4,755 (0.5)	3,195 (0.5)	975 (0.4)	340 (0.5)	120 (1.3)	125 (1.0)
Other ethnic groups	6,590 (0.7)	4,125 (0.6)	1,505 (0.6)	515 (0.8)	170 (1.8)	275 (2.2)
**Index of multiple deprivation quintile**						
1 most deprived	164,415 (16.6)	104,895 (15.9)	42,025 (17.1)	12,265 (18.9)	2,625 (27.8)	2,605 (20.5)
2	187,240 (18.9)	122,190 (18.5)	47,225 (19.2)	12,990 (20.0)	2,185 (23.1)	2,650 (20.8)
3	224,590 (22.6)	149,385 (22.6)	55,965 (22.8)	14,600 (22.5)	1,890 (20.0)	2,750 (21.6)
4	216,980 (21.9)	146,780 (22.2)	52,795 (21.5)	13,355 (20.6)	1,530 (16.2)	2,515 (19.7)
5 least deprived	198,985 (20.1)	136,535 (20.7)	47,370 (19.3)	11,640 (17.9)	1,225 (13.0)	2,210 (17.4)
**Setting**						
Urban city or town	530,975 (53.5)	353,115 (53.5)	131,920 (53.8)	34,580 (53.3)	4,750 (50.2)	6,605 (51.9)
Urban conurbation	204,530 (20.6)	132,680 (20.1)	50,665 (20.6)	14,715 (22.7)	2,995 (31.7)	3,470 (27.2)
Rural	256,700 (25.9)	173,990 (26.4)	62,790 (25.6)	15,560 (24.0)	1,705 (18.0)	2,655 (20.8)
**Primary care coding of kidney disease**						
CKD3–5 diagnostic code	592,520 (59.7)	326,090 (49.4)	191,500 (78.0)	57,250 (88.3)	8,030 (84.9)	9,650 (75.8)
Dialysis code	15,785 (1.6)	[N]	[N]	[N]	7,485 (79.2)	8,300 (65.2)
Kidney transplant code	13,630 (1.4)	[N]	[N]	[N]	1,290 (13.6)	12,340 (96.9)
**Risk group (occupation/access)**						
Care home resident	44,470 (4.5)	24,280 (3.7)	15,255 (6.2)	4,705 (7.3)	165 (1.7)	65 (0.5)
Health/social care worker	5,680 (0.6)	4,395 (0.7)	630 (0.3)	210 (0.3)	70 (0.7)	370 (2.9)
Housebound	54,600 (5.5)	27,320 (4.1)	19,140 (7.8)	6,930 (10.7)	600 (6.3)	610 (4.8)

For peer review only - http://bmjopen.bmj.com/site/about/guidelines.xhtml

End of life care	40,400 (4.1)	20,555 (3.1)	13,545 (5.5)	5,515 (8.5)	605 (6.4)	180 (1.4)
**Risk group (clinical)**						
Prior SARS-CoV-2 *	27,835 (2.8)	15,570 (2.4)	7,830 (3.2)	2,855 (4.4)	1,045 (11.1)	535 (4.2)
Immunosuppression	56,985 (5.7)	33,045 (5.0)	13,600 (5.5)	4,000 (6.2)	985 (10.4)	5,355 (42.0)
Moderate/severe obesity	111,630 (11.3)	73,530 (11.1)	27,730 (11.3)	7,910 (12.2)	1,315 (13.9)	1,150 (9.0)
Diabetes	275,745 (27.8)	156,995 (23.8)	82,940 (33.8)	28,015 (43.2)	3,950 (41.8)	3,840 (30.2)
Chronic respiratory disease (inc. asthma)	120,935 (12.2)	75,960 (11.5)	33,880 (13.8)	9,085 (14.0)	1,015 (10.7)	990 (7.8)
Chronic heart disease	438,965 (44.2)	264,765 (40.1)	128,885 (52.5)	36,710 (56.6)	4,755 (50.3)	3,850 (30.2)
Chronic liver disease	32,215 (3.2)	20,940 (3.2)	7,850 (3.2)	2,290 (3.5)	550 (5.8)	585 (4.6)
Asplenia	8,665 (0.9)	5,680 (0.9)	2,090 (0.9)	585 (0.9)	140 (1.5)	170 (1.3)
Cancer (non-haematologic)	168,840 (17.0)	107,245 (16.3)	46,265 (18.9)	13,055 (20.1)	1,315 (13.9)	960 (7.5)
Haematologic cancer	20,430 (2.1)	12,180 (1.8)	5,830 (2.4)	1,875 (2.9)	310 (3.3)	240 (1.9)
Organ transplant (non-kidney) †	2,190 (0.2)	1,205 (0.2)	690 (0.3)	220 (0.3)	75 (0.8)	[N]
CND (inc. learning disability)	163,135 (16.4)	99,640 (15.1)	47,650 (19.4)	13,215 (20.4)	1,450 (15.3)	1,180 (9.3)
Severe mental illness	12,985 (1.3)	8,375 (1.3)	3,275 (1.3)	1,035 (1.6)	180 (1.9)	125 (1.0)
Clinically extremely vulnerable (other) §	9,065 (0.9)	5,765 (0.9)	2,220 (0.9)	1,075 (1.7)	[N]	[N]
**Region**						
East of England	220,630 (22.2)	147,850 (22.4)	53,895 (22.0)	13,835 (21.3)	2,085 (22.1)	2,970 (23.3)
Midlands	227,805 (23.0)	152,235 (23.1)	55,885 (22.8)	14,685 (22.6)	2,280 (24.1)	2,715 (21.3)
London	30,610 (3.1)	19,080 (2.9)	7,315 (3.0)	2,545 (3.9)	765 (8.1)	905 (7.1)
North East and Yorkshire	187,440 (18.9)	123,370 (18.7)	47,135 (19.2)	12,615 (19.5)	1,855 (19.6)	2,465 (19.4)
North West	90,180 (9.1)	59,455 (9.0)	22,825 (9.3)	6,050 (9.3)	685 (7.2)	1,160 (9.1)
South East	58,810 (5.9)	39,085 (5.9)	14,580 (5.9)	3,830 (5.9)	580 (6.1)	735 (5.8)
South West	176,735 (17.8)	118,715 (18.0)	43,745 (17.8)	11,295 (17.4)	1,205 (12.7)	1,780 (14.0)

Data are n (%) after rounding to the nearest 5. Primary care codes and risk groups are coded by separate binary variables; percentages under these table subheadings therefore do not sum to 100. Among people with CKD4–5, 59,480 had stage 4 CKD (eGFR 15–29 ml/min/1.73 m^2) and 5,375 had stage 5 CKD (eGFR <15ml/min/1.73 m^2). CKD, chronic kidney disease; CND, chronic neurological disease; [N], covariate absent in all individuals by definition; RRT, renal replacement therapy. * Based on prior evidence of a positive SARS-CoV-2 test, COVID-19-related primary care code, or COVID-19-related hospitalisation as of 1st December 2020; † Excludes individuals with kidney transplants based on primary care coding or UK Renal Registry status; § Classified as clinically extremely vulnerable in the absence of any of the comorbidities listed above (including RRT).

For peer review only

For peer review only - http://bmjopen.bmj.com/site/about/guidelines.xhtml

Cumulative coverage in people with kidney disease as of 31st August 2022 was 97.5% for dose 1, 97.0% for dose 2, and 93.9% for dose 3 (**Figure 1**). Dose 4 coverage was 61.4% for the cohort as a whole and 81.9% among the subset of individuals with at least one indicator for receipt of a third primary or second booster dose. Only 19,255 individuals had received a fifth dose, corresponding to a cumulative coverage of 2.3% in the population as a whole.

For their primary vaccine schedule (doses 1 and 2), most individuals received a homologous series of BNT162b2 (53.4% of recorded schedules) or ChAdOx1-S (45.8%), with <1% receiving either heterologous schedules or mRNA-1273 (**Supplementary Table 1**). For subsequent doses, individuals primarily received BNT162b2 (91.8% and 40.4% of recorded doses 3 and 4, respectively) or mRNA-1273 (7.9% and 59.6% of recorded doses 3 and 4, respectively), reflecting JCVI recommendations that RNA vaccines be preferentially used for third primary or booster doses[7 8]. Accordingly, heterologous third doses were prevalent, primarily involving the administration of BNT162b2 or mRNA-1273 to individuals who had received two prior doses of ChAdOx1-S (39.7% and 5.2% of recorded 3-dose schedules, respectively), though homologous vaccination with BNT162b2 remained the most common 3-dose schedule overall (51.7%; **Supplementary Table 1**).

Factors associated with 3-dose vaccine uptake

Associations between individual-level factors and time to receipt of a 3-dose series are shown in **Figure 2** and **Supplementary Table 2**. HRs were generally stable across minimally, partially, and fully adjusted models (**Supplementary Table 2**). To mitigate potential overadjustment bias, we report on partially adjusted models hereafter.

Vaccine uptake was faster in older individuals and in health and social care workers, reflecting the inclusion of these groups in JCVI prioritisation criteria[17]. By contrast, 3-dose uptake was slower among individuals living in more deprived areas (HR [95% CI] of 1.44 [1.43–1.45] for comparison of least deprived with most deprived IMD quintile), among ethnic minority groups (HRs of 0.48–0.70 relative to White individuals), and individuals who were housebound (HR 0.61 [0.61–0.62]) or receiving end-of-life care (HR 0.87 [0.86–0.88]). These associations were highly consistent across kidney disease subgroups, including RRT recipients (**Supplementary Figure 2** and **Supplementary Table 3**).

In comparison to people with CKD3a, 3-dose vaccine uptake was somewhat slower among people with CKD3b (HR 0.94 [0.93–0.94]) and CKD4–5 (0.87 [0.87–0.88]), but faster among individuals receiving RRT (HRs of 1.11 [1.08–1.14] and 1.51 [1.48–1.54] for dialysis and transplant recipients, respectively). Having a primary care code associated with stage 3–5 CKD was not strongly associated with vaccine uptake in the cohort as a whole (HR 1.04 [1.04–1.04]), but was associated with faster vaccine uptake in more severe kidney disease subgroups (HRs of <1.1 for CKD3a and CKD3b, 1.17 [1.13–1.20] for CKD4–5, 1.14 [1.07–1.22] for dialysis recipients, and 1.11 [1.06–1.16] for transplant recipients; **Supplementary Figure 2**).

Other clinical covariates tended to have modest associations with vaccine uptake (HRs of 0.9–1.1). However, immunosuppression (HR 1.19 [1.18–1.20]) and receipt of non-kidney organ transplants (HR 1.36 [1.30–1.43]) were associated with faster 3-dose uptake, while severe mental illness was associated with slower uptake (HR 0.78 [0.76–0.80]). These

For peer review only - http://bmjopen.bmj.com/site/about/guidelines.xhtml

associations were highly consistent across CKD and RRT subgroups (**Supplementary Figure 2**).

Cumulative coverage estimates stratified by ethnicity and IMD quintile revealed population subgroups with particularly low vaccination rates (**Figure 3** and **Supplementary Table 4**). Among ethnic minority groups, 3-dose coverage ranged from 67% in the lowest IMD quintile to 88% in the highest, and was <90% across age and kidney disease subgroups (**Supplementary Table 4**). Among White individuals, 3-dose coverage fell below 90% among individuals <70 years of age and RRT recipients in the most deprived IMD quintiles.

Schoenfeld residual plots revealed no major violations of the proportional hazards assumption. Associations were generally consistent when explored via logistic regression among 838,975 individuals who were uncensored throughout follow-up (**Supplementary Table 2**), with the exception that having a clinical code associated with stage 3–5 CKD was positively correlated with vaccine uptake in the overall cohort using this approach (odds ratio 1.25 [1.23–1.28]).

Factors associated with 4-dose vaccine uptake

A total of 698,755 individuals (70.4% of the overall cohort) had at least one indicator for receipt of a fourth dose (3 primary + 1 booster or 2 primary + 2 boosters) and were included in the secondary outcome analysis. Baseline clinical and demographic characteristics for these individuals are summarised in **Supplementary Table 5**.

Factors associated with 4-dose uptake were generally consistent with those observed for the primary outcome analyses. Vaccine uptake was faster in older individuals and in health and social care workers, but slower in individuals living in more deprived areas (HR of 1.52 [1.50–1.53] for comparison of least deprived with most deprived IMD quintile), in ethnic minority groups (HRs of 0.37–0.69 relative to White individuals), and in individuals who were housebound (HR 0.75 [0.74–0.76]); **Supplementary Figure 3** and **Supplementary Table 6**).

Clinical factors associated with eligibility for a third primary dose were also associated with faster 4-dose uptake, including immunosuppression (HR 1.16 [1.14–1.18]), haematologic cancer (HR 1.13 [1.11–1.16]) and transplant (HRs for 2.37 [2.30–2.45] and 1.56 [1.48–1.64] for kidney and non-kidney transplants, respectively). On the other hand, severe mental illness (HR 0.75 [0.72–0.77]) was associated with slower 4-dose uptake. Associations for demographic and clinical covariates were highly consistent across kidney disease subgroups, including RRT recipients (**Supplementary Figure 4** and **Supplementary Table 7**). Having a primary care code associated with stage 3–5 CKD was not associated with 4-dose uptake (HRs of 1.03–1.10 for the overall cohort, CKD subgroups, and dialysis recipients), but was associated with faster uptake in kidney transplant recipients (HR 1.15 [1.09–1.21], respectively).

Once again, stratified coverage estimates revealed several under-vaccinated subgroups (**Figure 3**). Among ethnic minority groups, 4-dose coverage ranged from 35% in the most deprived IMD quintile to 71% in the least deprived, with particularly low coverage (<50%) among individuals aged 16–64 years in all IMD quintiles (**Supplementary Table 8**). In White

For peer review only - http://bmjopen.bmj.com/site/about/guidelines.xhtml

individuals, 4-dose coverage was below 75% among individuals <70 years of age in all IMD quintiles, and among RRT recipients in the most deprived IMD quintiles.

Discussion

Summary

A principal aim of the COVID-19 vaccine roll-out is to protect those at greatest risk of severe disease. Based on the data presented here, considerable progress has been made towards achieving this goal among people with moderate-to-severe kidney disease in England, with 97.0% of individuals having received at least 2 doses of COVID-19 vaccine, 93.9% having received 3 doses, and 81.9% of eligible individuals having received 4 doses as of 31st August 2022.

Although high coverage has been achieved across people with kidney disease, considerable variation in vaccine uptake was evident in relation to the clinical and demographic characteristics of these individuals. Minority ethnicity and social deprivation were associated with delayed vaccine administration and lower final coverage, even in high-risk groups such as dialysis and kidney transplant recipients. Among clinical covariates, uptake was faster in individuals with immunosuppression, but slower in individuals with a history of severe mental illness. These patterns were consistent across stage 3–5 CKD and RRT subgroups, and affected both 3- and 4-dose uptake.

Findings in context

Inequities in COVID-19 vaccine uptake according to ethnicity and social deprivation have previously been reported for the general population[14], with similar disparities also impacting past national vaccination campaigns in the UK[18]. While our study is among the first to explore variation in COVID-19 vaccine uptake in people with kidney disease, several surveys of haemodialysis patients and kidney transplant recipients in France[19] and the USA[20-22] have reported higher COVID-19 vaccine hesitancy among ethnic minority groups and those living in more deprived areas – findings consistent with the delayed vaccine uptake in these groups reported here.

While prior studies have offered inconsistent findings with respect to the link between mental illness and COVID-19 vaccine uptake[14 23], these typically focused on 1-dose coverage in the early months of the vaccine roll-out, whereas the current study extends the window of follow-up throughout the primary and booster campaigns and focuses on a high-risk clinical group, limiting comparability.

Strengths and limitations

By harnessing the OpenSAFELY-TPP database, we incorporated linked health records across a population of 992,205 people with kidney disease, providing a comprehensive and timely view of the clinical and demographics factors associated with COVID-19 vaccine uptake in this high-risk population. We used eGFR levels to define CKD, thereby enabling us to avoid potential bias or imprecision associated with the inconsistent use of diagnostic codes across practices and regions, though the use and reporting of creatinine measurements may also be subject to regional inconsistency. The use of UKRR data also enabled us to audit vaccine

For peer review only - http://bmjopen.bmj.com/site/about/guidelines.xhtml

uptake among high-risk groups while reducing potential misclassification of current RRT status. Finally, we used complementary statistical modelling strategies to ensure our conclusions were robust to the assumptions of any single approach.

Nonetheless, several limitations of our study should be considered. First, the OpenSAFELY-TPP database does not offer a geographically representative sample of England, with regions such as London (3% of the present cohort) notably under-represented. Thus, region-specific variation in vaccine uptake may not be captured. Second, by considering completion of a 3-dose series as the primary endpoint, our Cox models may contravene the assumption of non-informative censoring, with the receipt of vaccine doses 1 and 2 resulting in a reduced likelihood of COVID-19-related mortality (and associated censoring). However, a sensitivity analysis restricted to uncensored individuals offered a consistent view of the factors associated with vaccine uptake at the end of follow-up. Third, individuals with CKD were identified based on a single serum creatinine measurement. This approach is highly sensitive and minimises delay in identification of affected individuals, but lacks confirmation of chronicity[24]. Finally, pre-existing conditions were considered as either present or absent, thereby failing to capture potentially informative details regarding the timing and severity of these conditions. This may have impacted our ability to accurately determine individuals eligible for a fourth COVID-19 vaccine dose, given that factors such as the timing of immunosuppressive therapy relative to vaccination are included in JCVI eligibility criteria[7].

Policy implications and interpretation

Our findings suggest that high-risk clinical risk groups do not escape the entrenched inequities shaping vaccine acceptance and uptake. Targeted activities to promote vaccine uptake among under-immunised communities have increasingly been used to expand the reach of the COVID-19 vaccine roll-out[25 26], and may yet be crucial towards enhancing booster dose coverage in people with kidney disease. Notably, under-vaccinated populations in the present study included: ethnic minority groups of any age or disease severity in areas of both low and high social deprivation; and White individuals <75 years of age in areas of both medium and high social deprivation. Furter research such as qualitative surveys within these under-vaccinated populations could offer a valuable opportunity to identify key barriers to vaccine uptake.

The prevalence of stage 3–5 CKD diagnostic codes varied from 49% in people with CKD3a to 88% in people with CKD4–5. These primary care codes may act as a proxy for GP, NHS, or personal awareness of CKD, which is often asymptomatic in its early stages. Indeed, coding status has previously been linked with improved primary care management of CKD, including delivery of influenza and pneumococcal vaccines[27]. Although we did not observe a strong link between CKD3–5 code and vaccine uptake in people with CKD3a or CKD3b, the presence of a diagnostic code was associated with earlier 3-dose uptake in people with CKD4–5 and those receiving RRT. This finding highlights the importance of consistent diagnostic code usage by GPs to promote timely vaccination of high-risk individuals.

Given the reduced immunogenicity of COVID-19 vaccines in people with kidney disease alongside the lower effectiveness of vaccines against the Omicron variant and subvariants [4 28], 3 doses may be insufficient to provide long-term protection in this high-risk population. A gradual increase in dose 4 coverage was apparent in early 2022, with a notable uptick

For peer review only - http://bmjopen.bmj.com/site/about/guidelines.xhtml

following the launch of the spring booster campaign in February 2022[14]. Although not all individuals in the present cohort would be eligible for a 4-dose series, 70% had at least one indicator for receipt of a fourth dose. Cumulative dose-4 coverage in these individuals was 81.9%, and fell short of this level in many subgroups, suggesting that concerted efforts to promote booster dose uptake among people with kidney disease remains essential. Evolving evidence regarding the risk of infection after primary vaccination among people with kidney disease serves as a clear mandate for the necessity of booster doses in these patients[5]. Vaccine-hesitant individuals report personal concerns over vaccine safety; therefore, clear communication about existing and ongoing research into the safety profile of primary and booster COVID-19 vaccine doses in individuals with kidney disease may help promote vaccine uptake in this population.

Conclusion

This study offers a comprehensive view of the status of the COVID-19 vaccine roll-out in a large cohort of people with kidney disease. To our knowledge, this is the first study to explore the link between individual-level characteristics and COVID-19 vaccine uptake at scale in this high-risk clinical group. Vaccine uptake was significantly slower among ethnic minority groups, in areas of higher social deprivation, and in people with severe mental illness. Addressing these disparities during ongoing and future booster campaigns will be essential to protect people with kidney disease from the most harmful sequelae of COVID-19.

Acknowledgements

We are very grateful for all the support received from the TPP Technical Operations team throughout this work, and for generous assistance from the information governance and database teams at NHS England/NHS Transformation Directorate.

Contributors

Conceptualisation: EPKP, EJC, FL, DN, and LAT; Methodology: EPKP, JT, WJH, LF, ACAG, VM, SS, EJW, BG, DN, and LAT; Software: WJH, CB, JC, SH, FH, JP, and AJW; Formal analysis, EPKP and JT; Data curation: CB, JC, SH, FH, AM, JP, SS, RS, AJW, and DN; Writing–original draft, EPKP; Writing–review & editing, EJC, HJC, EMFH, FL, SL, LN, SS, JACS, MW, BZ, DN, and LAT; Visualisation, EPKP; Supervision, DN and LAT; Project administration: EPKP and AM; Funding acquisition, EPKP, BG, DN, and LAT.

Funding

This work was jointly funded by the Wellcome Trust (222097/Z/20/Z); MRC (MR/V015757/1, MC_PC-20059, MR/W016729/1); NIHR (NIHR135559, COV-LT2-0073), and Health Data Research UK (HDRUK2021.000, 2021.0157). EPKP received funding from the UKRI COVID-19 Longitudinal Health and Wellbeing National Core Study (Phase 1 LHW-NCS, MC_PC-20059) through a secondment scheme. The views expressed are those of the authors and not necessarily those of the NIHR, NHS England, UK Health Security Agency or the Department of Health and Social Care. Funders had no role in the study design, collection, analysis, and interpretation of data; in the writing of the report; and in the decision to submit the article for publication.

Competing interests

For peer review only

BG's work on better use of data in healthcare more broadly is currently funded in part by: the Bennett Foundation, the Wellcome Trust, NIHR Oxford Biomedical Research Centre, NIHR Applied Research Collaboration Oxford and Thames Valley, the Mohn-Westlake Foundation; all Bennett Institute staff are supported by BG's grants on this work. BG is a Non-Executive Director at NHS Digital. EJW holds grants from MRC

Ethical approval

This study was approved by the Health Research Authority (REC reference 20/LO/0651) and by the London School of Hygiene & Tropical Medicine's Ethics Board (reference 21863).

Information governance

NHS England is the data controller for OpenSAFELY-TPP; TPP is the data processor; all study authors using OpenSAFELY have the approval of NHS England. This implementation of OpenSAFELY is hosted within the TPP environment which is accredited to the ISO 27001 information security standard and is NHS IG Toolkit compliant. Patient data has been pseudonymised for analysis and linkage using industry standard cryptographic hashing techniques; all pseudonymised datasets transmitted for linkage onto OpenSAFELY are encrypted; access to the platform is via a virtual private network (VPN) connection, restricted to a small group of researchers; the researchers hold contracts with NHS England and only access the platform to initiate database queries and statistical models; all database activity is logged; only aggregate statistical outputs leave the platform environment following best practice for anonymisation of results such as statistical disclosure control for low cell counts.

The OpenSAFELY research platform adheres to the obligations of the UK General Data Protection Regulation (GDPR) and the Data Protection Act 2018. In March 2020, the Secretary of State for Health and Social Care used powers under the UK Health Service (Control of Patient Information) Regulations 2002 (COPI) to require organisations to process confidential patient information for the purposes of protecting public health, providing healthcare services to the public and monitoring and managing the COVID-19 outbreak and incidents of exposure; this sets aside the requirement for patient consent.

Taken together, these provide the legal bases to link patient datasets on the OpenSAFELY platform. GP practices, from which the primary care data are obtained, are required to share relevant health information to support the public health response to the pandemic, and have been informed of the OpenSAFELY analytics platform.

This project includes data from the UKRR derived from patient-level information collected by the NHS as part of the care and support of kidney patients. We thank all kidney patients and kidney centres involved. The data are collated, maintained, and quality assured by the UKRR, which is part of the UK Kidney Association. Access to the data was facilitated by the UKRR's Data Release Group. UKRR data are used within OpenSAFELY to address a limited number of critical audit and service delivery questions related to the impact of COVID-19 on patients with kidney disease.

Data sharing

Detailed pseudonymised patient data is potentially re-identifiable and therefore not shared. Access to the underlying identifiable and potentially re-identifiable pseudonymised

For peer review only

For peer review only - http://bmjopen.bmj.com/site/about/guidelines.xhtml

electronic health record data is tightly governed by various legislative and regulatory frameworks, and restricted by best practice. The data in OpenSAFELY is drawn from General Practice data across England where TPP is the data processor. TPP developers initiate an automated process to create pseudonymised records in the core OpenSAFELY database, which are copies of key structured data tables in the identifiable records. These pseudonymised records are linked onto key external data resources that have also been pseudonymised via SHA-512 one-way hashing of NHS numbers using a shared salt. Bennett Institute for Applied Data Science developers and PIs holding contracts with NHS England have access to the OpenSAFELY pseudonymised data tables as needed to develop the OpenSAFELY tools. These tools in turn enable researchers with OpenSAFELY data access agreements to write and execute code for data management and data analysis without direct access to the underlying raw pseudonymised patient data, and to review the outputs of this code. All code for the full data management pipeline – from raw data to completed results for this analysis – and for the OpenSAFELY platform as a whole is available for review at github.com/OpenSAFELY.

Code availability

Data management was performed using Python, with analysis carried out using R 4.0.2. Code for data management and analysis, as well as codelists, are archived online (https://github.com/opensafely/ckd-coverage-ve).

Figure legends

Figure 1. Dose-specific coverage and product profile among people with kidney disease. (A) Cumulative dose-specific vaccine coverage over time. One minus Kaplan-Meier estimates are plotted for the receipt of any combination of vaccine products, censoring at death, deregistration, or 31st August 2022. For dose 4, we report overall coverage (solid line) and coverage among the subset of the cohort with at least one indicator for receipt of a fourth dose (3 primary + 1 booster or 2 primary + 2 boosters; dotted line). (B) Vaccine product distribution by dose.

Figure 2. Factors associated with completion of a 3-dose vaccine series in people with kidney disease. The left-hand panels display hazard ratios and 95% confidence intervals from partially adjusted models that included age, care home residence, health and social care worker status, housebound status, receipt of end-of-life care, setting (urban/rural), sex, ethnicity, IMD quintile, prior SARS-CoV-2 infection, immunosuppression, and haematologic cancer. Confidence intervals are generally too narrow to be visible. See **Supplementary Table 2** for minimally and fully adjusted model outputs. The right-hand panels display the cumulative coverage based on Kaplan-Meier estimates, censoring at death, deregistration, or 31st August 2022. CKD, chronic kidney disease; IMD, index of multiple deprivation; RRT, renal replacement therapy.

Figure 3. Cumulative 3- and 4-dose coverage in subgroups defined by deprivation index, ethnicity, age, and kidney disease severity. Cumulative coverage was determined based on Kaplan-Meier estimates, censoring at death, deregistration, or 31st August 2022.

For peer review only - http://bmjopen.bmj.com/site/about/guidelines.xhtml

Ethnic minority groups are combined due to their low combined prevalence within the study population. See **Supplementary Tables 4** and **8** for complete data. CKD, chronic kidney disease; dial., dialysis; IMD, index of multiple deprivation quintile; RRT, renal replacement therapy; Tx, transplant.

For peer review only

References

1. Williamson EJ, Walker AJ, Bhaskaran K, et al. Factors associated with COVID-19-related death using OpenSAFELY. *Nature* 2020;584(7821):430-36.
2. Ng JH, Hirsch JS, Wanchoo R, et al. Outcomes of patients with end-stage kidney disease hospitalized with COVID-19. *Kidney Int* 2020;98(6):1530-39.
3. Chen JJ, Lee TH, Tian YC, et al. Immunogenicity rates after SARS-CoV-2 vaccination in people with end-stage kidney disease: a systematic review and meta-analysis. *JAMA Netw Open* 2021;4(10):e2131749.
4. Carr EJ, Wu M, Harvey R, et al. Neutralising antibodies after COVID-19 vaccination in UK haemodialysis patients. *Lancet* 2021;398(10305):1038-41.
5. The OpenSAFELY Collaborative: Green A, Curtis H, et al. Describing the population experiencing COVID-19 vaccine breakthrough following second vaccination in England: A cohort study from OpenSAFELY. *BMC Med* 2022;20(1):243.
6. Public Health England. Chronic kidney disease prevalence model. Available from: https://assets.publishing.service.gov.uk/government/uploads/system/uploads/attachment_data/file/612303/ChronickidneydiseaseCKDprevalencemodelbriefing.pdf (accessed 28 November 2022).
7. JCVI. Joint Committee on Vaccination and Immunisation (JCVI) advice on third primary dose vaccination. Available from: https://www.gov.uk/government/publications/third-primary-covid-19-vaccine-dose-for-people-who-are-immunosuppressed-jcvi-advice/joint-committee-on-vaccination-and-immunisation-jcvi-advice-on-third-primary-dose-vaccination (accessed 12 April 2022).
8. JCVI. JCVI statement regarding a COVID-19 booster vaccine programme for winter 2021 to 2022. Available from: https://www.gov.uk/government/publications/jcvi-statement-september-2021-covid-19-booster-vaccine-programme-for-winter-2021-to-2022/jcvi-statement-regarding-a-covid-19-booster-vaccine-programme-for-winter-2021-to-2022 (accessed 12 April 2022).
9. JCVI. JCVI advice on COVID-19 booster vaccines for those aged 18 to 39 and a second dose for ages 12 to 15. Available from: https://www.gov.uk/government/news/jcvi-advice-on-covid-19-booster-vaccines-for-those-aged-18-to-39-and-a-second-dose-for-ages-12-to-15 (accessed 31 May 2022).
10. JCVI. JCVI advises a spring COVID-19 vaccine dose for the most vulnerable. Available from: https://www.gov.uk/government/news/jcvi-advises-a-spring-covid-19-vaccine-dose-for-the-most-vulnerable (accessed 14 April 2022).
11. Mathieu E, Ritchie H, Ortiz-Ospina E, et al. A global database of COVID-19 vaccinations. *Nat Hum Behav* 2021;5(7):947-53.
12. WHO. WHO Coronavirus (COVID-19) Dashboard. Available from: https://covid19.who.int (accessed 12 April 2022).
13. OpenSAFELY. NHS COVID-19 vaccine coverage: weekly report. Available from: https://opensafely.org/research/2021/covid-vaccine-coverage (accessed 12 April 2021).
14. Curtis HJ, Inglesby P, Morton CE, et al. Trends and clinical characteristics of COVID-19 vaccine recipients: a federated analysis of 57.9 million patients' primary care records in situ using OpenSAFELY. *Br J Gen Pract* 2022;72(714):e51-e62.
15. PRIMIS, University of Nottingham. SARS-CoV2 (COVID-19) vaccination uptake reporting specification (version 1). Available from: https://www.nottingham.ac.uk/primis/covid-19/covid-19.aspx (accessed 14 October 2021).

For peer review only - http://bmjopen.bmj.com/site/about/guidelines.xhtml

16. Levey AS, Stevens LA, Schmid CH, et al. A new equation to estimate glomerular filtration rate. *Ann Intern Med* 2009;150(9):604-12.
17. UK Health Security Agency. COVID-19: the green book, chapter 14a. Available from: https://www.gov.uk/government/publications/covid-19-the-green-book-chapter-14a (accessed 14 June 2022).
18. Roberts D, Matthews V, Saliba V, et al. National Immunisation Programme: health equity audit. Available from: https://assets.publishing.service.gov.uk/government/uploads/system/uploads/attachment_data/file/957670/immnstn-equity_AUDIT_v11.pdf (accessed 14 April 2022).
19. Andrian T, Koppe L, Novel E, et al. COVID-19 vaccine acceptance among haemodialysis patients: a French survey. *Clin Kidney J* 2021;14(8):1985-86.
20. Garcia P, Montez-Rath ME, Moore H, et al. SARS-CoV-2 vaccine acceptability in patients on hemodialysis: a nationwide survey. *J Am Soc Nephrol* 2021 doi: 10.1681/ASN.2021010104.
21. Tsapepas D, Husain SA, King KL, et al. Perspectives on COVID-19 vaccination among kidney and pancreas transplant recipients living in New York City. *Am J Health Syst Pharm* 2021;78(22):2040-45.
22. Tummalapalli SL, Cukor D, Bohmart A, et al. A mobile health-based survey to assess COVID-19 vaccine intent and uptake among patients on dialysis. *Kidney Int Rep* 2022;7(3):633-37.
23. Hassan L, Sawyer C, Peek N, et al. COVID-19 vaccination uptake in people with severe mental illness: a UK-based cohort study. *World Psychiatry* 2022;21(1):153-54.
24. Carrero JJ, Fu EL, Vestergaard SV, et al. Defining measures of kidney function in observational studies using routine health care data: methodological and reporting considerations. *Kidney Int* 2022; S0085-2538(22)00842-0.
25. England N. Bridging the Uptake Gap – COVID-19 vaccination toolkit for Black African and Black African Caribbean communities. Available from: https://www.england.nhs.uk/coronavirus/publication/bridging-the-uptake-gap-covid-19-vaccination-toolkit-for-black-african-and-black-african-caribbean-communities/ (accessed 14 April 2022).
26. Griffin S. Covid-19: Ethnicity vaccination gap narrows in England, but concerns remain. *BMJ* 2021;372:n505.
27. Kim LG, Cleary F, Wheeler DC, et al. How do primary care doctors in England and Wales code and manage people with chronic kidney disease? Results from the National Chronic Kidney Disease Audit. *Nephrol Dial Transplant* 2018;33(8):1373-79.
28. Andrews N, Stowe J, Kirsebom F, et al. Covid-19 Vaccine effectiveness against the Omicron (B.1.1.529) variant. *N Engl J Med* 2022;386(16):1532-46.

For peer review only - http://bmjopen.bmj.com/site/about/guidelines.xhtml

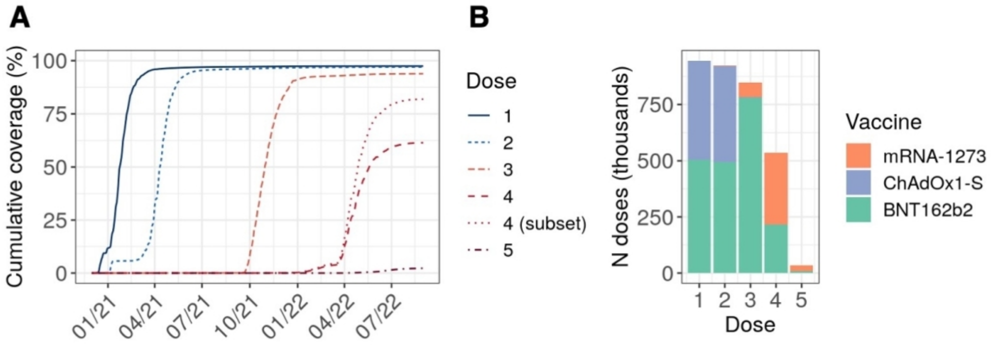

Revised Figure 1

213x76mm (185 x 185 DPI)

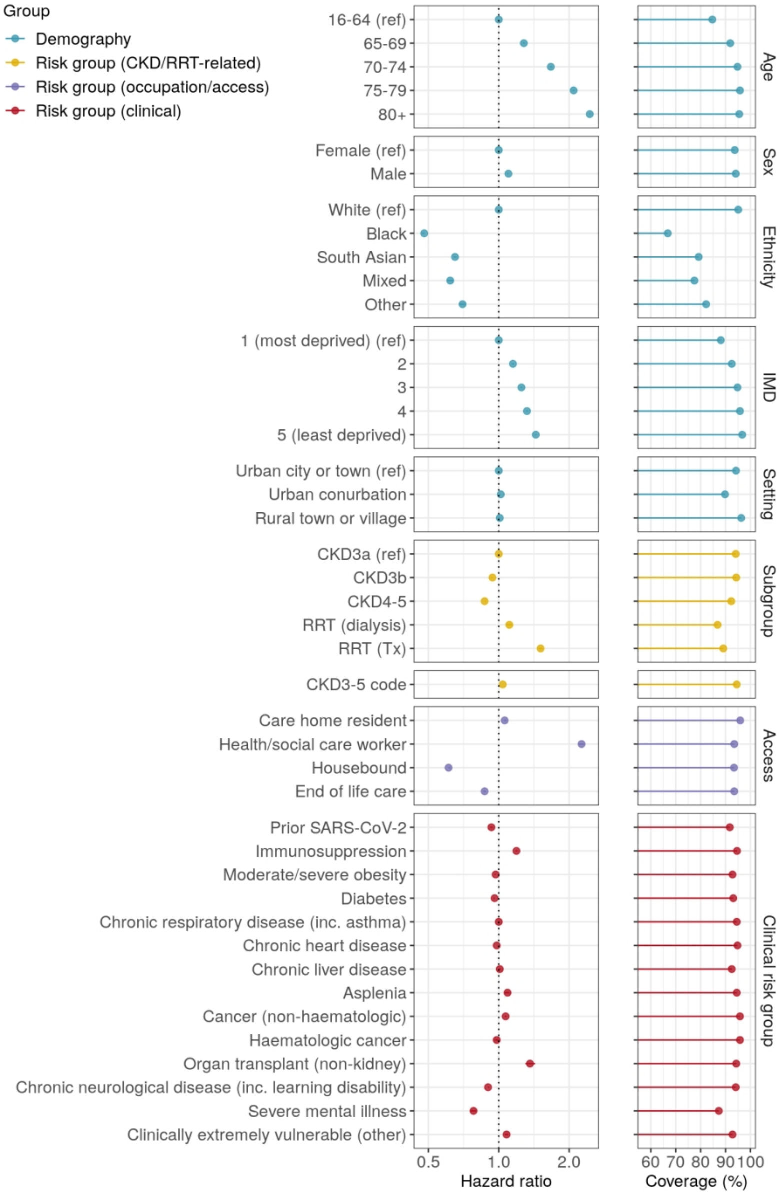

Revised Figure 2

513x770mm (76 x 76 DPI)

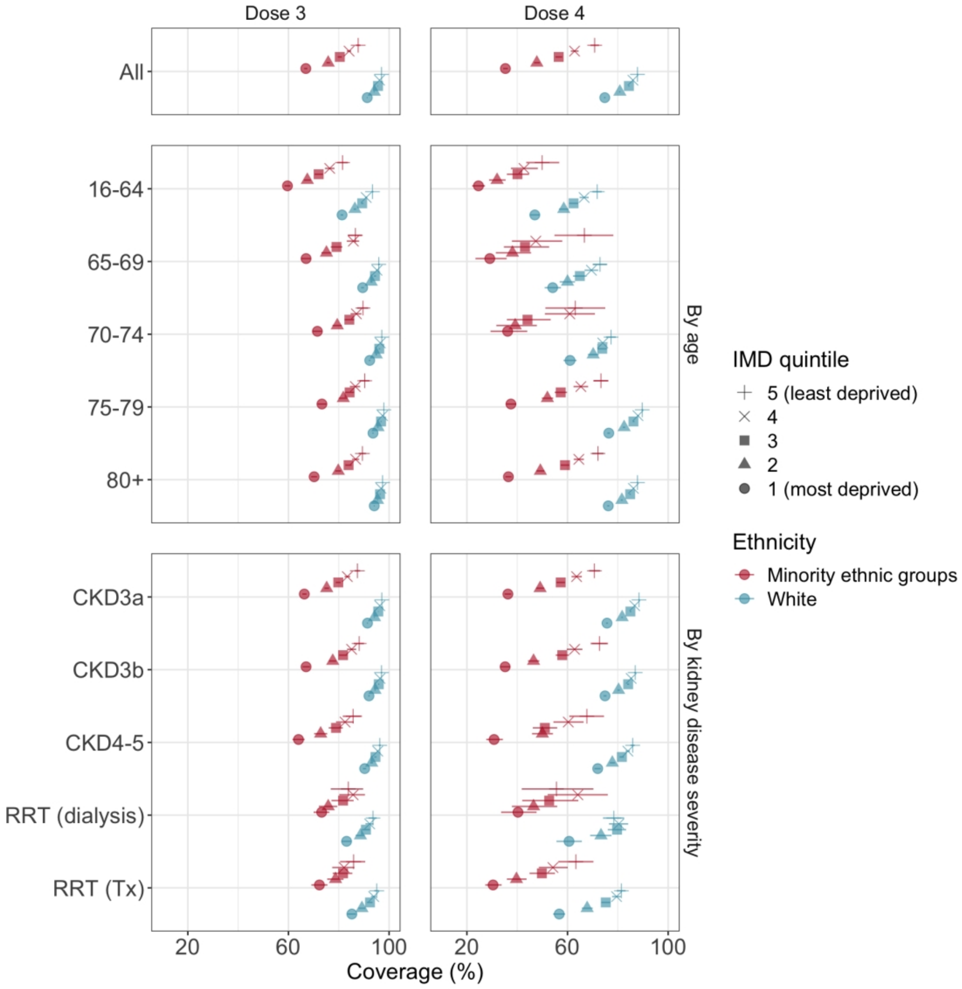

Revised Figure 3

677x677mm (72 x 72 DPI)

Supplementary Appendix

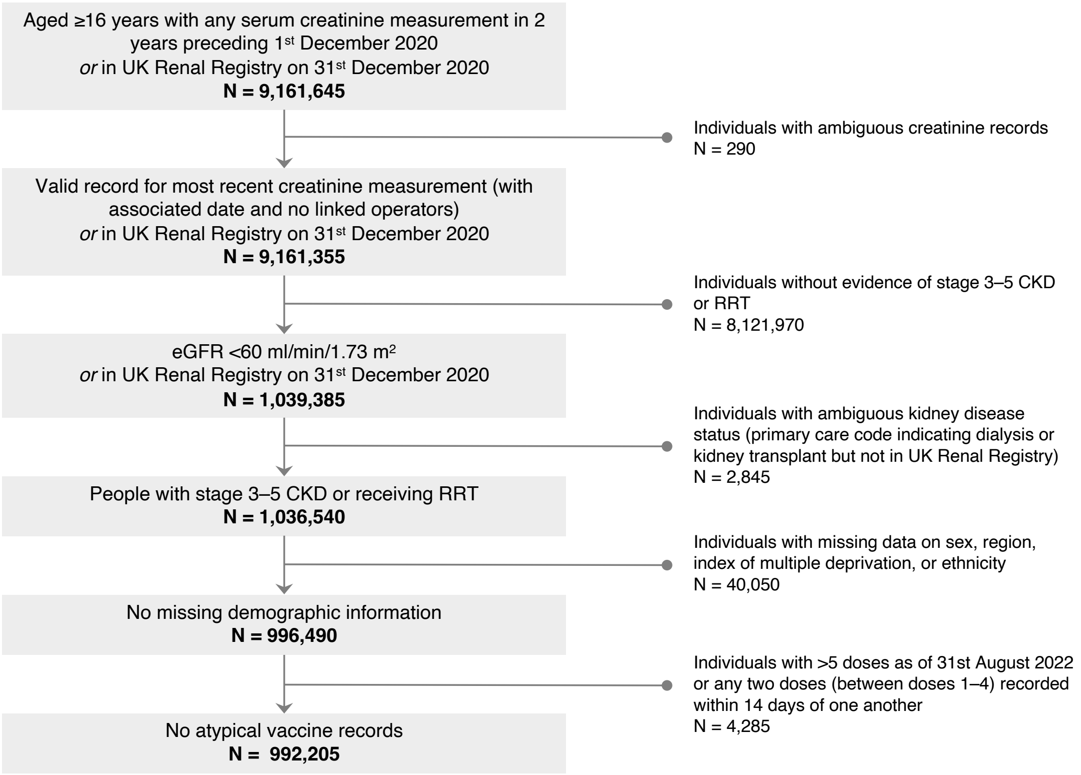

Supplementary Figure 1. Flow chart of study eligibility. Frequencies are rounded to the nearest 5. CKD, chronic kidney disease; eGFR, estimated glomerular filtrate rate; RRT, renal replacement therapy.

review only

For peer review only - http://bmjopen.bmj.com/site/about/guidelines.xhtml

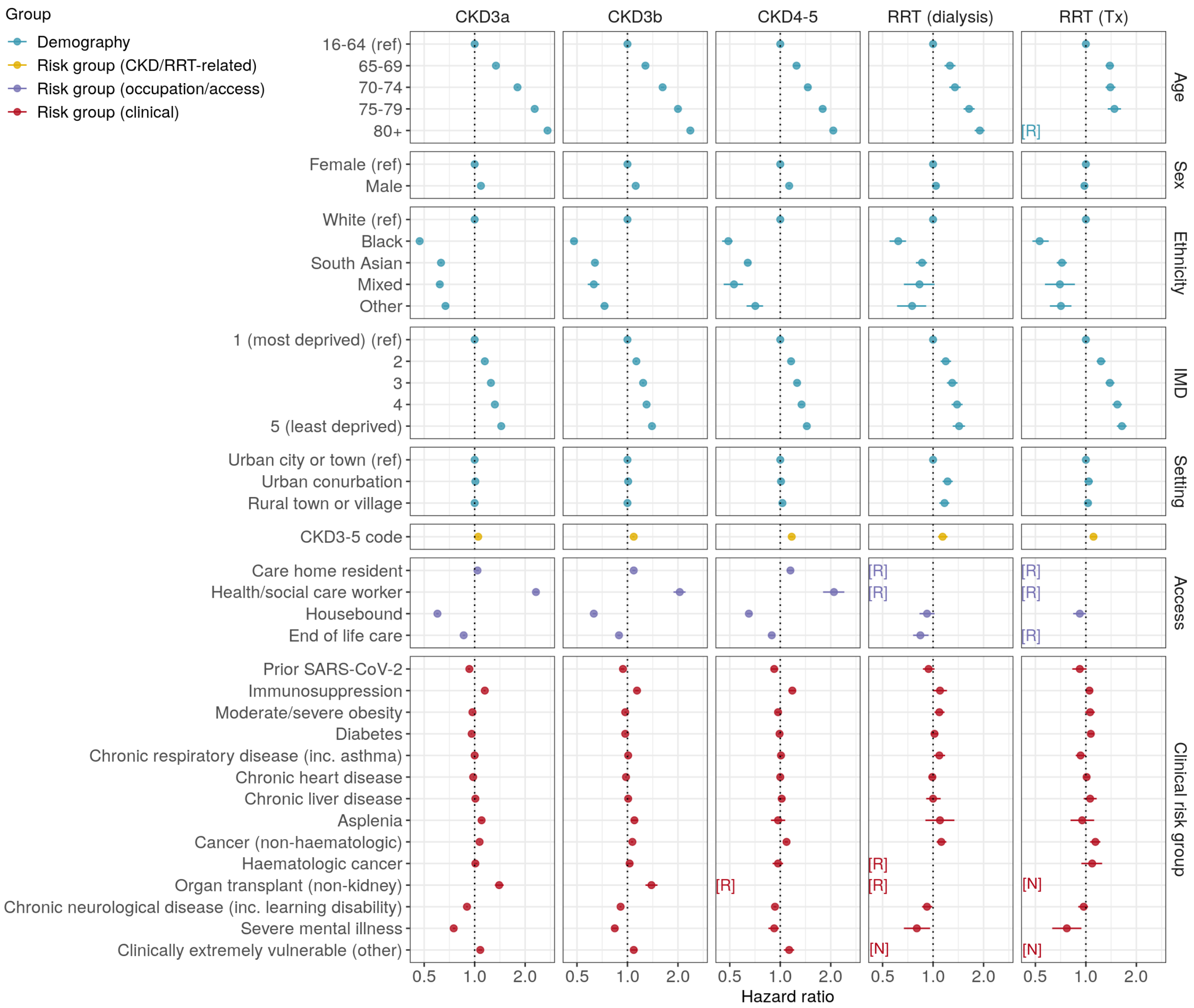

Supplementary Figure 2. Factors associated with completion of a 3-dose vaccine series in kidney disease subgroups. Hazard ratios and 95% confidence intervals were derived from partially adjusted models that included age, care home residence, health and social care worker status, housebound status, receipt of end-of-life care, setting (urban/rural), sex, ethnicity, IMD quintile, prior SARS-CoV-2 infection, immunosuppression, and haematologic cancer. Data are redacted [R] for any rows in which there were >0 and ≤10 event or non-event counts after rounding to the nearest 5, with non-event counts calculated among uncensored individuals. CKD, chronic kidney disease; IMD, index of multiple deprivation; [N], covariate absent in all individuals by definition; [R], redacted; RRT, renal replacement therapy; Tx, transplant.

For peer review only - http://bmjopen.bmj.com/site/about/guidelines.xhtml

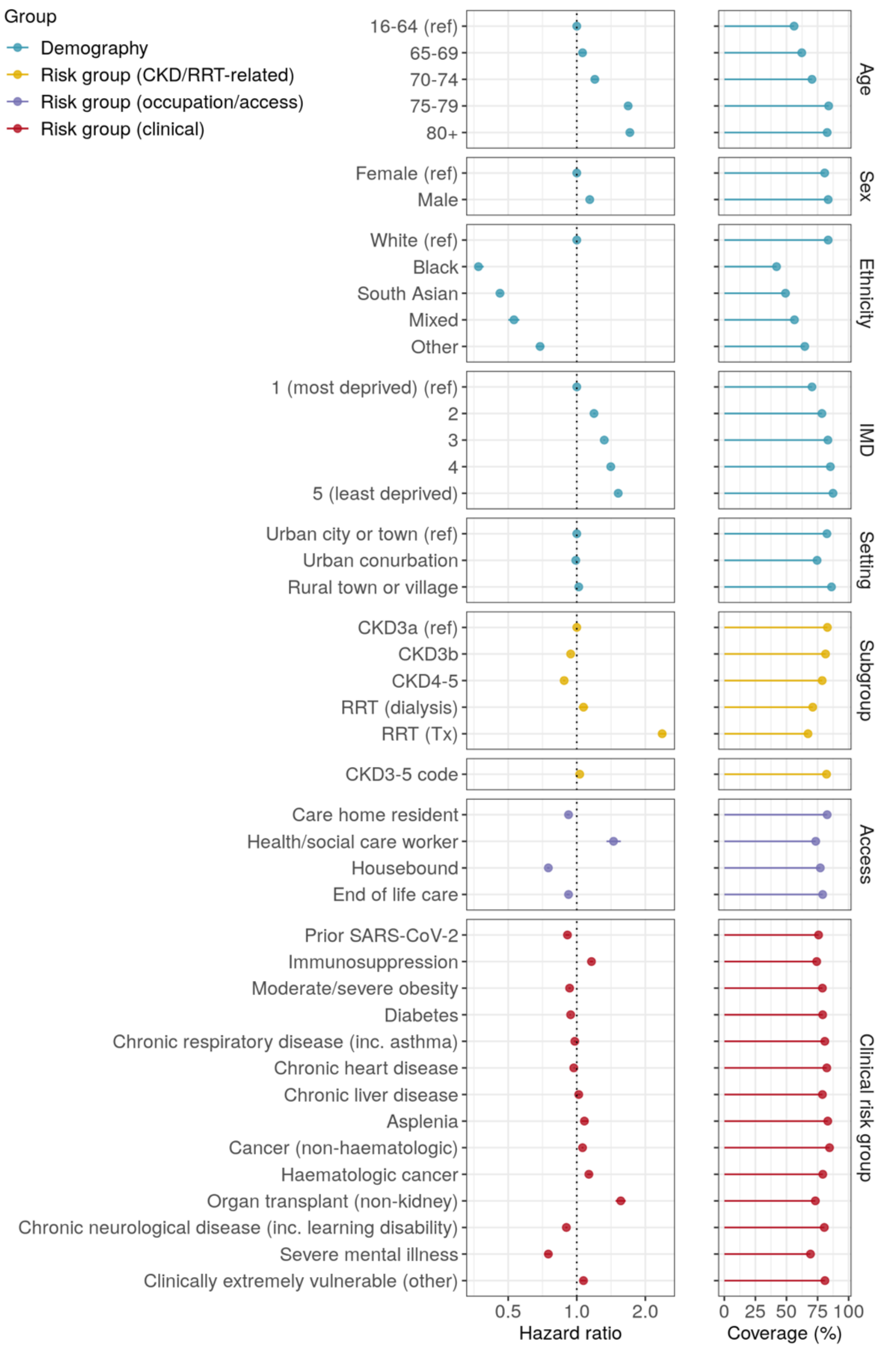

Supplementary Figure 3. Factors associated with completion of a 4-dose vaccine series in people with kidney disease. The left-hand panels display hazard ratios and 95% confidence intervals from partially adjusted models that included age, care home residence, health and social care worker status, housebound status, receipt of end-of-life care, setting (urban/rural), sex, ethnicity, IMD quintile, prior SARS-CoV-2 infection, immunosuppression, and haematologic cancer. Confidence intervals are generally too narrow to be visible. See **Supplementary Table 6** for minimally and fully adjusted model outputs. The right-hand panels display the cumulative coverage based on Kaplan-Meier estimates, censoring at death, deregistration, or 31st August 2022. CKD, chronic kidney disease; IMD, index of multiple deprivation; RRT, renal replacement therapy.

For peer review only - http://bmjopen.bmj.com/site/about/guidelines.xhtml

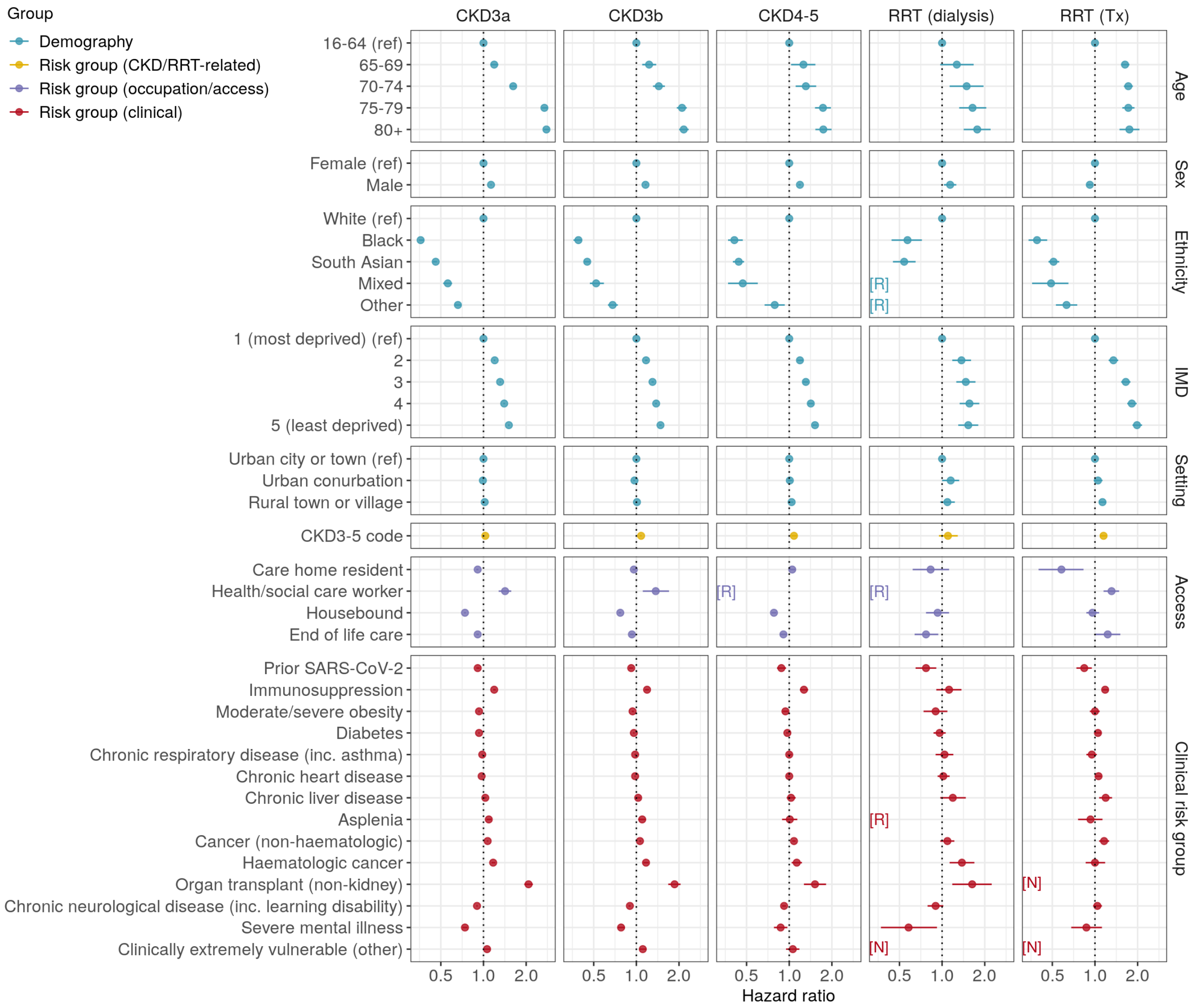

Supplementary Figure 4. Factors associated with completion of a 4-dose vaccine series in kidney disease subgroups. Individuals were included in the analysis if they were ≥75 years of age at baseline, care home residents, transplant recipients, or had a history of haematologic malignancy or immunosuppression. Hazard ratios and 95% confidence intervals were derived from partially adjusted models that included age, care home residence, health and social care worker status, housebound status, receipt of end-of-life care, setting (urban/rural), sex, ethnicity, IMD quintile, prior SARS-CoV-2 infection, immunosuppression, and haematologic cancer. Data are redacted [R] for any rows in which there were >0 and ≤10 event or non-event counts after rounding to the nearest 5, with non-event counts calculated among uncensored individuals. CKD, chronic kidney disease; IMD, index of multiple deprivation; [N], covariate absent in all individuals by definition; [R], redacted; RRT, renal replacement therapy; Tx, transplant.

For peer review only - http://bmjopen.bmj.com/site/about/guidelines.xhtml

Supplementary Table 1. Product profile by dose.

Subgroup	Dose 1, n (%)	Dose 2, n (%)	Dose 3, n (%)	Dose 4, n (%)
**N**	944,605 (100)	922,365 (100)	849,150 (100)	535,975 (100)
**Product**				
BNT	438,890 (53.5)	495,375 (53.7)	779,740 (91.8)	216,435 (40.4)
AZ	438,890 (46.5)	425,820 (46.2)	1,965 (0.2)	200 (0.0)
MOD	735 (0.1)	1,170 (0.1)	67,445 (7.9)	319,340 (59.6)
**2-dose product combination**				
BNT-BNT	–	492,105 (53.4)	–	–
AZ-AZ	–	422,145 (45.8)	–	–
BNT-AZ	–	3,675 (0.4)	–	–
AZ-BNT	–	3,215 (0.3)	–	–
Other	–	1,225 (0.1)	–	–
**3-dose product combination**				
BNT-BNT-BNT	–	–	438,905 (51.7)	–
AZ-AZ-BNT	–	–	336,725 (39.7)	–
AZ-AZ-MOD	–	–	44,430 (5.2)	–
BNT-BNT-MOD	–	–	21,970 (2.6)	–
BNT-AZ-BNT	–	–	2,575 (0.3)	–
AZ-AZ-AZ	–	–	1,720 (0.2)	–
AZ-BNT-BNT	–	–	1,320 (0.2)	–
Other	–	–	1,510 (0.2)	–

Frequencies are rounded to the nearest 5. Any product combinations given to <1000 individuals were pooled as 'Other'. AZ, ChAdOx1-S (AstraZeneca); BNT, BNT162b2 (Pfizer-BioNTech); MOD, mRNA-1273 (Moderna).

For peer review only

Supplementary Table 2. Cox proportional hazards model and logistic regression outputs for factors associated with completion of a 3-dose vaccination series in people with kidney disease.

	Cox models					Logistic regression	
**Variable**	**N (n events)**	**Cov. (%)**	**HR (95% CI), minimally adjusted**	**HR (95% CI), partially adjusted**	**HR (95% CI), fully adjusted**	**N (n events)**	**OR (95% CI), partially adjusted**
**Age**							
16–64 (ref)	109,815 (89,610)	84.7	1.00	1.00	1.00	103,215 (87,745)	1.00
65–69	74,740 (65,845)	91.9	1.32 (1.31-1.34)	1.28 (1.27-1.29)	1.31 (1.30-1.32)	69,495 (64,110)	1.85 (1.79-1.92)
70–74	143,575 (129,880)	94.8	1.76 (1.75-1.78)	1.67 (1.66-1.69)	1.71 (1.70-1.73)	132,385 (125,905)	2.67 (2.59-2.75)
75–79	181,200 (163,730)	95.8	2.20 (2.18-2.21)	2.09 (2.07-2.11)	2.15 (2.14-2.17)	162,940 (156,740)	3.38 (3.27-3.49)
80+	482,870 (399,765)	95.5	2.47 (2.45-2.49)	2.45 (2.44-2.47)	2.56 (2.54-2.58)	370,940 (356,715)	3.39 (3.30-3.48)
**Sex**							
Female (ref)	555,365 (476,420)	93.7	1.00	1.00	1.00	473,535 (445,645)	1.00
Male	436,840 (372,415)	94.1	1.11 (1.11-1.12)	1.10 (1.09-1.10)	1.10 (1.09-1.10)	365,440 (345,565)	1.18 (1.16-1.20)
**Ethnicity**							
White (ref)	930,565 (805,890)	95.1	1.00	1.00	1.00	785,470 (750,345)	1.00
Black	17,675 (11,030)	66.8	0.45 (0.44-0.46)	0.48 (0.47-0.48)	0.48 (0.47-0.49)	15,705 (10,590)	0.19 (0.19-0.20)
South Asian	32,620 (23,560)	79.2	0.61 (0.60-0.62)	0.65 (0.64-0.66)	0.65 (0.64-0.66)	28,000 (22,365)	0.32 (0.31-0.33)
Mixed	4,755 (3,435)	77.5	0.60 (0.58-0.62)	0.62 (0.60-0.64)	0.62 (0.60-0.65)	4,215 (3,290)	0.29 (0.26-0.31)
Other	6,590 (4,920)	82.2	0.68 (0.66-0.70)	0.70 (0.68-0.72)	0.69 (0.67-0.71)	5,585 (4,625)	0.33 (0.31-0.36)
**IMD**							
1 most deprived (ref)	164,415 (130,595)	88.1	1.00	1.00	1.00	136,680 (121,160)	1.00
2	187,240 (156,820)	92.5	1.19 (1.18-1.20)	1.15 (1.14-1.16)	1.14 (1.14-1.15)	157,145 (146,105)	1.48 (1.44-1.52)
3	224,590 (194,255)	94.8	1.32 (1.31-1.33)	1.25 (1.24-1.26)	1.24 (1.23-1.25)	190,175 (181,085)	1.90 (1.84-1.95)
4	216,980 (190,310)	95.8	1.40 (1.39-1.42)	1.32 (1.31-1.33)	1.31 (1.30-1.32)	184,545 (177,440)	2.18 (2.11-2.25)
5 least deprived	198,985 (176,855)	96.7	1.53 (1.52-1.54)	1.44 (1.43-1.45)	1.42 (1.41-1.43)	170,425 (165,420)	2.74 (2.64-2.83)
**Setting**							
Urban city or town (ref)	530,975 (456,140)	94.2	1.00	1.00	1.00	449,315 (425,415)	1.00
Urban conurbation	204,530 (166,040)	89.8	0.93 (0.93-0.94)	1.02 (1.01-1.02)	1.01 (1.01-1.02)	170,720 (154,285)	0.84 (0.82-0.86)
Rural town or village	256,700 (226,650)	96.3	1.08 (1.07-1.09)	1.01 (1.00-1.01)	1.01 (1.00-1.01)	218,940 (211,515)	1.18 (1.14-1.21)
**Kidney disease subgroup**							
CKD3a (ref)	659,785 (579,655)	94.1	1.00	1.00	1.00	581,900 (549,290)	1.00
CKD3b	245,380 (203,490)	94.3	0.92 (0.92-0.93)	0.94 (0.93-0.94)	0.93 (0.92-0.93)	194,630 (184,545)	0.95 (0.93-0.97)
CKD4–5	64,855 (47,985)	92.3	0.84 (0.83-0.85)	0.87 (0.87-0.88)	0.86 (0.85-0.87)	44,025 (41,015)	0.87 (0.83-0.90)
RRT (dialysis)	9,455 (6,925)	86.8	1.03 (1.00-1.05)	1.11 (1.08-1.14)	1.09 (1.06-1.11)	6,875 (6,015)	1.13 (1.04-1.22)
RRT (transplant)	12,735 (10,780)	89.1	1.53 (1.50-1.56)	1.51 (1.48-1.54)	1.48 (1.45-1.51)	11,545 (10,340)	1.23 (1.15-1.31)
**Primary care coding of kidney disease**							
CKD diagnostic code	592,520 (507,470)	94.5	1.03 (1.02-1.03)	1.04 (1.04-1.04)	1.07 (1.06-1.07)	494,910 (469,965)	1.25 (1.23-1.28)
**Risk group (occupation/access)**							
Care home resident	44,470 (28,155)	95.9	1.02 (1.00-1.03)	1.06 (1.05-1.08)	1.11 (1.10-1.13)	21,100 (20,320)	1.25 (1.16-1.35)
Health/social care worker	5,680 (5,245)	93.5	2.12 (2.06-2.18)	2.26 (2.20-2.32)	2.24 (2.18-2.30)	5,515 (5,165)	2.67 (2.38-2.99)
Housebound	54,600 (38,340)	93.4	0.60 (0.59-0.60)	0.61 (0.61-0.62)	0.63 (0.62-0.63)	33,090 (31,195)	0.85 (0.81-0.90)

For peer review only

End of life care	40,400 (24,220)	93.5	0.82 (0.81-0.84)	0.87 (0.86-0.88)	0.87 (0.86-0.88)	19,445 (18,405)	0.89 (0.83-0.95)
**Risk group (clinical)**							
Prior SARS-CoV-2	27,835 (19,630)	91.7	0.88 (0.87-0.90)	0.93 (0.92-0.94)	0.94 (0.92-0.95)	18,470 (17,040)	1.02 (0.96-1.08)
Immunosuppression	56,985 (47,850)	94.6	1.17 (1.15-1.18)	1.19 (1.18-1.20)	1.13 (1.11-1.14)	46,000 (43,730)	1.47 (1.39-1.54)
Moderate/severe obesity	111,630 (96,740)	92.8	0.93 (0.93-0.94)	0.97 (0.96-0.97)	0.98 (0.97-0.99)	99,230 (92,410)	1.10 (1.07-1.13)
Diabetes	275,745 (229,435)	93.1	0.91 (0.90-0.91)	0.96 (0.95-0.96)	0.97 (0.96-0.97)	225,340 (211,050)	1.09 (1.06-1.11)
CRD (inc. asthma)	120,935 (98,590)	94.5	0.98 (0.97-0.98)	1.00 (0.99-1.01)	1.00 (1.00-1.01)	92,980 (88,445)	1.08 (1.04-1.11)
Chronic heart disease	438,965 (366,070)	94.8	0.98 (0.98-0.99)	0.98 (0.98-0.98)	0.99 (0.98-0.99)	348,165 (332,040)	1.08 (1.05-1.10)
Chronic liver disease	32,215 (27,010)	92.5	1.00 (0.99-1.01)	1.01 (1.00-1.02)	1.01 (1.00-1.02)	27,140 (25,240)	1.04 (0.99-1.09)
Asplenia	8,665 (7,440)	94.5	1.10 (1.07-1.12)	1.09 (1.06-1.11)	1.08 (1.06-1.11)	7,345 (6,970)	1.18 (1.06-1.31)
Cancer (non-haematologic)	168,840 (141,125)	95.8	1.09 (1.08-1.10)	1.07 (1.06-1.08)	1.07 (1.07-1.08)	133,245 (128,420)	1.34 (1.30-1.38)
Haematologic cancer	20,430 (16,740)	95.8	1.16 (1.14-1.18)	0.98 (0.96-1.00)	1.03 (1.01-1.05)	15,450 (14,895)	1.04 (0.94-1.15)
Organ transplant (non-kidney)	2,190 (1,895)	94.3	1.45 (1.38-1.51)	1.36 (1.30-1.43)	1.41 (1.35-1.48)	1,880 (1,780)	1.68 (1.36-2.07)
CND (inc. learning disability)	163,135 (129,130)	94.1	0.89 (0.88-0.89)	0.90 (0.90-0.91)	0.91 (0.90-0.91)	119,585 (113,255)	0.92 (0.90-0.95)
Severe mental illness	12,985 (10,105)	87.3	0.75 (0.73-0.76)	0.78 (0.76-0.80)	0.79 (0.77-0.80)	10,575 (9,280)	0.65 (0.61-0.69)
CEV (other)	9,065 (7,790)	92.8	1.04 (1.02-1.06)	1.08 (1.06-1.10)	1.06 (1.04-1.09)	7,795 (7,265)	1.02 (0.93-1.12)

Cumulative coverage was determined based on Kaplan-Meier estimates, censoring at death, deregistration, or 31st August 2022. Logistic regression models included 838,975 individuals who were uncensored (registered and alive) as of the analysis cut-off (31st August 2022). Minimally adjusted models included age, care home residence, and health and social care worker status given their use in vaccine prioritisation criteria. Partially adjusted models additionally included housebound status, receipt of end-of-life care, setting, sex, ethnicity, IMD quintile, prior SARS-CoV-2 infection, immunosuppression, and haematologic cancer. Fully adjusted models included all covariates. CI, confidence interval; CKD, chronic kidney disease; HR, hazard ratio; IMD, index of multiple deprivation; OR, odds ratio; RRT, renal replacement therapy.

For peer review only

Supplementary Table 3. Cox proportional hazards models for factors associated with completion of a 3-dose vaccination series in kidney disease subgroups.

	CKD3a		CKD3b		CKD4-5		RRT (dialysis)		RRT (transplant)	
**Variable**	**Cov. (%)**	**HR (95% CI)**	**Cov. (%)**	**HR (95% CI)**	**Cov. (%)**	**HR (95% CI)**	**Cov. (%)**	**HR (95% CI)**	**Cov. (%)**	**HR (95% CI)**
**Age**										
16–64 (ref)	85.1	1.00	83.1	1.00	81.4	1.00	80.8	1.00	87.2	1.00
65–69	92.3	1.34 (1.32-1.35)	90.8	1.28 (1.24-1.31)	88.9	1.25 (1.19-1.32)	88.7	1.26 (1.17-1.36)	94.1	1.39 (1.31-1.47)
70–74	95.1	1.80 (1.78-1.82)	94.1	1.62 (1.58-1.66)	91.9	1.46 (1.40-1.52)	91.2	1.35 (1.25-1.46)	94.5	1.40 (1.31-1.50)
75–79	96.0	2.28 (2.25-2.30)	95.4	2.00 (1.96-2.05)	94.0	1.79 (1.72-1.86)	95.0	1.64 (1.52-1.77)	94.3	1.48 (1.35-1.62)
80+	95.7	2.72 (2.70-2.75)	95.4	2.37 (2.32-2.41)	94.3	2.07 (2.00-2.13)	95.0	1.90 (1.77-2.03)	[R]	[R]
**Sex**										
Female (ref)	93.9	1.00	94.0	1.00	92.1	1.00	85.4	1.00	89.2	1.00
Male	94.3	1.09 (1.08-1.09)	94.6	1.12 (1.11-1.13)	92.5	1.13 (1.11-1.15)	87.6	1.04 (0.99-1.09)	89.0	0.98 (0.94-1.02)
**Ethnicity**										
White (ref)	95.2	1.00	95.3	1.00	94.1	1.00	89.5	1.00	91.6	1.00
Mixed	66.0	0.47 (0.46-0.48)	68.6	0.48 (0.46-0.50)	66.0	0.49 (0.45-0.52)	70.2	0.62 (0.55-0.69)	69.9	0.53 (0.48-0.60)
Asian or Asian British	79.6	0.63 (0.62-0.64)	78.7	0.64 (0.63-0.66)	75.3	0.64 (0.61-0.67)	81.6	0.86 (0.79-0.92)	80.2	0.72 (0.67-0.77)
Black or Black British	77.3	0.62 (0.59-0.64)	79.2	0.63 (0.58-0.68)	69.7	0.53 (0.46-0.60)	81.8	0.83 (0.67-1.02)	79.2	0.70 (0.57-0.86)
Other ethnic groups	82.3	0.67 (0.65-0.70)	83.6	0.73 (0.69-0.77)	79.3	0.71 (0.63-0.79)	70.1	0.75 (0.61-0.91)	80.3	0.71 (0.61-0.82)
**IMD**										
1 most deprived (ref)	88.3	1.00	89.1	1.00	86.0	1.00	79.6	1.00	81.2	1.00
2	92.6	1.15 (1.14-1.16)	93.1	1.13 (1.11-1.14)	90.8	1.16 (1.12-1.19)	85.3	1.19 (1.11-1.28)	86.7	1.23 (1.16-1.31)
3	94.9	1.25 (1.24-1.26)	95.2	1.24 (1.22-1.26)	93.4	1.26 (1.22-1.30)	89.1	1.30 (1.21-1.40)	90.9	1.39 (1.31-1.48)
4	95.9	1.32 (1.31-1.33)	96.0	1.30 (1.28-1.32)	95.0	1.34 (1.30-1.38)	92.3	1.39 (1.29-1.50)	92.7	1.54 (1.44-1.64)
5 least deprived	96.8	1.44 (1.43-1.45)	96.8	1.40 (1.38-1.42)	96.0	1.44 (1.39-1.48)	93.1	1.43 (1.31-1.55)	94.6	1.64 (1.53-1.75)
**Setting**										
Urban city or town (ref)	94.4	1.00	94.6	1.00	93.0	1.00	86.5	1.00	89.9	1.00
Urban conurbation	90.2	1.01 (1.01-1.02)	90.5	1.01 (1.00-1.02)	87.1	1.01 (0.98-1.03)	83.1	1.22 (1.14-1.31)	84.0	1.04 (0.98-1.10)
Rural town or village	96.4	1.00 (1.00-1.01)	96.5	1.00 (0.99-1.01)	95.6	1.03 (1.00-1.05)	94.1	1.17 (1.09-1.25)	93.5	1.03 (0.98-1.08)
**Primary care coding of kidney disease**										
CKD diagnostic code	94.9	1.05 (1.04-1.06)	94.7	1.09 (1.07-1.10)	92.8	1.17 (1.13-1.20)	88.1	1.14 (1.07-1.22)	90.1	1.11 (1.06-1.16)
**Risk group (occupation/access)**										
Care home resident	95.9	1.04 (1.02-1.06)	96.1	1.09 (1.07-1.12)	94.9	1.15 (1.10-1.20)	[R]	[R]	[R]	[R]
Health/social care worker	93.7	2.32 (2.25-2.40)	91.9	2.05 (1.88-2.22)	90.2	2.09 (1.80-2.41)	[R]	[R]	[R]	[R]
Housebound	93.6	0.60 (0.59-0.61)	93.7	0.63 (0.62-0.65)	92.0	0.65 (0.63-0.68)	90.5	0.92 (0.83-1.02)	91.2	0.92 (0.84-1.00)
End of life care	93.7	0.86 (0.85-0.88)	93.9	0.89 (0.87-0.91)	92.6	0.89 (0.85-0.93)	86.5	0.84 (0.76-0.94)	[R]	[R]
**Risk group (clinical)**										
Prior SARS-CoV-2	92.3	0.93 (0.91-0.94)	92.7	0.94 (0.91-0.96)	89.8	0.92 (0.88-0.97)	83.2	0.94 (0.87-1.02)	83.4	0.92 (0.83-1.01)
Immunosuppression	95.4	1.15 (1.13-1.16)	95.5	1.14 (1.11-1.17)	93.6	1.18 (1.12-1.24)	86.9	1.10 (1.00-1.21)	89.4	1.05 (1.00-1.09)
Moderate/severe obesity	92.8	0.97 (0.97-0.98)	93.2	0.97 (0.96-0.99)	91.4	0.97 (0.94-1.00)	88.2	1.09 (1.02-1.17)	88.7	1.06 (0.99-1.13)
Diabetes	93.3	0.96 (0.95-0.96)	93.4	0.97 (0.96-0.98)	91.7	0.99 (0.97-1.01)	88.1	1.02 (0.97-1.07)	90.1	1.07 (1.03-1.12)
CRD (inc. asthma)	94.6	1.00 (0.99-1.00)	94.8	1.01 (1.00-1.02)	93.2	1.01 (0.98-1.04)	92.0	1.09 (1.01-1.18)	89.3	0.93 (0.87-1.00)
Chronic heart disease	95.1	0.98 (0.98-0.99)	94.9	0.98 (0.97-0.99)	93.5	1.00 (0.98-1.02)	88.3	0.99 (0.94-1.04)	90.6	1.01 (0.97-1.05)
Chronic liver disease	92.7	1.01 (1.00-1.03)	92.8	1.01 (0.99-1.04)	91.3	1.02 (0.97-1.07)	83.3	1.00 (0.91-1.11)	89.9	1.06 (0.97-1.16)
Asplenia	95.1	1.10 (1.07-1.13)	94.4	1.10 (1.05-1.15)	90.2	0.97 (0.88-1.07)	84.4	1.10 (0.90-1.34)	87.3	0.95 (0.81-1.12)
Cancer (non-haematologic)	95.9	1.07 (1.07-1.08)	96.0	1.07 (1.05-1.08)	94.8	1.09 (1.06-1.11)	92.3	1.12 (1.05-1.20)	93.7	1.14 (1.06-1.22)
Haematologic cancer	95.9	1.01 (0.99-1.04)	95.8	1.03 (0.99-1.07)	94.1	0.97 (0.90-1.04)	[R]	[R]	90.3	1.09 (0.94-1.25)
Organ transplant (non-kidney)	93.4	1.40 (1.32-1.49)	94.4	1.39 (1.28-1.51)	[R]	[R]	[R]	[R]	[N]	[N]
CND (inc. learning disability)	94.3	0.90 (0.89-0.91)	94.3	0.91 (0.90-0.92)	92.6	0.93 (0.91-0.95)	87.5	0.92 (0.86-0.99)	90.9	0.97 (0.90-1.03)
Severe mental illness	86.2	0.75 (0.74-0.77)	90.6	0.84 (0.81-0.87)	87.7	0.92 (0.85-0.98)	73.3	0.80 (0.67-0.96)	76.6	0.77 (0.63-0.94)

For peer review only

CEV (other)	93.1	1.08 (1.05-1.11)	93.6	1.09 (1.05-1.15)	89.4	1.13 (1.05-1.21)	[N]	[N]	[N]	[N]

Counts are rounded to the nearest 5. Cumulative coverage was determined based on Kaplan-Meier estimates, censoring at death, deregistration, or 31st August 2022. Hazard ratios and 95% confidence intervals were derived from partially adjusted models that included age, care home residence, health and social care worker status, housebound status, receipt of end-of-life care, setting (urban/rural), sex, ethnicity, IMD quintile, prior SARS-CoV-2 infection, immunosuppression, and haematologic cancer. Data are redacted [R] for any rows in which there were >0 and ≤10 event or non-event counts after rounding to the nearest 5, with non-event counts calculated among uncensored individuals. CEV, clinically extremely vulnerable; CKD, chronic kidney disease; CND, chronic neurological disease; IMD, index of multiple deprivation; [N], covariate absent in all individuals by definition; [R] redacted; RRT, renal replacement therapy.

For peer review only - http://bmjopen.bmj.com/site/about/guidelines.xhtml

For peer review only

Supplementary Table 4. Cumulative 3-dose coverage in subgroups defined by deprivation index, ethnicity, age, and kidney disease severity.

	IMD 1 (most deprived)				IMD 2				IMD 3				IMD 4				IMD 5 (least deprived)			
	White		Ethnic minority groups		White		Ethnic minority groups		White		Ethnic minority groups		White		Ethnic minority groups		White		Ethnic minority groups	
**Characteristic**	N	%	N	%	N	%	N	%	N	%	N	%	N	%	N	%	N	%	N	%
**All**	143510	91.3	20905	66.9	171190	94.1	16045	75.8	213100	95.6	11485	80.4	209385	96.2	7590	84.1	193375	97.0	5610	87.7
**Age**																				
16–64	19850	81.3	6655	59.6	19170	86.4	4720	67.5	19665	89.3	3075	72.0	17945	90.9	1970	76.4	15395	93.4	1375	81.5
65–69	12720	89.5	2560	67.0	13520	92.9	1875	75.1	15135	94.4	1275	79.1	14210	95.2	830	85.8	12005	95.9	610	86.6
70–74	22120	92.3	2425	71.5	25420	94.9	2015	79.4	31215	96.1	1535	84.2	30175	96.5	1015	87.0	26870	97.1	790	89.6
75–79	25380	93.6	2475	73.3	31565	95.6	2175	81.8	40160	96.8	1690	84.3	39405	97.4	1140	86.6	36295	97.9	920	90.3
80+	63440	94.1	6790	70.2	81520	95.4	5260	79.8	106935	96.4	3915	83.9	107650	96.8	2635	86.7	102815	97.4	1920	89.4
**Kidney disease subgroup**																				
CKD3a	92270	91.4	12630	66.3	112400	94.2	9790	75.2	142175	95.7	7210	79.8	141890	96.3	4890	83.4	132825	97.1	3710	87.4
CKD3b	37355	92.0	4675	67.0	43600	94.4	3620	77.6	53440	95.9	2530	81.7	51140	96.4	1655	85.1	46200	97.0	1170	88.1
CKD4-5	10320	90.3	1945	64.0	11610	93.1	1380	72.8	13620	94.6	980	78.9	12815	95.5	545	82.5	11245	96.3	400	85.7
RRT (dial.)	1770	83.1	850	73.2	1580	88.6	605	75.8	1540	90.8	350	81.7	1330	92.3	205	85.7	1085	93.6	140	83.8
RRT (Tx)	1800	85.2	805	72.3	2000	89.2	650	78.7	2330	92.4	415	81.7	2215	93.9	305	82.1	2015	95.1	200	85.9

Cumulative coverage (%) was determined based on Kaplan-Meier estimates, censoring at death, deregistration, or 31st August 2022. Ethnic minority groups are combined due to their low combined prevalence within the study population. CKD, chronic kidney disease; dial., dialysis; IMD, index of multiple deprivation quintile; RRT, renal replacement therapy; Tx, transplant.

Key

(salmon)	<75%
(yellow)	75–90%

For peer review only

Supplementary Table 5. Baseline characteristics of secondary outcome analysis population (dose 4 uptake).

Characteristic	All, n (%)	CKD3a, n (%)	CKD3b, n (%)	CKD4–5, n (%)	RRT (dialysis), n (%)	RRT (transplant), n (%)
**N**	698,755 (100)	431,830 (100)	199,175 (100)	51,305 (100)	3,715 (100)	12,735 (100)
**Age**						
16–64	17,035 (2.4)	5,170 (1.2)	1,405 (0.7)	565 (1.1)	615 (16.6)	9,285 (72.9)
65–69	6,630 (0.9)	3,660 (0.8)	1,075 (0.5)	335 (0.7)	145 (3.9)	1,415 (11.1)
70–74	11,015 (1.6)	6,850 (1.6)	2,200 (1.1)	650 (1.3)	145 (3.9)	1,170 (9.2)
75–79	181,200 (25.9)	130,805 (30.3)	39,940 (20.1)	8,620 (16.8)	1,225 (33.0)	615 (4.8)
80+	482,870 (69.1)	285,345 (66.1)	154,560 (77.6)	41,135 (80.2)	1,590 (42.8)	245 (1.9)
**Sex**						
Female	398,820 (57.1)	248,700 (57.6)	116,330 (58.4)	27,545 (53.7)	1,335 (35.9)	4,910 (38.6)
Male	299,940 (42.9)	183,130 (42.4)	82,845 (41.6)	23,755 (46.3)	2,385 (64.2)	7,825 (61.4)
**Ethnicity**						
White	665,365 (95.2)	413,335 (95.7)	190,370 (95.6)	48,230 (94.0)	3,080 (82.9)	10,355 (81.3)
Black	8,610 (1.2)	4,910 (1.1)	2,240 (1.1)	790 (1.5)	185 (5.0)	490 (3.8)
South Asian	18,360 (2.6)	9,860 (2.3)	4,880 (2.5)	1,770 (3.4)	360 (9.7)	1,490 (11.7)
Mixed	2,455 (0.4)	1,455 (0.3)	645 (0.3)	195 (0.4)	35 (0.9)	125 (1.0)
Other	3,965 (0.6)	2,270 (0.5)	1,045 (0.5)	320 (0.6)	55 (1.5)	275 (2.2)
**Index of multiple deprivation quintile**						
1 most deprived	105,175 (15.1)	61,455 (14.2)	31,565 (15.8)	8,785 (17.1)	765 (20.6)	2,605 (20.5)
2	127,700 (18.3)	76,800 (17.8)	37,380 (18.8)	10,065 (19.6)	805 (21.7)	2,650 (20.8)
3	160,310 (22.9)	99,115 (23.0)	45,905 (23.0)	11,735 (22.9)	805 (21.7)	2,750 (21.6)
4	157,740 (22.6)	99,470 (23.0)	44,055 (22.1)	10,980 (21.4)	720 (19.4)	2,515 (19.7)
5 least deprived	147,835 (21.2)	94,985 (22.0)	40,275 (20.2)	9,740 (19.0)	620 (16.7)	2,210 (17.4)
**Setting**						
Urban city or town	373,270 (53.4)	229,995 (53.3)	107,200 (53.8)	27,600 (53.8)	1,875 (50.5)	6,605 (51.9)
Urban conurbation	140,300 (20.1)	85,065 (19.7)	39,865 (20.0)	10,885 (21.2)	1,010 (27.2)	3,470 (27.2)
Rural	185,185 (26.5)	116,770 (27.0)	52,110 (26.2)	12,820 (25.0)	830 (22.3)	2,655 (20.8)
**Primary care coding of kidney disease**						
CKD3–5 diagnostic code	439,595 (62.9)	223,695 (51.8)	157,215 (78.9)	45,745 (89.2)	3,290 (88.6)	9,650 (75.8)
Dialysis code	11,280 (1.6)	[N]	[N]	[N]	2,980 (80.2)	8,300 (65.2)
Kidney transplant code	12,780 (1.8)	[N]	[N]	[N]	440 (11.8)	12,340 (96.9)
**Risk group (occupation/access)**						
Care home resident	44,470 (6.4)	24,280 (5.6)	15,255 (7.7)	4,705 (9.2)	165 (4.4)	65 (0.5)
Health/social care worker	1,040 (0.1)	515 (0.1)	110 (0.1)	30 (0.1)	[R]	370 (2.9)
Housebound	50,195 (7.2)	24,770 (5.7)	18,040 (9.1)	6,445 (12.6)	330 (8.9)	610 (4.8)
End of life care	36,515 (5.2)	18,305 (4.2)	12,585 (6.3)	5,110 (10.0)	335 (9.0)	180 (1.4)
**Risk group (clinical)**						
Prior SARS-CoV-2 *	20,685 (3.0)	10,845 (2.5)	6,590 (3.3)	2,295 (4.5)	415 (11.2)	535 (4.2)
Immunosuppression	56,985 (8.2)	33,045 (7.7)	13,600 (6.8)	4,000 (7.8)	985 (26.5)	5,355 (42.0)
Moderate/severe obesity	53,820 (7.7)	31,075 (7.2)	16,655 (8.4)	4,680 (9.1)	260 (7.0)	1,150 (9.0)
Diabetes	190,955 (27.3)	100,935 (23.4)	63,425 (31.8)	21,350 (41.6)	1,405 (37.8)	3,840 (30.2)
Chronic respiratory disease (inc. asthma)	92,340 (13.2)	55,060 (12.8)	28,215 (14.2)	7,570 (14.8)	505 (13.6)	990 (7.8)

For peer review only

For peer review only - http://bmjopen.bmj.com/site/about/guidelines.xhtml

Chronic heart disease	347,840 (49.8)	200,060 (46.3)	110,430 (55.4)	31,365 (61.1)	2,140 (57.6)	3,850 (30.2)
Chronic liver disease	18,560 (2.7)	11,035 (2.6)	5,265 (2.6)	1,480 (2.9)	200 (5.4)	585 (4.6)
Asplenia	5,960 (0.9)	3,690 (0.9)	1,585 (0.8)	455 (0.9)	55 (1.5)	170 (1.3)
Cancer (non-haematologic)	130,945 (18.7)	79,045 (18.3)	39,060 (19.6)	11,145 (21.7)	730 (19.7)	960 (7.5)
Haematologic cancer	20,430 (2.9)	12,180 (2.8)	5,830 (2.9)	1,875 (3.7)	310 (8.3)	240 (1.9)
Organ transplant (non-kidney) †	2,190 (0.3)	1,205 (0.3)	690 (0.3)	220 (0.4)	75 (2.0)	[N]
CND (inc. learning disability)	135,005 (19.3)	79,505 (18.4)	42,145 (21.2)	11,520 (22.5)	655 (17.6)	1,180 (9.3)
Severe mental illness	7,075 (1.0)	4,185 (1.0)	2,070 (1.0)	640 (1.2)	55 (1.5)	125 (1.0)
Clinically extremely vulnerable (other) §	5,850 (0.8)	3,585 (0.8)	1,690 (0.8)	580 (1.1)	[N]	[N]
**Region**						
East of England	156,055 (22.3)	97,360 (22.5)	43,865 (22.0)	10,950 (21.3)	910 (24.5)	2,970 (23.3)
Midlands	155,545 (22.3)	95,935 (22.2)	44,660 (22.4)	11,410 (22.2)	825 (22.2)	2,715 (21.3)
London	20,295 (2.9)	11,830 (2.7)	5,525 (2.8)	1,775 (3.5)	255 (6.9)	905 (7.1)
North East and Yorkshire	131,475 (18.8)	80,280 (18.6)	38,150 (19.2)	9,910 (19.3)	675 (18.2)	2,465 (19.4)
North West	63,585 (9.1)	39,165 (9.1)	18,285 (9.2)	4,735 (9.2)	235 (6.3)	1,160 (9.1)
South East	43,760 (6.3)	27,525 (6.4)	12,125 (6.1)	3,135 (6.1)	235 (6.3)	735 (5.8)
South West	128,045 (18.3)	79,730 (18.5)	36,560 (18.4)	9,385 (18.3)	585 (15.7)	1,780 (14.0)

Individuals were included in the analysis if they were ≥75 years of age at baseline, care home residents, transplant recipients, or had a history of haematologic malignancy or immunosuppression. Data are n (%) after rounding to the nearest 5. Primary care codes and risk groups are coded by separate binary variables; percentages under these table subheadings therefore do not sum to 100. Among people with CKD4–5, 47,670 had stage 4 CKD (eGFR 15–29 ml/min/1.73 m^2) and 3,630 had stage 5 CKD (eGFR <15ml/min/1.73 m^2). CKD, chronic kidney disease; CND, chronic neurological disease; [N], covariate absent in all individuals by definition; RRT, renal replacement therapy. * Based on prior evidence of a positive SARS-CoV-2 test, COVID-19-related primary care code, or COVID-19-related hospitalisation as of 1st December 2020; † Excludes individuals with kidney transplants based on primary care coding or UK Renal Registry status; § Classified as clinically extremely vulnerable in the absence of any of the comorbidities listed above (including RRT).

For peer review only

For peer review only - http://bmjopen.bmj.com/site/about/guidelines.xhtml

Supplementary Table 6. Cox proportional hazards models for factors associated with completion of a 4-dose vaccination series in people with kidney disease.

Variable	N (n events)	Cov. (%)	HR (95% CI), minimally adjusted	HR (95% CI), partially adjusted	HR (95% CI), fully adjusted
**Age**					
16–64 (ref)	17,035 (8,955)	56.1	1.00	1.00	1.00
65–69	6,630 (3,685)	62.3	1.18 (1.14-1.23)	1.06 (1.02-1.10)	1.53 (1.47-1.60)
70–74	11,015 (6,750)	70.5	1.41 (1.37-1.45)	1.20 (1.16-1.24)	1.89 (1.82-1.96)
75–79	181,200 (139,245)	83.9	1.71 (1.67-1.74)	1.68 (1.64-1.72)	2.96 (2.87-3.06)
80+	482,870 (321,255)	82.7	1.70 (1.67-1.74)	1.71 (1.67-1.75)	3.04 (2.94-3.13)
**Sex**					
Female (ref)	398,820 (272,245)	80.7	1.00	1.00	1.00
Male	299,940 (207,645)	83.5	1.15 (1.15-1.16)	1.14 (1.13-1.15)	1.14 (1.13-1.15)
**Ethnicity**					
White (ref)	665,365 (465,840)	83.5	1.00	1.00	1.00
Black	8,610 (3,115)	42.0	0.34 (0.33-0.35)	0.37 (0.36-0.39)	0.38 (0.37-0.40)
South Asian	18,360 (7,610)	49.2	0.42 (0.41-0.43)	0.46 (0.45-0.47)	0.46 (0.45-0.47)
Mixed	2,455 (1,185)	56.4	0.51 (0.48-0.54)	0.53 (0.50-0.56)	0.54 (0.51-0.57)
Other	3,965 (2,140)	64.7	0.66 (0.64-0.69)	0.69 (0.66-0.72)	0.68 (0.65-0.71)
**IMD**					
1 most deprived (ref)	105,175 (60,455)	70.5	1.00	1.00	1.00
2	127,700 (83,030)	78.5	1.24 (1.23-1.26)	1.19 (1.18-1.21)	1.19 (1.18-1.20)
3	160,310 (112,170)	83.3	1.41 (1.40-1.43)	1.32 (1.31-1.33)	1.31 (1.30-1.33)
4	157,740 (113,965)	85.3	1.51 (1.50-1.53)	1.41 (1.39-1.42)	1.39 (1.38-1.41)
5 least deprived	147,835 (110,270)	87.4	1.64 (1.62-1.65)	1.52 (1.50-1.53)	1.50 (1.48-1.51)
**Setting**					
Urban city or town (ref)	373,270 (258,070)	82.5	1.00	1.00	1.00
Urban conurbation	140,300 (86,560)	74.6	0.90 (0.89-0.91)	0.99 (0.98-1.00)	0.98 (0.97-0.99)
Rural town or village	185,185 (135,255)	86.2	1.10 (1.09-1.10)	1.02 (1.01-1.03)	1.02 (1.01-1.02)
**Kidney disease subgroup**					
CKD3a (ref)	431,830 (312,330)	82.9	1.00	1.00	1.00
CKD3b	199,175 (130,145)	81.4	0.92 (0.92-0.93)	0.94 (0.93-0.94)	0.93 (0.92-0.94)
CKD4–5	51,305 (27,610)	78.7	0.85 (0.84-0.86)	0.88 (0.87-0.89)	0.87 (0.86-0.88)
RRT (dialysis)	3,715 (1,830)	71.1	0.98 (0.93-1.03)	1.07 (1.02-1.12)	1.05 (1.00-1.10)
RRT (transplant)	12,735 (7,970)	67.3	2.05 (1.99-2.12)	2.37 (2.30-2.45)	2.44 (2.36-2.52)
**Primary care coding of kidney disease**					
CKD diagnostic code	439,595 (301,095)	82.2	1.02 (1.01-1.02)	1.03 (1.02-1.03)	1.05 (1.04-1.06)
**Risk group (occupation/access)**					
Care home resident	44,470 (19,620)	82.7	0.86 (0.85-0.87)	0.92 (0.90-0.93)	0.98 (0.96-0.99)
Health/social care worker	1,040 (745)	73.5	1.40 (1.30-1.51)	1.45 (1.35-1.56)	1.40 (1.30-1.51)
Housebound	50,195 (25,025)	77.2	0.72 (0.72-0.73)	0.75 (0.74-0.76)	0.77 (0.76-0.78)
End of life care	36,515 (15,080)	79.1	0.89 (0.87-0.90)	0.92 (0.90-0.93)	0.92 (0.90-0.94)
**Risk group (clinical)**					
Prior SARS-CoV-2	20,685 (9,985)	75.8	0.86 (0.84-0.88)	0.91 (0.89-0.93)	0.92 (0.90-0.94)
Immunosuppression	56,985 (35,705)	74.3	1.20 (1.19-1.22)	1.16 (1.14-1.18)	1.19 (1.18-1.21)
Moderate/severe obesity	53,820 (36,900)	78.9	0.89 (0.88-0.89)	0.93 (0.92-0.94)	0.94 (0.93-0.96)
Diabetes	190,955 (122,995)	79.1	0.88 (0.88-0.89)	0.94 (0.93-0.94)	0.95 (0.94-0.95)
CRD (inc. asthma)	92,340 (58,055)	80.8	0.97 (0.96-0.97)	0.98 (0.97-0.99)	0.98 (0.97-0.99)
Chronic heart disease	347,840 (229,640)	82.4	0.99 (0.98-0.99)	0.97 (0.97-0.98)	0.98 (0.98-0.99)
Chronic liver disease	18,560 (12,195)	78.9	1.00 (0.99-1.02)	1.02 (1.00-1.04)	1.02 (1.00-1.04)
Asplenia	5,960 (4,170)	83.2	1.10 (1.07-1.14)	1.08 (1.05-1.12)	1.08 (1.04-1.11)
Cancer (non-haematologic)	130,945 (88,215)	84.6	1.09 (1.09-1.10)	1.06 (1.05-1.07)	1.07 (1.06-1.07)
Haematologic cancer	20,430 (12,960)	79.2	1.29 (1.27-1.31)	1.13 (1.11-1.16)	1.17 (1.14-1.19)
Organ transplant (non-kidney)	2,190 (1,430)	73.2	1.51 (1.43-1.59)	1.56 (1.48-1.64)	1.99 (1.88-2.10)
CND (inc. learning disability)	135,005 (80,650)	80.4	0.89 (0.89-0.90)	0.90 (0.89-0.91)	0.90 (0.90-0.91)
Severe mental illness	7,075 (3,795)	69.3	0.70 (0.68-0.72)	0.75 (0.72-0.77)	0.77 (0.74-0.79)
CEV (other)	5,850 (4,005)	80.9	1.02 (0.99-1.05)	1.07 (1.04-1.10)	1.03 (1.00-1.07)

Cumulative coverage was determined based on Kaplan-Meier estimates, censoring at death, deregistration, or 31st August 2022. Minimally adjusted models included age, care home residence, and health and social care worker status given their use in vaccine prioritisation criteria. Partially adjusted models additionally included housebound status, receipt of end-of-life care, setting, sex, ethnicity, IMD quintile, prior SARS-CoV-2 infection, immunosuppression, and haematologic cancer. Fully adjusted models included all covariates. CI, confidence interval; CKD, chronic kidney disease; HR, hazard ratio; IMD, index of multiple deprivation; OR, odds ratio; RRT, renal replacement therapy.

For peer review only - http://bmjopen.bmj.com/site/about/guidelines.xhtml

Supplementary Table 7. Cox proportional hazards models for factors associated with completion of a 4-dose vaccination series in kidney disease subgroups.

	CKD3a		CKD3b		CKD4-5		RRT (dialysis)		RRT (transplant)	
**Variable**	**Cov. (%)**	**HR (95% CI)**	**Cov. (%)**	**HR (95% CI)**	**Cov. (%)**	**HR (95% CI)**	**Cov. (%)**	**HR (95% CI)**	**Cov. (%)**	**HR (95% CI)**
**Age**										
16–64 (ref)	47.4	1.00	51.1	1.00	54.7	1.00	51.8	1.00	61.9	1.00
65–69	55.6	1.19 (1.12-1.27)	61.0	1.23 (1.10-1.38)	62.1	1.26 (1.03-1.53)	61.6	1.27 (0.97-1.67)	80.4	1.63 (1.53-1.75)
70–74	68.4	1.62 (1.54-1.70)	70.0	1.44 (1.31-1.59)	68.1	1.31 (1.11-1.55)	71.8	1.49 (1.13-1.96)	84.3	1.72 (1.60-1.85)
75–79	84.7	2.69 (2.58-2.82)	82.1	2.10 (1.93-2.27)	79.0	1.73 (1.52-1.97)	75.3	1.64 (1.32-2.05)	87.1	1.72 (1.56-1.90)
80+	83.5	2.78 (2.66-2.90)	81.8	2.16 (2.00-2.34)	79.3	1.74 (1.53-1.99)	76.6	1.77 (1.42-2.20)	83.1	1.75 (1.49-2.06)
**Sex**										
Female (ref)	81.7	1.00	79.9	1.00	76.6	1.00	66.6	1.00	69.0	1.00
Male	84.7	1.13 (1.12-1.14)	83.5	1.16 (1.15-1.18)	81.2	1.19 (1.16-1.21)	73.0	1.14 (1.03-1.26)	66.1	0.92 (0.88-0.96)
**Ethnicity**										
White (ref)	84.4	1.00	82.9	1.00	80.8	1.00	75.5	1.00	72.9	1.00
Black	41.8	0.36 (0.35-0.38)	42.5	0.39 (0.36-0.41)	42.3	0.41 (0.37-0.47)	50.1	0.57 (0.44-0.72)	35.1	0.39 (0.34-0.46)
South Asian	51.1	0.46 (0.44-0.47)	48.3	0.45 (0.43-0.47)	44.2	0.44 (0.40-0.48)	49.1	0.54 (0.45-0.65)	43.1	0.51 (0.47-0.56)
Mixed	58.8	0.56 (0.52-0.60)	55.5	0.52 (0.47-0.59)	45.3	0.47 (0.37-0.60)	[R]	[R]	39.1	0.49 (0.36-0.65)
Other	65.2	0.66 (0.63-0.70)	65.0	0.68 (0.63-0.74)	63.9	0.79 (0.67-0.93)	[R]	[R]	54.0	0.63 (0.53-0.75)
**IMD**										
1 most deprived (ref)	71.9	1.00	71.1	1.00	66.7	1.00	54.9	1.00	48.7	1.00
2	79.7	1.20 (1.18-1.21)	78.1	1.17 (1.15-1.20)	75.5	1.19 (1.14-1.25)	68.6	1.37 (1.18-1.60)	61.1	1.35 (1.25-1.46)
3	84.0	1.31 (1.30-1.33)	83.1	1.30 (1.28-1.33)	80.1	1.31 (1.25-1.36)	76.1	1.47 (1.26-1.72)	71.6	1.65 (1.53-1.78)
4	86.1	1.40 (1.38-1.42)	84.7	1.38 (1.35-1.40)	83.2	1.42 (1.36-1.48)	78.8	1.56 (1.33-1.83)	76.6	1.82 (1.69-1.97)
5 least deprived	88.1	1.51 (1.49-1.53)	86.6	1.48 (1.45-1.51)	85.4	1.52 (1.46-1.58)	77.4	1.53 (1.30-1.80)	80.0	1.98 (1.84-2.15)
**Setting**										
Urban city or town (ref)	83.4	1.00	82.1	1.00	79.3	1.00	70.7	1.00	68.2	1.00
Urban conurbation	76.1	0.99 (0.98-1.00)	74.0	0.97 (0.95-0.98)	70.9	1.01 (0.98-1.05)	64.3	1.15 (1.00-1.32)	56.9	1.05 (0.98-1.13)
Rural town or village	86.9	1.02 (1.01-1.03)	85.4	1.01 (1.00-1.02)	83.8	1.04 (1.01-1.07)	80.2	1.09 (0.97-1.23)	78.6	1.13 (1.07-1.20)
**Primary care coding of kidney disease**										
CKD diagnostic code	83.6	1.03 (1.03-1.04)	82.0	1.08 (1.06-1.09)	79.0	1.08 (1.04-1.13)	72.4	1.10 (0.95-1.29)	69.0	1.15 (1.09-1.21)
**Risk group (occupation/access)**										
Care home resident	82.4	0.91 (0.89-0.92)	83.6	0.96 (0.94-0.99)	81.8	1.05 (1.00-1.11)	57.1	0.83 (0.62-1.12)	50.0	0.58 (0.40-0.83)
Health/social care worker	73.2	1.42 (1.28-1.57)	76.1	1.37 (1.11-1.70)	[R]	[R]	[R]	[R]	71.2	1.31 (1.15-1.48)
Housebound	77.8	0.74 (0.73-0.75)	77.6	0.77 (0.75-0.78)	74.2	0.78 (0.75-0.81)	68.3	0.93 (0.77-1.12)	72.6	0.96 (0.87-1.07)
End of life care	80.2	0.91 (0.89-0.93)	79.2	0.93 (0.90-0.96)	74.8	0.91 (0.86-0.96)	63.1	0.77 (0.64-0.94)	73.4	1.23 (1.00-1.51)
**Risk group (clinical)**										
Prior SARS-CoV-2	77.8	0.91 (0.89-0.94)	77.0	0.92 (0.89-0.95)	71.6	0.88 (0.82-0.95)	56.4	0.77 (0.65-0.91)	54.1	0.84 (0.74-0.95)
Immunosuppression	74.1	1.19 (1.17-1.22)	77.3	1.19 (1.15-1.23)	74.6	1.27 (1.19-1.36)	60.6	1.12 (0.91-1.37)	70.6	1.18 (1.13-1.24)
Moderate/severe obesity	79.8	0.93 (0.92-0.94)	79.2	0.94 (0.93-0.96)	75.8	0.94 (0.90-0.98)	62.3	0.90 (0.74-1.09)	64.5	1.00 (0.92-1.08)
Diabetes	79.9	0.93 (0.92-0.94)	79.3	0.96 (0.95-0.97)	76.8	0.97 (0.95-0.99)	68.3	0.96 (0.87-1.06)	67.5	1.05 (1.00-1.11)
CRD (inc. asthma)	81.6	0.98 (0.97-0.99)	80.3	0.98 (0.97-1.00)	78.1	1.00 (0.97-1.04)	72.3	1.04 (0.90-1.20)	68.3	0.95 (0.87-1.03)
Chronic heart disease	83.4	0.97 (0.97-0.98)	81.7	0.98 (0.97-0.99)	79.2	1.00 (0.97-1.02)	73.4	1.02 (0.93-1.13)	72.4	1.06 (1.01-1.11)
Chronic liver disease	79.5	1.03 (1.01-1.05)	79.2	1.03 (1.00-1.07)	76.1	1.03 (0.96-1.11)	66.6	1.19 (0.97-1.47)	69.5	1.19 (1.07-1.32)
Asplenia	84.5	1.09 (1.05-1.14)	83.4	1.10 (1.03-1.17)	75.3	1.01 (0.89-1.14)	[R]	[R]	63.8	0.93 (0.76-1.13)
Cancer (non-haematologic)	85.3	1.07 (1.06-1.08)	83.8	1.06 (1.05-1.08)	82.0	1.08 (1.05-1.11)	78.3	1.09 (0.97-1.22)	78.7	1.16 (1.07-1.26)
Haematologic cancer	79.0	1.17 (1.14-1.21)	80.3	1.17 (1.12-1.22)	77.3	1.13 (1.04-1.23)	72.2	1.38 (1.13-1.69)	73.4	1.00 (0.86-1.18)
Organ transplant (non-kidney)	72.5	2.08 (1.94-2.23)	74.1	1.86 (1.68-2.05)	69.0	1.52 (1.27-1.82)	61.5	1.63 (1.18-2.24)	[N]	[N]
CND (inc. learning disability)	81.2	0.90 (0.89-0.91)	79.9	0.90 (0.88-0.91)	76.9	0.92 (0.89-0.95)	67.5	0.90 (0.79-1.02)	71.4	1.04 (0.97-1.12)
Severe mental illness	68.4	0.74 (0.71-0.77)	71.6	0.78 (0.74-0.83)	71.4	0.87 (0.78-0.97)	35.2	0.58 (0.37-0.92)	54.3	0.87 (0.68-1.12)

CEV (other)	81.1	1.06 (1.02-1.11)	80.5	1.11 (1.05-1.18)	77.9	1.06 (0.95-1.18)	[N]	[N]	[N]	[N]

Individuals were included in the analysis if they were ≥75 years of age at baseline, care home residents, transplant recipients, or had a history of haematologic malignancy or immunosuppression. Counts are rounded to the nearest 5. Cumulative coverage was determined based on Kaplan-Meier estimates, censoring at death, deregistration, or 31st August 2022. Hazard ratios and 95% confidence intervals were derived from partially adjusted models that included age, care home residence, health and social care worker status, housebound status, receipt of end-of-life care, setting (urban/rural), sex, ethnicity, IMD quintile, prior SARS-CoV-2 infection, immunosuppression, and haematologic cancer. Data are redacted [R] for any rows in which there were >0 and ≤10 event or non-event counts after rounding to the nearest 5, with non-event counts calculated among uncensored individuals. CEV, clinically extremely vulnerable; CKD, chronic kidney disease; CND, chronic neurological disease; IMD, index of multiple deprivation; [N], covariate absent in all individuals by definition; [R] redacted; RRT, renal replacement therapy.

For peer review only

Supplementary Table 8. Cumulative 4-dose coverage in subgroups defined by deprivation index, ethnicity, age, and kidney disease severity.

	IMD 1 (most deprived)				IMD 2				IMD 3				IMD 4				IMD 5 (least deprived)			
	White		Ethnic minority groups		White		Ethnic minority groups		White		Ethnic minority groups		White		Ethnic minority groups		White		Ethnic minority groups	
**Characteristic**	N	%	N	%	N	%	N	%	N	%	N	%	N	%	N	%	N	%	N	%
**All**	94295	74.8	10880	35.3	119075	80.8	8620	47.8	153940	84.4	6370	56.5	153430	86.0	4310	62.8	144625	87.8	3205	70.8
**Age**																				
16–64	2805	47.0	1190	24.6	2860	58.5	815	32.0	3105	62.4	505	40.1	2830	66.6	340	42.7	2360	71.8	235	49.9
65–69	1030	54.1	235	29.1	1130	60.0	205	38.1	1340	64.9	130	43.1	1275	69.5	100	47.4	1120	72.9	65	66.7
70–74	1635	61.0	190	36.2	2000	70.2	165	39.2	2400	73.8	135	44.1	2270	74.0	100	60.9	2040	77.3	70	63.1
75–79	25380	76.4	2475	37.5	31565	82.5	2175	52.0	40160	86.2	1690	57.3	39405	88.0	1140	65.3	36295	89.7	920	73.3
80+	63440	76.2	6790	36.5	81520	81.6	5260	49.2	106935	84.9	3915	59.0	107650	86.2	2635	64.5	102815	87.8	1920	72.1
**Kidney disease subgroup**																				
CKD3a	55615	75.7	5845	36.3	72130	81.7	4670	49.1	95515	85.0	3600	57.3	97010	86.7	2460	63.6	93065	88.4	1920	70.7
CKD3b	28670	74.9	2890	35.2	35050	80.3	2330	46.5	44240	84.1	1665	57.9	42920	85.3	1130	62.8	39485	86.9	790	72.7
CKD4-5	7685	72.0	1105	30.8	9260	77.8	805	50.0	11155	81.6	580	51.0	10635	84.0	345	60.3	9500	85.9	235	67.7
RRT (dial.)	530	60.6	235	40.3	640	73.3	165	46.5	695	79.7	110	52.7	650	80.4	70	64.1	565	78.4	60	55.6
RRT (Tx)	1800	56.7	805	30.4	2000	67.8	650	39.7	2330	75.2	415	49.8	2215	79.5	305	54.2	2015	81.4	200	63.3

Cumulative coverage (%) was determined based on Kaplan-Meier estimates, censoring at death, deregistration, or 31st August 2022. Ethnic minority groups are combined due to their low combined prevalence within the study population. CKD, chronic kidney disease; dial., dialysis; IMD, index of multiple deprivation quintile; RRT, renal replacement therapy; Tx, transplant.

Key

(orange)	<50%
(yellow)	50–75%

For peer review only

STROBE Statement—checklist of items that should be included in reports of observational studies

	Item No.	Recommendation	Page No.	Relevant text from manuscript
**Title and abstract**	1	(*a*) Indicate the study's design with a commonly used term in the title or the abstract	1–2	"cohort study" referred to in title and abstract
		(*b*) Provide in the abstract an informative and balanced summary of what was done and what was found	2	N/A
**Introduction**				
Background/rationale	2	Explain the scientific background and rationale for the investigation being reported	3	N/A
Objectives	3	State specific objectives, including any prespecified hypotheses	3	Knowledge gaps addressed by current study identified in paragraph 3 of Background.
**Methods**				
Study design	4	Present key elements of study design early in the paper	3	Summary of approach provided in paragraph 4 of Background.
Setting	5	Describe the setting, locations, and relevant dates, including periods of recruitment, exposure, follow-up, and data collection	3–4 & Table 1	'Data sources' subheading provides details of databases supporting analyses. 'Study population' subheading defines relevant dates. 'Outcomes' subheading defines relevant exposures. Table 1 provides a breakdown of the study population by location.
Participants	6	(*a*) *Cohort study*—Give the eligibility criteria, and the sources and methods of selection of participants. Describe methods of follow-up<br>*Case-control study*—Give the eligibility criteria, and the sources and methods of case ascertainment and control selection. Give the rationale for the choice of cases and controls<br>*Cross-sectional study*—Give the eligibility criteria, and the sources and methods of selection of participants	4 & Supp Fig 1	Inclusion criteria are defined under 'Study population' subheading and in Supp Fig 1, which provides a flowchart of cohort selection. Databases are defined under the 'Data sources' subheading.
		(*b*) *Cohort study*—For matched studies, give matching criteria and number of exposed and unexposed	N/A	No matching undertaken.

For peer review only - http://bmjopen.bmj.com/site/about/guidelines.xhtml

		Case-control study—For matched studies, give matching criteria and the number of controls per case		
Variables	7	Clearly define all outcomes, exposures, predictors, potential confounders, and effect modifiers. Give diagnostic criteria, if applicable	4–5	Outcomes are defined under 'Outcomes' subheading. All covariates included in models are defined under 'Covariates' subheading. The sequential adjustment process adopted to account for potential confounding is defined in the first paragraph of the 'Statistical analysis' subheading.
Data sources/ measurement	8*	For each variable of interest, give sources of data and details of methods of assessment (measurement). Describe comparability of assessment methods if there is more than one group	3	All variables were defined using the OpenSAFELY-TPP database described under the 'Data sources' subheading.
Bias	9	Describe any efforts to address potential sources of bias	4–5	The sequential adjustment process adopted to account for potential confounding is defined under the first paragraph of the 'Statistical analysis' subheading. Exclusion of individuals with missing demographic data (which is often an indicator of poor data quality in electronic health records) is described in paragraph 1 under the 'Study population' subheading.
Study size	10	Explain how the study size was arrived at	Supp Fig 1	Supp Fig 1 provides a full flow chart of the study selection process
Quantitative variables	11	Explain how quantitative variables were handled in the analyses. If applicable, describe which groupings were chosen and why	4	Definition of chronic kidney disease stage in relation to eGFR followed standard diagnostic thresholds, as described in paragraph 3 of the 'Study population' subheading. Age cut-offs were defined based on vaccine eligibility criteria, as described in paragraph 1 under the 'Covariates' subheading.
Statistical methods	12	(*a*) Describe all statistical methods, including those used to control for confounding	5	The sequential adjustment process adopted to account for potential confounding is defined under

For peer review only

				the first paragraph of the 'Statistical analysis' subheading.
		(*b*) Describe any methods used to examine subgroups and interactions	5	Subgroup analyses are described in paragraph 2 under the 'Statistical analysis' subheading.
		(*c*) Explain how missing data were addressed	4	Exclusion of individuals with missing demographic data (which is often an indicator of poor data quality in electronic health records) is described in paragraph 1 under the 'Study population' subheading. Data were complete for all other covariates.
		(*d*) *Cohort study*—If applicable, explain how loss to follow-up was addressed<br>*Case-control study*—If applicable, explain how matching of cases and controls was addressed<br>*Cross-sectional study*—If applicable, describe analytical methods taking account of sampling strategy	5	As noted in paragraph 1 under the 'Statistical analysis' subheading, we censored at death, deregistration, or the end of the study period (11th May 2022).
		(*e*) Describe any sensitivity analyses	5	The logistic regression approach used as a sensitivity analysis is described in paragraph 2 under this subheading.
**Results**				
Participants	13*	(a) Report numbers of individuals at each stage of study—eg numbers potentially eligible, examined for eligibility, confirmed eligible, included in the study, completing follow-up, and analysed	Supp Fig 1 and Supp Table 2	Supp Fig 1 provides a full flow chart of the study selection process. The number of individuals uncensored at the end of follow-up is defined in Supp Table 2 (penultimate column).
		(b) Give reasons for non-participation at each stage	N/A	N/A
		(c) Consider use of a flow diagram	Supp Fig 1	Supp Fig 1 provides a full flow chart of the study selection process.
Descriptive data	14*	(a) Give characteristics of study participants (eg demographic, clinical, social) and information on exposures and potential confounders	Table 1 and Supp Table 4	Table 1 and Supp Table 4 provide a summary of the covariate profile for the study population as a whole and analysis subgroups.
		(b) Indicate number of participants with missing data for each variable of interest	N/A	Data were complete for all covariates in the final study population.

For peer review only - http://bmjopen.bmj.com/site/about/guidelines.xhtml
For peer review only

		(c) *Cohort study*—Summarise follow-up time (eg, average and total amount)	Fig 1	Fig 1A provides a summary of the event distribution for each outcome over the course of follow-up. The analysis cut-off (11th May 2022) is specified in the figure legend and elsewhere in the manuscript.
Outcome data	15*	*Cohort study*—Report numbers of outcome events or summary measures over time	Supp Tables 2, 3, 5, and 6	Supp Tables provide a breakdown of the number of events by subgroup for all analyses
		Case-control study—Report numbers in each exposure category, or summary measures of exposure	N/A	N/A
		Cross-sectional study—Report numbers of outcome events or summary measures	N/A	N/A
Main results	16	(*a*) Give unadjusted estimates and, if applicable, confounder-adjusted estimates and their precision (eg, 95% confidence interval). Make clear which confounders were adjusted for and why they were included	Supp Tables 2 and 5	Supp Tables 2 and 5 present results of sequentially adjusted models with 95% CIs. The variables included in the models are indicated via the symbols * (minimally adjusted models) and † (partially adjusted models)
		(*b*) Report category boundaries when continuous variables were categorized	Table 1 and Supp Tables 2–6	The boundaries used to define age groups are specified in all results tables. No other covariates required category boundaries to be specified.
		(*c*) If relevant, consider translating estimates of relative risk into absolute risk for a meaningful time period	N/A	N/A
Other analyses	17	Report other analyses done—eg analyses of subgroups and interactions, and sensitivity analyses	Supp Tables 2, 3, and 6	Subgroup analyses are summarised in Supp Tables 3 and 6. Sensitivity analyses (via logistic regression) are summarised in Supp Table 2.
**Discussion**				
Key results	18	Summarise key results with reference to study objectives	7	'Summary' subheading
Limitations	19	Discuss limitations of the study, taking into account sources of potential bias or imprecision. Discuss both direction and magnitude of any potential bias	8	'Strengths and limitations' subheading

For peer review only - http://bmjopen.bmj.com/site/about/guidelines.xhtml

Interpretation	20	Give a cautious overall interpretation of results considering objectives, limitations, multiplicity of analyses, results from similar studies, and other relevant evidence	7–8	‘Findings in context’ subheading
Generalisability	21	Discuss the generalisability (external validity) of the study results	9	‘Policy implications and interpretation’ subheading
**Other information**				
Funding	22	Give the source of funding and the role of the funders for the present study and, if applicable, for the original study on which the present article is based	9	‘Funding’ subheading

*Give information separately for cases and controls in case-control studies and, if applicable, for exposed and unexposed groups in cohort and cross-sectional studies.

Note: An Explanation and Elaboration article discusses each checklist item and gives methodological background and published examples of transparent reporting. The STROBE checklist is best used in conjunction with this article (freely available on the Web sites of PLoS Medicine at http://www.plosmedicine.org/, Annals of Internal Medicine at http://www.annals.org/, and Epidemiology at http://www.epidem.com/). Information on the STROBE Initiative is available at www.strobe-statement.org.

For peer review only

For peer review only - http://bmjopen.bmj.com/site/about/guidelines.xhtml
